# Fluoroquinolone-Based Organic Salts (GUMBOS) with Antibacterial Potential

**DOI:** 10.3390/ijms242115714

**Published:** 2023-10-28

**Authors:** Fábio M. S. Costa, Andreia Granja, Rocío L. Pérez, Isiah M. Warner, Salette Reis, Marieta L. C. Passos, M. Lúcia M. F. S. Saraiva

**Affiliations:** 1 LAQV, REQUIMTE, Laboratory of Applied Pharmacy, Department of Chemical Sciences, Faculty of Pharmacy, Porto University, Rua de Jorge Viterbo Ferreira, 4050-313 Porto, Portugal; up201206985@up.pt (F.M.S.C.); aagranja@ff.up.pt (A.G.); shreis@ff.up.pt (S.R.); 2 Department of Chemistry, Louisiana State University, Baton Rouge, LA 70803, USA; rperez@georgiasouthern.edu (R.L.P.); iwarner@lsu.edu (I.M.W.); 3 Department of Chemistry and Biochemistry, Georgia Southern University, Statesboro, GA 30458, USA; 4 Department of Chemistry, Cincinnati University, Cincinnati, OH 45221, USA

**Keywords:** ciprofloxacin, moxifloxacin, fluoroquinolones, GUMBOS, antibacterial, combination drug therapy

## Abstract

Antimicrobial resistance is a silent pandemic considered a public health concern worldwide. Strategic therapies are needed to replace antibacterials that are now ineffective. One approach entails the use of well-known antibacterials along with adjuvants that possess non-antibiotic properties but can extend the lifespan and enhance the effectiveness of the treatment, while also improving the suppression of resistance. In this regard, a group of uniform materials based on organic salts (GUMBOS) presents an alternative to this problem allowing the combination of antibacterials with adjuvants. Fluoroquinolones are a family of antibacterials used to treat respiratory and urinary tract infections with broad-spectrum activity. Ciprofloxacin and moxifloxacin-based GUMBOS were synthesized via anion exchange reactions with lithium and sodium salts. Structural characterization, thermal stability and octanol/water partition ratios were evaluated. The antibacterial profiles of most GUMBOS were comparable to their cationic counterparts when tested against Gram-positive *S. aureus* and Gram-negative *E. coli*, except for deoxycholate anion, which demonstrated the least effective antibacterial activity. Additionally, some GUMBOS were less cytotoxic to L929 fibroblast cells and non-hemolytic to red blood cells. Therefore, these agents exhibit promise as an alternative approach to combining drugs for treating infections caused by resistant bacteria.

## 1. Introduction

Antimicrobial resistance (AMR) arises when pathogens become unresponsive to the effects of the available medication, and it has become a healthcare crisis. While this is a natural phenomenon, the appearance and dissemination of drug-resistant organisms are accelerated by inappropriate overdosing, arbitrary prescribing, lack of clean water and poor hygiene in communities, unnecessary use in agriculture and cattle, inadequate infection prevention protocols, poor diagnostic and public health infrastructure, lack of funding and gaps in knowledge and education [1].

Antibiotics are becoming ineffective against bacteria, due to their inherent resistant strategies such as modifications of drugs influx or efflux systems, enzymatic degradation, and alterations in target locations [2]. Moreover, they are becoming ineffective due to human action through misuse and overuse of antibacterials [1] which leads to an increased dosage use or change of antibiotics class in the clinical setting [3]. In the market setting, most novel drugs being approved are minimal modifications of identified classes instead of new ones which can be a cause for further resistance [4]. Thus, humanity is vulnerable as the existing antibiotics and traditional combinations are not successful anymore against multi-drug resistance (MDR) and there is a need to search for the sources of this crisis and for alternative therapies that potentiate the activity of antibiotics [5]. Adjuvants can be unconventional or prescription-only non-antibiotic drugs with minimal or no antibacterial activity; however, they can improve pharmacological properties such as the extension of antibiotic lifetime and enhancement of antibiotic potency or resistance suppression [6]. Other current alternatives include synergic activity observed between different drugs, resistance inhibition, and drugs that modify the physiology of resistant cells [7]. The implementation of these novel methodologies is still in the early stages, and there is difficulty in effectively converting the results obtained in the laboratory set-up into the best possible outcomes when applied in a clinical setting [8], mainly because of the variability of the pharmacokinetic parameters [9].

Research has been enthusiastic about the utilization of ionic liquids (ILs) and a group of uniform materials based on organic salts (GUMBOS) as an alternative that could fix complications seen with traditional therapies, as they possess versatility and high tunability among several of their properties [10]. In the literature, organic salts derived from active pharmaceutical ingredients (APIs) also fall into this category of GUMBOS as they allow the pairing of charged entities through basic chemical reactions to obtain a product with tunable properties, such as broad-spectrum action towards pathogens [11,12]. Organic salts that have been researched as potential pharmaceutical agents with good performances are chlorhexidine derivatives against enterohaemorrhagic *E. coli* [13] and several other bacterial species [14,15], octenidine-penicillin GUMBOS against gonorrhea-causing *Neisseria gonorrhoeae* [16], amphotericin B Ils and organic salts against infantile visceral leishmaniasis-causing *Leishmania infantum* [17] and clofazimine-fluoroquinolones organic salts against *Mycobacterium avium* [18]. The majority of these studies used anion exchange reactions for the combination of different active pharmaceutical ingredients [13,14,16,19,20,21,22]. Other studies also used an acid-base neutralization method on fluoroquinolones organic salts as anions with clofazimine, an antimycobacterial drug, with improved solubility and thermostability, without significant damage to the drug’s bioactivity properties [18].

**Figure 1:**
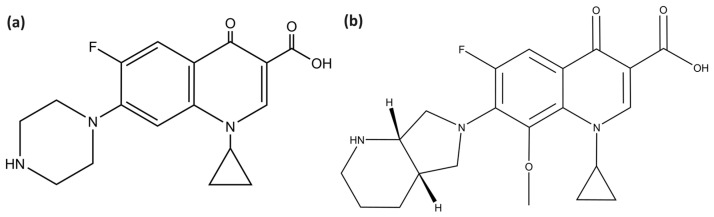
Structures of studied fluoroquinolones: ciprofloxacin (**a**) and moxifloxacin (**b**).

Quinolones are synthetic antimicrobials widely used nowadays with multiple biomedical applications. Nalidixic acid discovery [23] steered the development of a second-generation category called fluoroquinolones (FQ) after insertion of a fluorine atom and a primary ring substitution such as norfloxacin in 1986 and ciprofloxacin (Figure 1a) in 1987 [24]. Fluoroquinolones have a broad-spectrum antibacterial performance [25] and have been extensively used to treat bacterial infections [26]. Nowadays, ciprofloxacin is amongst the most common FQ in the clinic, specifically to treat urinary tract infections caused by Gram-negative bacteria, and after more than thirty years, it is still regarded as a standard antibiotic [27]. Subsequently, several fluoroquinolones have been developed such as levofloxacin from the third generation and gemifloxacin and moxifloxacin (Figure 1b) from the fourth generation as potent antibacterials against Gram-positive and anaerobic bacteria [25,28]. Functional groups of ciprofloxacin include cyclopropyl, carboxylic acid, fluoro, and piperazin-1-yl [29], while moxifloxacin has a (4aS,7aS)-octahydro-6H-pyrrolo [3,4-b]yridine-6-yl group instead of the piperazin-1-yl and an additional methoxy substituent, which is reported to increase potency and decrease toxicity [30]. Ciprofloxacin and moxifloxacin are both zwitterionic at physiological pH [31,32] with good solubility in acidic or basic solvents, while its solubility in organic solvents is poor. The solubility of zwitterionic ciprofloxacin can be improved by combining it with other cations, such as sodium or potassium or anions, such as chloride or sulfate. Additionally, ciprofloxacin can also be formulated into different salt forms to improve its solubility.

The mechanism of fluoroquinolones involves interference in bacterial enzymes, specifically DNA gyrase and topoisomerase IV, which could lead to the inhibition of bacterial DNA synthesis [33]. Nevertheless, as a result of widespread usage of these antimicrobial agents in treatments, the resistance rate of FQ has increased in a wide range of bacterial species [34]. FQ resistance is widely credited to chromosomal mutations of specific genes or mutations causing reduced drug accumulation, and the existence of plasmid-mediated quinolone resistance genes [24]. By combining zwitterionic fluoroquinolones with other active pharmaceutical ingredients, innovative compounds can be created to develop combination therapies. These therapies have the potential to offer enhanced effectiveness and broader activity against various pathogens that have exhibited resistance to FQ. In the literature, FQ-based organic salts with antibacterial performances have been synthesized using fluoroquinolones as cations with non-steroidal anti-inflammatory drugs (NSAIDs) [35], a non-denaturing detergent [36], carboxylic acids [37,38], specifically fatty acids [39,40] and amino acids [41,42]. More recently, FQ have been used in synthesis as anions with choline and ammonium, pyridinium and N-methylimidazolim ILs [22,43].

The main aim of this work is to synthesize novel fluoroquinolone-based GUMBOS and evaluate their physicochemical and biological properties to employ them as alternatives to conventional drug therapies to benefit humanity in the evolving antimicrobial resistance global crisis.

## 2. Results and Discussion

The objective of this study was to develop GUMBOS and assess their structural and thermal characteristics, as well as investigate their antibacterial properties, cytotoxicity, and hemocompatibility. Ciprofloxacin and moxifloxacin-derived GUMBOS were produced, and their physicochemical characterization was performed.

### 2.1. Synthesis of GUMBOS

**Figure 2:**
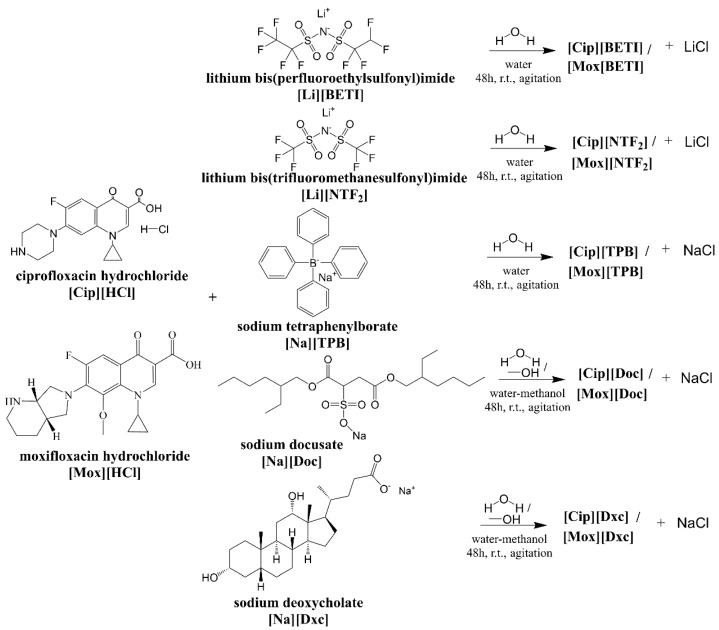
Synthesis of ciprofloxacin-based and moxifloxacin-based GUMBOS.

Ciprofloxacin and moxifloxacin-based GUMBOS were produced through simple anion exchange reactions (Figure 2). 

Yields obtained for each of the GUMBOS are described in Table 1, ranging between 48–81%. Similar yield values were obtained when conjugating these fluoroquinolones with fatty acids using amides chemistry [40]. Some of the yield differences obtained may be attributed to chemical reactivity and purity of starting materials, stoichiometry, reaction conditions, solvent choice, crystallization, and purification techniques. In general, yields for [Cip]-based GUMBOS were higher than [Mox]-based GUMBOS. Anion selection may have an effect in yield results as GUMBOS with [TPB] and [Dxc] anions had the lowest yields while the remaining anions resulted in relatively higher yields.

### 2.2. Structural Characterization

Physicochemical characterization through NMR, FT-IR spectroscopy, and ESI-MS verified the presence of both the positively charged cation and the negatively charged anion components.

#### 2.2.1. ^1^H- and ^13^C-NMR

Proton and carbon peaks were respectively assigned to each atom in the structure of each GUMBOS. Precursors were also analyzed to serve as a comparison method and validation of the results obtained. ^1^H- and ^13^C-NMR spectra can be found in the Supplementary Materials file. A comparative analysis confirmed bonding of cation and anion entities, owing to the appearance of significant changes in GUMBOS’ spectra, in relation to the same signals in the spectra of the parent substances. 

Characteristic proton bands [44] in the ^1^H-NMR spectra of [Cip]-based GUMBOS at 15.11 ppm (COOH), 8.68–7.61 ppm (CH groups from quinolin-4-one), 3.86 ppm (CH from cyclopropyl), 3.60–3.31 ppm (CH_2_ groups from piperazinium) and 1.36–1.15 ppm (CH_2_ groups from cyclopropyl). It also shows the disappearance of the proton band of the NH^+^ group (at 9.41 ppm) in the piperazinium ring, which is present in [Cip][HCl] [44]. This fact supports the assumption that [Cip] is present in GUMBOS in its non-ionised form. ^13^C-NMR spectra show characteristic carbon bands of ciprofloxacin [44] at 176.41 ppm (C=O), 165.85 ppm (COOH), 154.13, 148.21, 144.19, 139.10, 119.36, 111.19 and 106.88 ppm (carbon atoms at quinoline-4-one ring), 46.36 and 42.50 ppm (CH_2_ groups from piperazinium) and 35.99 and 7.62 ppm (CH and CH_2_ groups from cyclopropyl, respectively).

^1^H-NMR spectra of [Mox]-based GUMBOS had the following characteristic proton bands [45] at 15.11 ppm (COOH), 8.65 and 7.61 ppm (CH_2_ groups from 4-oxo-1,4-dihydroquinoline-3-carboxylic acid), 4.16 ppm (CH from cyclopropyl), 4.08–3.65 ppm (CH_2_ groups from (4aS,7aS)-octahydro-6H-pyrrolo [3,4-b]pyridin-6-yl)), 3.61 ppm (CH_3_ from methoxy), 3.18–1.70 ppm (CH_2_ and CH groups from (4aS,7aS)-octahydro-6H-pyrrolo [3,4-b]pyridin-6-yl)) and 1.21–0.89 (CH_2_ groups from cyclopropyl). It also shows the disappearance of the proton band of the NH^+^ group (at 9.99 ppm) in the piperidinopyrrolidine appendage, which is present in [Mox][HCl]. Characteristic carbon bands of moxifloxacin [45] in the ^13^C-NMR spectra were observed at 176.01 ppm (C=O), 165.84 ppm (C=O), 153.72–151.42 ppm (C-F), 150.36, 136.68, 134.51, 117.22 and 106.66 ppm (carbon atoms from 4-oxo-1,4-dihydroquinoline-3-carboxylic acid), 140.33 and 61.83 ppm (carbon atoms from COCH_3_), 106.66 ppm (carbon atoms at 4-oxo-1,4-dihydroquinoline-3-carboxylic acid), 54.46, 51.87, 41.32, 34.51, 20.51 and 17.48 ppm (carbon atoms from (4aS,7aS)-octahydro-6H-pyrrolo [3,4-b]pyridin-6-yl)), and 40.52, 9.57 and 8.36 ppm (CH and CH_2_ groups from cyclopropyl, respectively).

Lithium salts did not show any proton bands and displayed characteristic carbon bands around 122.18–107.77 ppm for [Li][BETI] and 124.57–114.98 ppm for [Li][NTF_2_] [46]. GUMBOS with [TPB] anion showed characteristic proton bands at 7.18, 6.97 and 6.84 ppm and characteristics carbon bands between 164.18–162.63 ppm (for the four outer carbon atoms), 135.62, 128.99–125.34 and 121.60 ppm (for the twenty inner carbon atoms). Sodium docusate GUMBOS presented proton bands [47] at 4.05, 3.93–3.73, 2.90, 2.78, 1.49, 1.41–0.98 and 0.93–0.78 ppm and carbon bands at 171.01, 165.79, 66.16–66.03, 61.42, 38.16, 34.09, 28.59, 28.31, 23.15, 22.36, 13.89 and 10.79 ppm. Sodium deoxycholate GUMBOS showed characteristic proton bands [48] at 4.45 ppm, 4.20, 3.78, 3.24, 2.21–2.08, 1.84–0.96, 0.91, 0.84 and 0.59 ppm and carbon bands at 176.34, 70.98, 69.92, 47.44, 46.18, 45.98, 41.60, 36.29, 33.80, 32.90, 30.96–30.22, 28.58, 27.17, 26.97, 26.08, 23.49, 23.08, 16.91 and 12.43 ppm.

#### 2.2.2. FT-IR Spectroscopy

**Figure 3:**
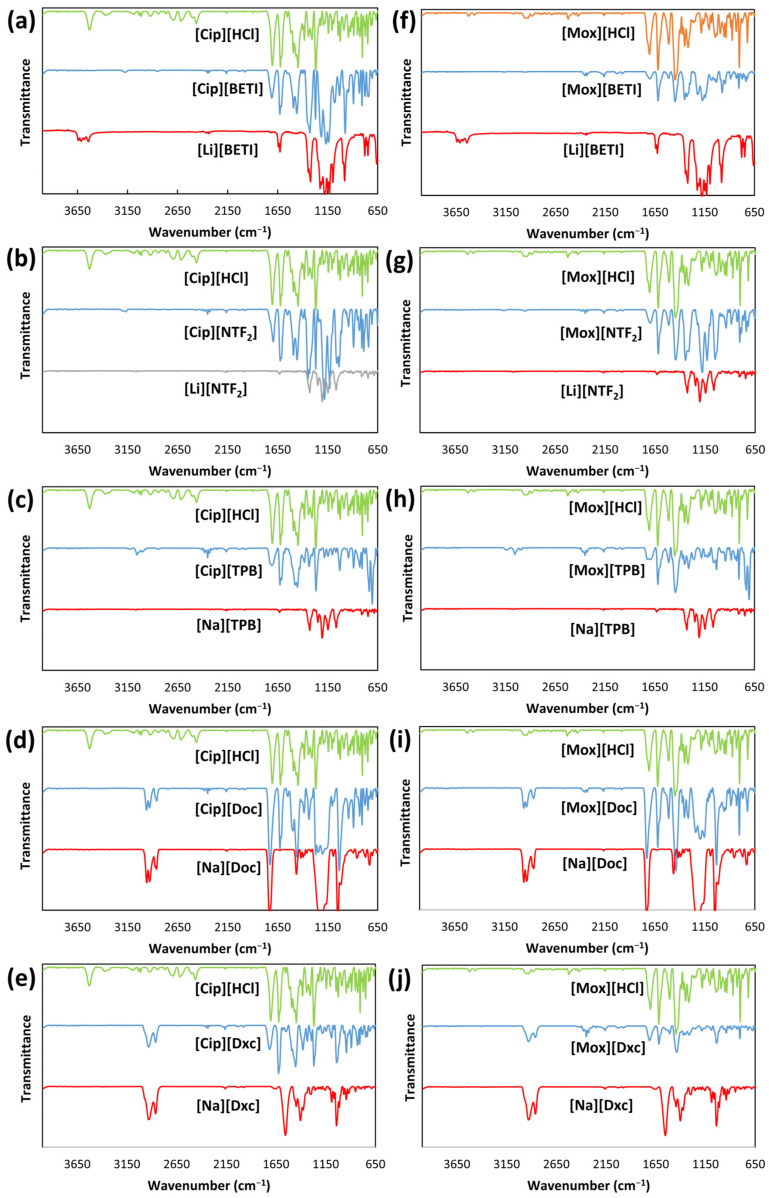
Overlay of FT-IR spectra of parent compounds (green and red) and the synthesized GUMBOS (blue): (**a**) [Cip][BETI], (**b**) [Cip][NTF_2_], (**c**) [Cip][TPB], (**d**) [Cip][Doc], (**e**) [Cip][Dxc], (**f**) [Mox][BETI], (**g**) [Mox][NTF_2_], (**h**) [Mox][TPB], (**i**) [Mox][Doc] and (**j**) [Mox][Dxc].

FT-IR spectra (Figure 3) of parent compounds, [Cip][HCl] and [Mox][HCl], allowed detection and confirmation of functional groups and covalent bondings.

Peaks at 3530 cm^−1^ and 3526 cm^−1^ represent the stretch vibration of O-H and peaks at 3370 cm^−1^ and 3437 cm^−1^ were allocated to stretching by N-H. The bands at 1703 cm^−1^ and 1707 cm^−1^ indicated carbonyl C=O stretching, whereas the peaks at 1622 cm^−1^ and 1620 cm^−1^ were assigned to C=C stretching of quinolones. The bands at 1447 cm^−1^ represented C–O and the peaks at 1269 cm^−1^ and 1262 cm^−1^ represent O-H bending which confirmed the existence of the carboxylic acid. Additionally, noticeable absorption peaks at 1024 cm^−1^ and 1026 cm^−1^ were assigned to C-F group [49,50].

For imide-based lithium salts [51], peaks were found at 1321 cm^−1^ and 1329 cm^−1^ (S=O stretching), 1181 cm^−1^ and 1206 cm^−1^ (CF_3_), 779 cm^−1^ and 800 cm^−1^ (C–S and S–N bondings), 750 cm^−1^ and 747 cm^−1^ (S–N stretching) for [Li][BETI] and [Li][NTF_2_], respectively.

For sodium docusate [52], characteristic bands were observed at 2959 cm^−1^, 2928 cm^−1^, and 2861 cm^−1^ allocated to the stretching vibration of C-H. Other characteristic peaks were seen and assigned at 1732 cm^−1^ (C=O ester stretching), 1462 cm^−1^ (C-H bending), 1209 cm^−1^ (S=O stretching), and 1019 cm^−1^ (for CCO bonds). For sodium deoxycholate [53], peaks at 2932 and 2863 cm^−1^ are allocated to the stretching C–H bond. Furthermore, an intense peak at 1557 cm^−1^ is assigned to C=O bond of the carboxylate ester group and another peak at 1042 cm^−1^ is allocated to CCO bonds.

For all GUMBOS, the disappearance of peaks representing the hydroxyl and amine groups of the parent compounds [Cip][HCl] and [Mox][HCl], proved protonation and interaction of the N-H of the cation with the anion. Some shifts and merges of characteristic vibration bands of both moieties also confirmed this. In [Cip][BETI] and [Cip][NTF_2_], an observable merge is present with addition of peaks for S=O and C-F bonds to the peaks of [Cip] spectra. For [Cip][Doc] and [Cip][Dxc], a C=O shift was observed for GUMBOS with C-H, S=O, and CCO peaks of the anions. The same pattern changes were observable for moxifloxacin GUMBOS.

#### 2.2.3. ESI-MS

Analysis of ESI-MS results complemented previous results for synthesis of FQ-based GUMBOS. Theoretical *m*/*z* values were calculated with ChemDraw Professional. In the positive ion mode spectra, peaks with *m*/*z* values of 332.1410 and 402.1829 were expected to [Cip] and [Mox] cations. For negative ion mode spectra, peaks were expected with *m*/*z* values of 379.9109, 279.9178, 319.1664, 421.2260, 391.2853 and were assigned to [BETI], [NTF_2_], [TPB], [Doc] and [Dxc] anions. The presence of the cations and anions was verified in all synthesized GUMBOS. Additionally, experimental values obtained were in accordance with expected *m*/*z* values (Table 2).

### 2.3. Thermal Stability Evaluation

Onset temperatures and peak temperatures were determined for all precursor compounds (Table 3) and GUMBOS (Table 4). DSC thermograms can be found in the Supplementary Materials file.

Thermal analysis of parent compounds was similar to previous studies that used [Cip][HCl] [54], [Mox][HCl] [55], [Li][BETI] [56], [Li][NTF_2_] [57], [Na][TPB] [58], [Na][Doc] [47] and [Na][Dxc] [59].

According to the results obtained, it was observed that [Cip][TPB] and [Mox][TPB] had the lowest melting point of all the synthesized GUMBOS. DSC results of some GUMBOS display at least one wide endothermic peak below 100 °C, which may be linked to dehydration of adsorbed water or their amorphous characteristics [12,60]. Melting points of GUMBOS decreased when compared with the melting points of the relatively large parent cations, 325.7 °C for [Cip][HCl] and 257.5 °C for [Mox][HCl]. DSC thermogram of [Cip][HCl] shows dehydration at around 149.1 °C and melting at 325 °C. Deviation in melting points of synthesized GUMBOS can be elucidated possibly by the size and symmetry differences of cations [61]. Furthermore, the lower melting points of [Cip][TPB] and [Mox][TPB], in comparison to all the other GUMBOS analyzed, can be explained by the much lower melting point of [TPB].

Thermogravimetric analysis (TGA) spectra were obtained for all GUMBOS. TGA curves can be found in the Supplementary Materials file. TGA is performed to study the thermal stability of a sample and its volatile components by continuous assessment of the weight variation that happens when a sample is heated at a constant rate. Table 5 presents key characterization parameters of GUMBOS’ TGA curves obtained in N_2_ atmosphere.

Overall, onset temperatures of GUMBOS decreased when compared to the parent [Cip][HCl], except for [Cip][Dxc] and increased compared to parent [Mox][HCl], except for [Mox][TPB]. Overall, GUMBOS with [TPB] anion decomposed at temperatures much lower than other GUMBOS. At low temperatures, [TPB] can undergo decomposition reactions, although reaction pathways and rates may depend on conditions and presence of other substances [63]. Possible decomposition pathways involve loss of a phenyl group from the boron atom, resulting in triphenylborane which is stable at low temperatures or formation of phenyl radicals which can react with each other and lead to formation of biphenyl and other by-products [64].

### 2.4. Octanol–Water Partition Coefficients

The relative hydrophobicities of GUMBOS were evaluated through the determination of log K_O/W_ values for all synthesized compounds (Table 6). Data assessment indicates that among [Cip]-based GUMBOS, [Cip][Dxc] was the most hydrophilic and [Cip][Doc] was the most hydrophobic. Among [Mox]-based GUMBOS, [Mox][Dxc] was the most hydrophobic compound.

Lithium salts, such as [Li][BETI] and [Li][NTF_2_], are generally considered hydrophilic as they readily dissociate in aqueous solutions, meaning they break apart into lithium ions (Li^+^) and other ions and interact with water molecules. GUMBOS derived from these precursors were also mostly hydrophilic as observed for [Cip][BETI], [Mox][BETI] and [Mox][NTF_2_]. The other synthesized GUMBOS were obtained through different sodium salts. Sodium tetraphenylborate is frequently used as a precipitation agent and is composed of a hydrophilic sodium ion (Na^+^) and a hydrophobic tetraphenylborate ion (BPh_4_^−^) which is composed of four phenyl rings bonded to a negatively charged boron atom [66].

The large size of the surrounding phenyl rings in [Na][TPB] causes significant distortion of hydrogen bonding and disruption of the hydration shell. As a result, the hydrophobic anions have a preference for dissolution in organic based solvents, such as oil, while the cations tend to remain in water. This leads to an antagonistic behavior between the cations and anions, causing them to distribute unevenly when added to a mixture of water and an organic solvent [67]. Unlike smaller counterions, such as nitrate and halides, [TPB] confers lipophilicity to its salts. Sodium docusate and sodium deoxycholate are anionic surfactants, meaning that it has both hydrophilic and hydrophobic regions in their molecular structures [68]. GUMBOS obtained with these sodium salts were mainly hydrophobic possibility due to the anion precursor characteristics, with the exception of [Cip][TPB] and [Cip]Dxc]. The different results obtained for these two individual compounds could be due to specific interactions with the cation structure that result in the more hydrophilic profile.

Some antimicrobial agents, such as phosphonium and quaternary ammonium compounds, are known to be more effective against bacteria when paired with more hydrophobic anions. This is because hydrophobic anions can enhance interactions between positively charged agents and the negatively charged bacterial cell membrane, leading to increased disruption of the membrane and improved antimicrobial activity. One study has reported influence of anion hydrophobicity on the antibacterial performance of ILs with small hydrophilic anions, such as [Cl], remaining on the external membrane surface while the more hydrophobic anions such as [NTF_2_], penetrated the membrane and introduced the hydrocarbon tail into the bacterial membrane [69], thus having the potential to increase its antimicrobial activity.

In this study, GUMBOS displayed variable degrees of hydrophobicity that were credited to anion exchange. Rhodamine-based GUMBOS also showed different hydrophobicities trends with ester and carboxylic acid functional groups on the rhodamine cation, with the latter showing an anion hydrophobicity order of [X][TPB] > [X][BETI] [70]. Hydrophobicity results of our compounds with carboxylic acid functional groups also followed the same order for [Mox]-based GUMBOS while the [Cip]-based GUMBOS hydrophobicity values were similar.

### 2.5. Antibacterial Susceptibility Testing

The antibacterial profiles of synthesized GUMBOS were evaluated on Gram-positive *S. aureus* and Gram-negative *E. coli* bacteria strains. The current epidemiologic cutoff values (ECOFF) for [Cip] and [Mox], which is the minimum inhibitory concentration (MIC) distribution of a drug that separates wild type bacterial populations from populations with acquired or mutational resistance to the drug, are 0.06 mg·L^−1^ and 0.25 mg·L^−1^ for *E. coli* and 2 mg·L^−1^ and 0.25 mg·L^−1^ for *S. aureus* [71]. The concentrations evaluated in this work were 0.25 mg·L^−1^ and 25 mg·L^−1^. One study using the same *E. coli* O157:H7 strain employed in this study observed inhibition of growth by a chlorhexidine di-ampicillin at concentrations between 0.06–0.12 mg·L^−1^ [13].

**Figure 4:**
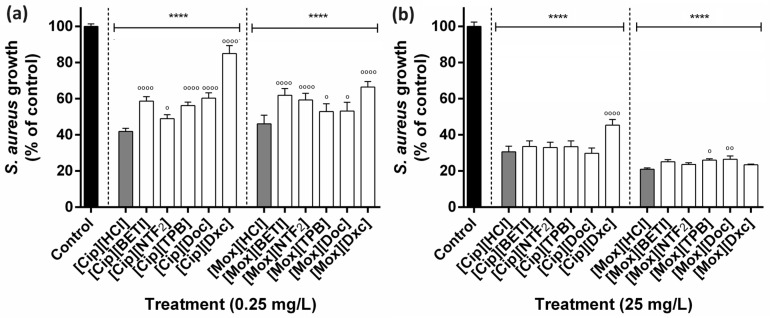
Antibacterial performance of ciprofloxacin- and moxifloxacin-based GUMBOS at (**a**) 0.25 mg·L^−1^ and (**b**) 25 mg·L^−1^ against Gram-positive *S. aureus*. Bars indicate the mean percentage bacterial growth values and the error bars indicate the standard error of the mean (*n* = 3). Black bars represent negative control, grey bars represent cation parents and white bars represent GUMBOS. Statistically significant differences between negative control (denoted by *) at: **** *p* < 0.0001 and the cation parent (denoted by ^o^) at: ^oooo^ *p* < 0.0001, ^oo^ *p* < 0.01, ^o^ *p* < 0.05.

Data analysis showed that GUMBOS at the lower concentration of 0.25 mg·L^−1^ already inhibited Gram-positive *S. aureus* growth after 24 h of exposure for all samples in comparison with the negative control and [Cip][NTF_2_], [Mox][TPB] and [Mox][Doc] were the GUMBOS with activities closer to their precursor (Figure 4a). Increasing the concentration to 25 mg·L^−1^ improved the antibacterial activity for all GUMBOS with no significant differences from their corresponding precursors (Figure 4b), except for [Cip][Dxc], [Mox][TPB] and [Mox][Doc]. It was observed that [Cip][Dxc] was the least antimicrobial agent of all tested samples both for higher and lower concentrations.

**Figure 5:**
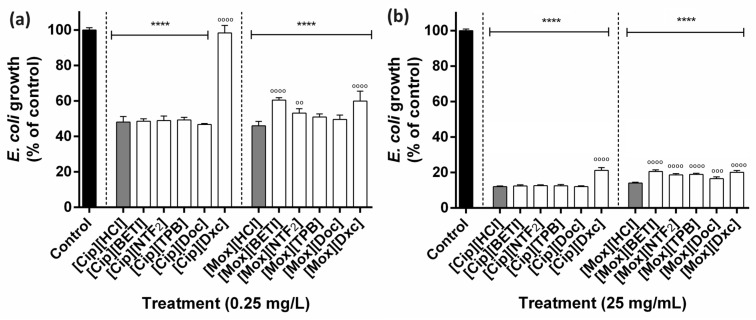
Antibacterial performance of ciprofloxacin- and moxifloxacin-based GUMBOS at (**a**) 0.25 mg·L^−1^ and (**b**) 25 mg·L^−1^ against Gram-negative *E. coli* O157:H7. Bars indicate the mean percentage bacterial growth values and the error bars indicate the standard error of the mean (*n* = 3). Black bars represent negative control, grey bars represent cation parents and white bars represent GUMBOS. Statistically significant differences between negative control (denoted by *) at: **** *p* < 0.0001 and the cation parent (denoted by o) at: ^oooo^ *p* < 0.0001, ^ooo^ *p* < 0.001, ^oo^ *p* < 0.01.

Against Gram-negative *E. coli*, [Cip]-based GUMBOS performance were similar to the precursor [Cip][HCl] for almost all GUMBOS at the lowest (Figure 5a) and the highest (Figure 5b) concentrations, except for [Cip][Dxc]. This confirms that in this circumstance, physicochemical changes in [Cip] did not affect antibacterial activity. When evaluating [Mox]-based GUMBOS, the ones that presented similar performance as the control [Mox][HCl] were [Mox][TPB] and [Mox][Doc] at the lowest concentration, while all others showed significant differences. Overall, [Cip]-based GUMBOS showed similar performances to their precursor, [Cip][HCl]. Conjugates with the deoxycholate anion produced the least effective antibacterial compound among those evaluated. This might be attributed to the fact that Gram-negative bacteria such as *E. coli* and *Salmonella* have demonstrated significant resistance to [Na][Dxc] through several mechanisms, including the use of different active efflux pumps, down-regulation of outer membrane porins and triggering multiple stress responses [72].

GUMBOS synthesized in this study were highly effective against Gram-negative *E. coli* than Gram-positive *S. aureus*. In the literature, most antibacterial agents are more efficient against Gram-positive than Gram-negative bacteria. Gram-positive bacteria possess a cytoplasmic membrane coated with a thick layer of peptidoglycan, lacking lipopolysaccharides. In contrast, Gram-negative bacteria feature an additional hydrophobic membrane consisting of lipopolysaccharides, phospholipids, and lipoproteins. This additional membrane can pose a formidable barrier that may impede penetration of antibacterial agents, acting as a protective shield. The presence of this outer membrane barrier, along with active efflux mechanisms, is likely to influence permeation of various compound classes to varying extents [73]. This discrimination of activities has also been verified in other studies with halogenobenzene piperidinium and pyrrolidinium hybrids [74], triphenylamine phosphonium ILs [75], imidazolium and pyridinium ILs [76], methylimidazolium–furanchalcone hybrids [77], imidazolium and piperidinium ILs [78] and pyrithione ILs [79]. In this work using fluoroquinolones as a different class of compounds, GUMBOS showed good performance against Gram-negative *E. coli*. A study using ciprofloxacin and norfloxacin as anions in six organic salts also showed higher broad-spectrum antibacterial performance against Gram-negative *K. pneumoniae* than the Gram-positive *S. aureus* and *B. subtilis* [22]. Fluoroquinolones conjugated with fatty acids revealed the highest bactericidal potential against four standard bacterial strains with sorbic and geranic acids for ciprofloxacin derivatives and acetic, unsaturated crotonic and sorbic acids for moxifloxacin derivatives [40]. β-lactam-based chlorhexidine GUMBOS caused fractional inhibitory concentration interaction index values for *E. coli* lower than those for *S. aureus* [14].

It was demonstrated that using fluoroquinolones, one class of antimicrobials, as parent compounds, different novel GUMBOS can be produced with promising properties that can help in the fight against antimicrobial resistance.

### 2.6. Cytotoxicity Evaluation

Cytotoxicities of prepared ciprofloxacin and moxifloxacin-based GUMBOS and corresponding starting compounds towards L929 mouse fibroblasts were determined using the resazurin reduction assay method. Besides the concentrations tested in the antibacterial susceptibility test (0.25 and 25 mg·L^−1^), one concentration above and below that range were also evaluated (0.025 and 50 mg·L^−1^). By testing both higher and lower concentrations, cytotoxicity assays provide a comprehensive understanding of the substance’s safety profile and therapeutic potential, which enables to make informed decisions regarding its use.

**Figure 6:**
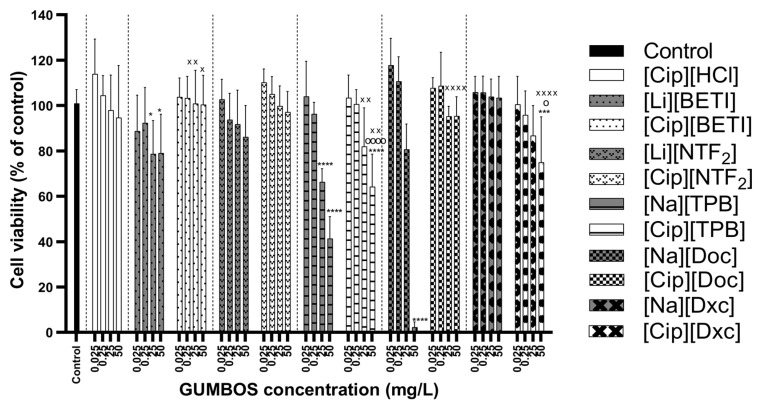
Cell viability results with resazurin assay on L929 cells after 24 h exposure to ciprofloxacin-based GUMBOS and its corresponding parent materials. Data are represented as mean ± standard deviation (*n* = 9). Statistically significant differences between negative control (denoted by *) at: **** *p* < 0.0001, *** *p* < 0.001, * *p* < 0.05; the cation parent [Cip][HCl] (denoted by o) at: ^oooo^ *p* < 0.0001, ^o^ *p* < 0.05; the respective anion (denoted by ^×^) at: ^××××^ *p* < 0.0001, ^××^ *p* < 0.01, ^×^ *p* < 0.05.

[Cip]-based GUMBOS were considered to be non-toxic to L929 cells at the lowest concentrations of 0.025–0.25 mg·L^−1^, with viability percentages similar to the negative control (Figure 6). At the highest concentration of 50 mg·L^−1^, that showed some levels of cell toxicity compared to negative control were [Cip][TPB] and [Cip][Dxc]. In the case of [Cip][TPB], its toxicity may be attributable to the parent material [Na][TPB] which was observed to be cytotoxic at the two highest concentrations. As for [Cip][Dxc], it might be result of interaction between cation and anion since both precursors presented higher viability profiles.

[Na][Doc] was highly cytotoxic, causing total cell death at 50 mg·L^−1^. Studies have found that cytotoxicity against Vero cells of [Na][Doc] is time- and dose-dependent, being cytotoxic after short-term exposure (1 h) and highly cytotoxic after long-term exposure (72 h) at 0.01% concentrations [80]. Cytotoxicity of docusate alone has also been verified with neuroblastoma cells, hepatocellular carcinoma (HepG2) and triple-negative breast cancer (MDA-MB-231) cells [81]. In this study, despite some counterions being highly cytotoxic, GUMBOS showed an improvement of cell viability overall. This was the case of GUMBOS with [Na][Doc] anion, where [Cip][Doc] had much lower cytotoxicity levels and revealed a similar performance as the negative control for all concentrations.

**Figure 7:**
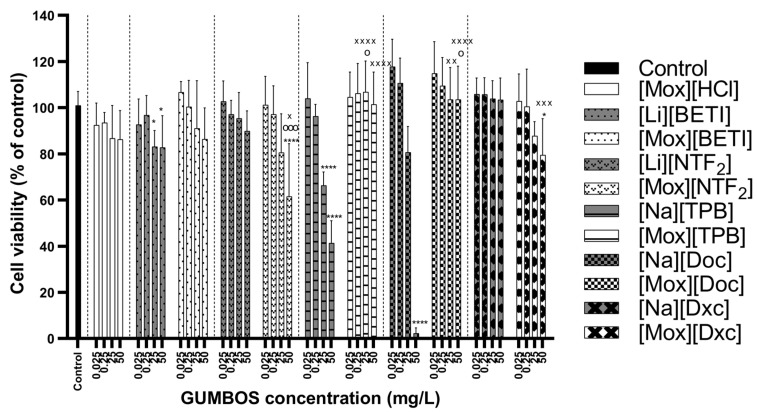
Cell viability results with resazurin assay on L929 cells after 24 h exposure to moxifloxacin-based GUMBOS and its corresponding parent materials. Data are represented as mean ± standard deviation (*n* = 9). Statistically significant differences between negative control (denoted by *) at: **** *p* < 0.0001, * *p* < 0.05; the cation parent [Mox][HCl] (denoted by ^o^) at: ^ooo^ *p* < 0.001, ^o^ *p* < 0.05; the respective anion (denoted by ^×^) at: ^××××^ *p* < 0.0001, ^×××^ *p* < 0.001, ^××^ *p* < 0.01, ^×^ *p* < 0.05.

[Mox]-based GUMBOS were observed to be non-toxic at the lowest concentrations, with performances similar to the negative control (Figure 7). Behavior patterns of [Mox]-based GUMBOS were also similar to [Cip]-based GUMBOS obtained previously where certain anions were proven cytotoxic to L929 cells, such as [Li][BETI], [NaTPB] and [Na][Doc] mainly at the two highest concentrations, whose values improved when combined with moxifloxacin. Exceptions were [Mox][NTF_2_] and [Cip][Dxc] which showed slight toxicity at 50 mg·L^−1^, despite the anion not causing toxicity to cells.

Other studies have also obtained non-toxicity results for healthy cells with other FQ-based compounds. Cell viability of the intestinal epithelial Caco-2 cells was preserved when FQ-based salts coupled with esters were incubated for hours, revealing no cytotoxic effect over the concentration range tested [82]. Ciprofloxacin and norfloxacin coupled with ILs showed no cell viability reduction also on mouse fibroblasts (3T3 cell line) at concentrations of 10 μM, corresponding to 0.003313 mg·L^−1^ in ciprofloxacin and 0.003193 mg·L^−1^ in norfloxacin [22]. Thus, at higher concentrations, our compounds still maintained cell viability, which contributes to the notion that these compounds are potentially useful for further testing at the evaluated concentrations.

On the other hand, novel conjugates of ciprofloxacin and moxifloxacin with fatty acids exhibited a pronounced cytotoxic potential against prostate cancer cells than the parent drug [40], which could be an additional advantage of these alternative compounds to be explored.

### 2.7. Hemolysis Assay

Following administration, drugs encounter a range of biological obstacles that can impede their desired therapeutic effects. Among these barriers, blood acts as a hindrance since the substances within the drugs can engage with biomolecules, potentially inducing alterations in structures. Consequently, such changes can cause modifications in biological responses. These interactions are inevitable and pose a potential risk, making it crucial to evaluate hemolytic activity. To ascertain the absence of severe pharmacologically mediated toxicity, an in vitro study was conducted, adhering to the guidelines established by the European Medicines Agency (EMA) [83]. This study aimed to assess the extent of red blood cell lysis and subsequent release of hemoglobin into the plasma following exposure to synthesized GUMBOS. In a concentration range of 0.025 to 50 mg·L^−1^, potential toxicity was evaluated after 1 h, through percentage of hemolysis. Ciprofloxacin and moxifloxacin are not typically known to cause hemolysis at therapeutic doses. However, it causes hemolysis in a dose-dependent manner and in rare cases, high concentrations can potentially lead to hemolytic anemia [84].

**Figure 8:**
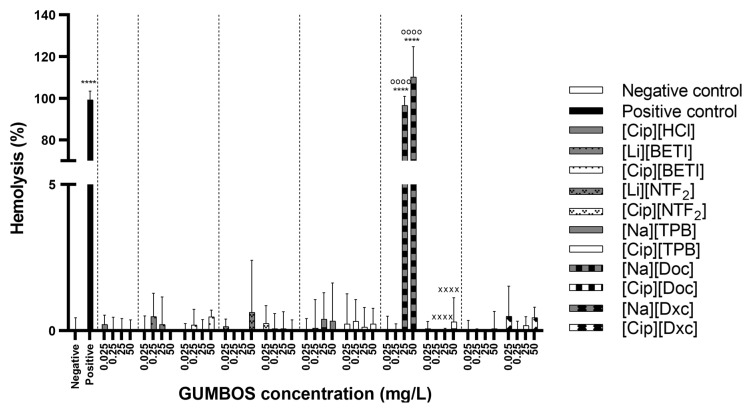
Hemolysis results after 24 h exposure to ciprofloxacin-based GUMBOS and its correspondent parent materials. Data represent the average of three assays using RBCs from three human donors. Statistically significant differences between negative control (denoted by *) at: **** *p* < 0.0001; the cation parent [Cip][HCl] (denoted by ^o^) at: ^oooo^ *p* < 0.0001; the respective anion (denoted by ^×^) at: ^××××^ *p* < 0.0001.

**Figure 9:**
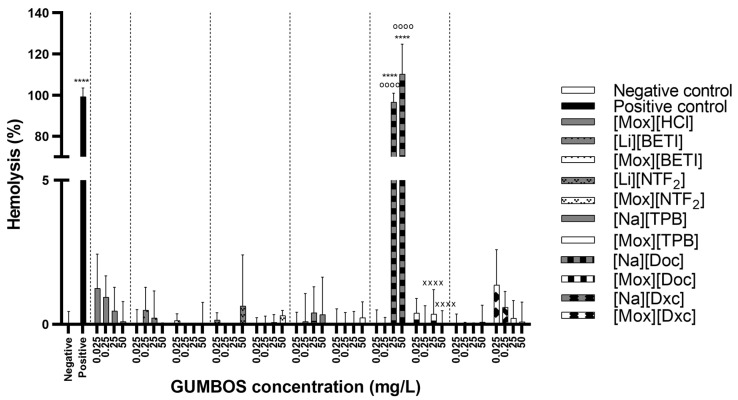
Hemolysis results after 24 h exposure to moxifloxacin-based GUMBOS and its correspondent parent materials. Data represent the average of three assays using RBCs from three human donors. Statistically significant differences between negative control (denoted by *) at: **** *p* < 0.0001; the cation parent [Mox][HCl] (denoted by ^o^) at: ^oooo^ *p* < 0.0001; the respective anion (denoted by ^×^) at: ^××××^ *p* < 0.0001.

For [Cip]-based (Figure 8) and [Mox]-based (Figure 9) GUMBOS, all novel compounds revealed low values or null hemolytic activity. As for the parent compounds, almost all revealed no hemolytic activity with the exception of [Na][Doc] at the two highest concentrations (25 and 50 mg·L^−1^). As is true for most anionic surfactants, the use of higher doses of sodium docusate can lead to hemolysis in susceptible patients likely because it can generate reactive oxygen species (ROS) that can damage red blood cells in susceptible individuals [85]. In particular, those with glucose-6-phosphate dehydrogenase (G6PD) deficiency are damaged, as the lack of G6PD can easily lead to cell break down when the person is exposed to certain triggers [86]. Fluoroquinolone conjugates with a cell-penetrating peptide verified the non-hemolytic activity of FQ and hemolytic activity for conjugates in the 10–100 µM range together with mammalian cytotoxicity due to the intrinsic cytoplasmic membrane disruption activity of the peptide, despite the selectivity index values of the conjugates for bacteria and yeasts being favourable [41]. Hemolytic studies have also been performed with ionic liquids based on active pharmaceutical ingredients. One study with ibuprofen-based cholinium and imidazolium ILs revealed hemocompatibility even at concentrations higher than that of the ibuprofen, the most commonly available over-the-counter NSAIDs [87], while other study with fluorinated ILs at lower concentrations showed hemolysis [88].

The hemocompatibility results are in agreement with cytotoxicity evaluation, where sodium docusate, which was the most hemolytic, was also one of the most cytotoxic compounds at the highest evaluated concentration. In general, hemolysis serves as the commonly utilized initial evaluation for toxicity in drug development as it can be linked to cytotoxicity assays. This is because the primary cause of toxicity often involves disruption of cell membranes [89].

Evaluation of fluoroquinolone-based GUMBOS prepared in this study demonstrated that, by a simple and effective modification of standard FQ drugs, it is possible to tailor certain properties of the drug to improve overall performance. The ability to tune their antimicrobial spectrum makes these FQ-based compounds a very promising tool with potential to create novel effective formulations. The data from this study will certainly increase value of GUMBOS as an easy and affordable path to improve physicochemical properties of active pharmaceutical ingredients without risking their bioactivity or, possibly, even improving their antibacterial activity.

## 3. Materials and Methods

### 3.1. Materials and Reagents

Ciprofloxacin hydrochloride monohydrate and moxifloxacin hydrochloride monohydrate were acquired from Sigma-Aldrich^®^ (St. Louis, MO, USA). Lithium bis(perfluoroethylsulfonyl)imide ([Li][BETI]) was purchased from TCI Chemicals (Portland, OR, USA), and lithium bis(trifluoromethanesulfonyl)imide ([Li][NTF_2_]), sodium tetraphenylborate ([Na][TPB]), sodium docusate ([Na][Doc]) and sodium deoxycholate ([Na][Dxc]) were all obtained from Sigma-Aldrich^®^ (St. Louis, MO, USA). 1-octanol was acquired from Honeywell (Charlotte, NC, USA). Dimethyl sulfoxide (DMSO), deuterated dimethyl sulfoxide (DMSO-d6), Dulbecco’s phosphate-buffered saline (PBS), Resazurin sodium salt, Triton^TM^ X-100, Trypan blue solution, Tryptic Soy Broth (TSB) and Tween^®^ 80 were all obtained from Sigma-Aldrich^®^ (St. Louis, MO, USA). Dulbecco’s Modified Eagle’s Medium (DMEM), 0.25% Trypsin-ethylenediamine tetraacetic acid (EDTA) (1X), Penicillin-Streptomycin (PenStrep) and Heat Inactivated Fetal Bovine Serum (FBS) were acquired from Gibco (Thermo Fisher Scientific™, Paisley, UK). L929 cell line (passages 13–17) was purchased from Cell Lines Service (CLS, Eppelheim, Germany). All prepared solutions were obtained with the use of ultrapure water purified by a specialized water system (Milli-Q^®^, 18.2 MΩ·cm, Healforce, Shanghai, China).

### 3.2. Synthesis of GUMBOS

Ciprofloxacin and moxifloxacin-based GUMBOS were produced through methods performed in previous works [14] with minor modifications. GUMBOS were synthesized via anion metathesis reactions, where their hydrochloride anions were exchanged using five bulky organic anions from two inorganic lithium salts ([Li][BETI] and [Li][NTF_2_], one organic salt ([Na][TPB]) and two ionic surfactants ([Na][Doc] and [Na][Dxc]) at a molar ratio of 1:1.

Both the cation and anion were dissolved using deionized water, except for docusate and deoxycholate anions which were dissolved in methanol, and then stirred for 48 h in the dark at ambient temperature for yield optimization. Eventually, the precipitated GUMBOS were washed during three cycles with cold, deionized water to remove any by-products. Samples were frozen at −70 °C for 48 h in an ultra-low temperature freezer and subsequently freeze-dried using lyophilization overnight in a LyoQuest (Telstar, Barcelona, Spain). Finally, yields for each of the synthesized GUMBOS were calculated.

### 3.3. Structural Characterization

The structures of precursors and synthesized GUMBOS were evaluated using proton and carbon-13 nuclear magnetic resonance (NMR) and Fourier transform infrared (FT-IR) spectroscopy. Electrospray ionization mass spectrometry (ESI-MS) was also performed to corroborate the formation of synthesized GUMBOS.

#### 3.3.1. ^1^H- and ^13^C-NMR

Proton (^1^H, 400.15 MHz) and carbon-13 (^13^C, 100.62 MHz) NMR spectra were obtained with a Bruker Avance III HD NanoBay 400. Samples were prepared by dissolving GUMBOS in DMSO-d_6_. Chemical shifts (δ) were described in parts per million (ppm) and coupling constants (J) in hertz (Hz). Multiplicities were labeled with the following abbreviations: singlet (s), doublet (d), triplet (t), and multiplet (m). Residual signals from the solvents were used as an internal reference, using the following reference chemical shifts: DMSO-d_6_ at 2.50 ppm for ^1^H NMR and 39.52 ppm for ^13^C NMR [90].

#### 3.3.2. FT-IR Spectroscopy

FT-IR spectroscopy was executed to analyze the chemical structure of GUMBOS. Measurements were performed using a Bruker Tensor 27 FT-IR spectrometer (Bruker Scientific, Billerica, MA, USA), coupled with a Pike MIRacleTM Single Reflection ATR cell (Pike Technologies, Madison, WI, USA). Spectra were collected under transmission wavenumbers ranging from 4000 to 600 cm^−1^ with a spectral resolution of 4 cm^−1^ and 32 successive scans being run and averaged into one spectrum. For data gathering and initial processing of results, the software Spectrum version 10.03.09 (PerkinElmer, Waltham, MA, USA) was used.

#### 3.3.3. ESI-MS

Electrospray ionization mass spectrometry (ESI-MS) was used to determine molecular weights for samples. Samples were ionized via a “soft” ionization into small droplets that are further desolvated and enter the mass analyzer and subsequently the detector to determine the mass/charge ratio with increased sensitivity. ESI-MS was performed using an Agilent 6230 B-TOF LC/MS in positive and negative mode at the Mass Spectrometry Facility of Louisiana State University.

### 3.4. Thermal Stability Evaluation

Differential scanning calorimetry (DSC) analysis was performed for all GUMBOS and thermograms were obtained using a DSC 200 F3 Maia^®^ thermogravimetric analyzer (Netzsch, Selb, Germany). Small amounts of GUMBOS weighing about 0.5–5 mg were placed in an aluminum pan and heated from room temperature to 350 °C at a slow heating rate of 10 °C min^−1^ underneath a nitrogen purge (50 mL min^−1^). Afterwards, a cooling cycle was performed back to 25 °C at the same temperature rate. An empty pan was used as a reference during the DSC analysis.

Thermogravimetric analysis (TGA) was executed using a Hi-Res modulated TGA 2950 thermogravimetric analyzer (TA Instruments, New Castle, DE, USA) and curves were obtained for all GUMBOS. Approximately 1–3 mg of GUMBOS was placed in a platinum pan and heated from 25 °C to 575 °C at a slow heating rate of 10 °C min^−1^.

Some characteristic parameters were extrapolated. For DSC and TGA, onset temperatures (T_onset_) were determined as the temperature value matching the intersection point of the extrapolated initial baseline and the tangent or line through the linear section of the leading edge of the T_peak_ point where the T_peak_ was the temperature value at the highest degradation rate. For TGA, additional data were collected such as the temperature value where weight loss starts (T_start_), and the percentage of mass residue remaining at 500 °C which allows evaluation of the short-term stability. All these parameters were extrapolated using the software NETZSCH Proteus^®^ version 6.1081 (Netzsch-Gerätebau GmbH, Selb, Germany) for DSC analysis and Universal Analysis 2000 version 4.5A (TA Instruments-Waters LLC, New Castle, DE, USA) for TGA analysis.

### 3.5. Octanol–Water Partition Coefficients

Following synthesis and structural characterization of FQ-based GUMBOS, octanol/water partition coefficient (K_o/w_) values were obtained through a shake-flask method with minor modifications [91]. Initially, water and 1-octanol were saturated by agitation for 24 h at room temperature. GUMBOS were solubilized in 5 mL of water-saturated octanol (C_i_), to which an equivalent volume of octanol-saturated water was added. This solution subjected to 24 h of agitation and the absorbance of the upper layer was measured at a wavelength of 280 nm. Sample concentration in octanol (C_o_) was determined through a calibration curve, which was created using six solutions of different concentrations (4–30 μmol·L^−1^) of GUMBOS in 1-octanol. Later, GUMBOS concentration in water (C_w_) were obtained by the following equation C_i_ − C_0_ = C_w_. Octanol/water partition coefficient (K_o/w_) values were then calculated using K_o/w_ = C_o_/C_w_. Data were representative of three replicates executed for each of the GUMBOS.

### 3.6. Antibacterial Susceptibility Testing

To evaluate the antimicrobial profile, a study was performed using the broth microdilution standard method [92] with minor modifications using DMSO as solvent. Standard laboratory strains of Gram-positive *Staphylococcus aureus* subsp. *aureus Rosenbach* ATCC 25923 and Gram-negative *Escherichia coli* O157:H7 were studied. TSB was used as the culture medium for bacteria and samples with bacteria in media were considered positive controls.

Antibacterial activity of GUMBOS was evaluated by comparing it with negative controls, as well as with the antibacterial activity of the parent compounds, [Cip][HCl] and [Mox][HCl]. All compounds were tested at concentrations in the range of 0.25–25 mg·L^−1^ as literature shows that for most strains of *S. aureus*, MICs of ciprofloxacin are between 0.025–5 mg·L^−1^ [93,94] and moxifloxacin MICs can range from 0.032–2 mg·L^−1^ [95]. These values are determined through laboratory testing and can vary depending on the testing methodology and conditions, thus the range increase in this study to 25 mg·L^−1^. Stock solutions were prepared using water for water-soluble compounds while non-water soluble compounds were reconstituted using a deionized water solution containing 5% DMSO [96]. Following that, the compounds were diluted using fresh tryptic soy broth (TSB) to achieve desired concentrations. Initially, agar media was used to grow additional bacteria to correlate absorbance readings to microbial counts. Then, a solution of 0.1 mL of *S. aureus* added to 10 mL of TSB was incubated at 37 °C for 24 h and repeated for three more days until enough bacteria was obtained. After centrifugation and washing steps with PBS (1×) solution until obtaining a homogenized pellet, the inoculum was then diluted to 0.06 absorbance at 630 nm or 0.5 McFarland [97]. After compounds were diluted in TSB, 100 μL of samples with 100 μL of *S. aureus* inoculum were then transferred to a 96-well flat bottom microtitre plate (TPP, Switzerland) which was sealed and left in incubation at 37 °C for 24 h. Absorbance was read at 630 nm with a BioTek universal microplate reader (Agilent Technologies, VT, USA) to determine the turbidity in the wells [97,98]. Three wells were prepared per sample. To ensure the viability and growth of bacteria in the culture media, negative controls were conducted along with a positive culture using the media containing only the bacteria. [Cip][HCl] served as the reference antibacterial agent.

### 3.7. Cytotoxicity Evaluation

To assess the impact of GUMBOS on cell viability, the resazurin assay was employed using L929 mouse fibroblast cells as described previously by Page et al. [99]. The L929 cells were cultured at 37 °C in a 5% CO_2_ atmosphere, utilizing DMEM supplemented with 10% FBS and 1% streptomycin/gentamycin. Once the cell culture reached 70–80% confluence, it was detached using a 0.25% (*w*/*v*) trypsin-ethylenediaminetetraacetic acid (EDTA) solution. The cells were then subjected to centrifugation with the Heraeus Multifuge X1R centrifuge (Thermo Fisher Scientific; Waltham, MA, USA) and resuspended in fresh medium. Viable cell counts were obtained through a Neubauer chamber (Improved Neubauer Bright-Line, Boeco; Hamburg, Germany).

For experiments, L929 cells were seeded at a density of 5 × 10^4^ cells per well in 96-well microplates and supplemented with DMEM medium. Plates were placed in a humidified incubator at 37 °C with 5% CO_2_ for 24 h to allow cell attachment and growth. Subsequently, the culture medium was replaced with different concentrations of each sample being tested. After 24h incubation, the medium was replaced with fresh culture medium containing 10% resazurin. Cells were then incubated for 4 h in the dark at 37 °C with 5% CO_2_ to allow resazurin conversion. Fluorescence of resorufin was measured using a plate reader (Synergy HT plate reader-Biotek Instruments, Winooski, VT, USA) with excitation at 560 nm and emission at 590 nm. Negative control wells contained only culture medium, while positive control wells contained the cation precursor, the active pharmaceutical ingredient. All experiments were conducted in triplicate.

### 3.8. Hemolysis Assay

For the hemolysis assay, human blood was obtained from healthy donors and stored in tubes coated with EDTA. Blood samples were generously provided by the Hematology Department of Centro Hospitalar Universitário do Porto (Hospital de Santo António, Porto, Portugal). To separate red blood cells (RBCs) from other components, the samples were centrifuged at 955 × *g* for 5 min at 4 °C using an Allegra X-15R Centrifuge (Beckman Coulter, Fullerton, California). The supernatant was discarded, and the RBCs were washed three times with a sterile saline solution (0.85% *w*/*v*). The obtained pellet of RBCs was then diluted in a 4% (*v*/*v*) saline solution.

GUMBOS were diluted to the desired concentrations using saline solution. Positive controls (100% lysis) consisted of a solution of Triton-X-100 (1% *v*/*v*), while negative controls (0% lysis) were prepared using saline solution alone. In a 96-well microplate, 100 μL of RBCs were incubated with 100 μL of the samples at 37 °C for 1 h. Subsequently, the supernatant was discarded, and the absorbance of hemoglobin was measured at 415 nm using UV-Vis spectroscopy and a microplate reader (Synergy HT plate reader-Biotek Instruments, Winooski, VT, USA). The percentage of hemolysis was calculated as follows:

(1) $$Hemolysis\;\left(\%\right)=\frac{Abs\;\left(sample\right)-Abs\;\left(negative\;control\right)}{Abs\;\left(positive\;control\right)-Abs\;\left(negative\;control\right)}\times 100$$

### 3.9. Statistical Analysis

An analysis to assess the distribution of the collected data was conducted and the data did not conform to a normal distribution. The *p*-value obtained from the Shapiro–Wilk test was below the significance level (α) and the Q-Q plot displayed noticeable deviations, confirming the non-normal distribution of the data. Results of antibacterial testing of the treatment groups with GUMBOS and controls were analysed using non-parametric methods such as one-way analysis of variance (ANOVA) and Tukey’s multiple-comparisons test. Differences between groups and control were considered to be significant at a *p* value of <0.05 with a 95% confidence interval. Statistical analyses were performed with GraphPad Prism 6.0 (GraphPad Software, Inc., San Diego, CA, USA).

## 4. Conclusions

Ten new fluoroquinolone-based GUMBOS were produced through a simple anion exchange reaction incorporating five different anions. Through comprehensive analysis, an equal cation-to-anion ration was confirmed with NMR and FT-IR, providing evidence of the presence of the cation and the anion in GUMBOS. The evaluation of the antibacterial profile of GUMBOS revealed notable antibacterial activity compared to the parent compounds [Cip][HCl] and [Mox][HCl], except for GUMBOS with deoxycholate anions which did not exhibit a strong antibacterial performance towards *S. aureus* and *E. coli*. In addition to this, GUMBOS were non-cytotoxic to L929 fibroblasts and were non-hemolytic to human red blood cells. This innovative approach of combining active pharmaceutical ingredients (APIs) with specific organic ions demonstrates the potential to tailor the biological and physicochemical characteristics of the resulting products. The emergence of these novel solid forms of fluoroquinolones opens up new possibilities for extending their pharmaceutical applications beyond traditional oral formulations. The results obtained in this research underscore the promising pharmacological properties of these compounds and their potential for further investigation in preclinical and clinical studies with the goal of ultimately bringing them to the market as safe and effective therapies for patients in a healthcare setting.

## Figures and Tables

**Table 1:** Yields obtained for [Cip]-based GUMBOS and [Mox]-based GUMBOS.

[Cip]-Based GUMBOS	Yield (%)	[Mox]-Based GUMBOS	Yield (%)
[Cip][BETI]	74%	[Mox][BETI]	69%
[Cip][NTF_2_]	74%	[Mox][NTF_2_]	64%
[Cip][TPB]	52%	[Mox][TPB]	53%
[Cip][Doc]	81%	[Mox][Doc]	67%
[Cip][Dxc]	64%	[Mox][Dxc]	48%

**Table 2:** ESI-MS analysis of synthesized materials.

GUMBOS	Ionic Formula		ESI-MS (Positive Mode)			ESI-MS (Negative Mode)		
	Cation	Anion	Expected Mass (*m/z*)	Experimental Mass (*m/z*)	Mass Error (ppm)	Expected Mass (*m/z*)	Experimental Mass (*m/z*)	Mass Error (ppm)
[Cip][BETI]	C_17_H_18_FN_3_O_3_^+^	C_4_F_10_NO_4_S_2_^-^	332.1410	332.1476	±19.8711	379.9114	379.9124	±2.6322
[Cip][NTF_2_]		C_2_F_6_NO_4_S_2_^-^		332.1414	±1.2043	279.9178	279.9190	±4.2870
[Cip][TPB]		(C_6_H_5_)_4_B^-^		332.1405	±1.5054	319.1663	319.1674	±3.4465
[Cip][Doc]		C_20_H_37_O_7_S^-^		332.1421	±3.3118	421.2260	421.2267	±1.6618
[Cip][Dxc]		C_24_H_39_O_4_^-^		332.1411	±0.3011	391.2853	391.2845	±2.0445
[Mox][BETI]	C_21_H_24_FN_3_O_4_^+^	C_4_F_10_NO_4_S_2_^-^	402.1829	402.1838	±2.2378	379.9109	379.9164	±14.4771
[Mox][NTF_2_]		C_2_F_6_NO_4_S_2_^-^		402.1832	±0.7459	279.9178	279.9188	±3.5725
[Mox][TPB]		(C_6_H_5_)_4_B^-^		402.1841	±2.9837	319.1664	319.1674	±3.1332
[Mox][Doc]		C_20_H_37_O_7_S^-^		402.1843	±3.4810	421.2260	421.2269	±2.1366
[Mox][Dxc]		C_24_H_39_O_4_^-^		402.1841	±2.9837	391.2853	391.2848	±1.2778

**Table 3:** Thermal analysis of GUMBOS’ precursors on DSC.

Precursors	T_onset_	T_peak_
[Cip][HCl]	322.7 °C	325.7 °C
[Mox][HCl]	253.5 °C	257.5 °C
[Li][BETI]	327.8 °C	330.0 °C
[Li][NTF_2_]	151.1 °C	165 °C
[Na][TPB]	64.5 °C	73.7 °C
[Na][Doc]	239.5 °C	282.4 °C
[Na][Dxc]	353.7 °C	355.8 °C

**Table 4:** Thermal analysis of GUMBOS on DSC.

GUMBOS	T_onset_	T_peak_
[Cip][BETI]	210.8 °C	213.2 °C
[Cip][NTF_2_]	202.9 °C	208.0 °C
[Cip][TPB]	112.4 °C	127.6 °C
[Cip][Doc]	269.0 °C	270.6 °C
	275.1 °C	282.9 °C
[Cip][Dxc]	140.2 °C	147.1 °C
	334.9 °C	339.4 °C
[Mox][BETI]	260.2 °C	278.7 °C
[Mox][NTF_2_]	263.3 °C	279.6 °C
[Mox][TPB]	*	*
[Mox][Doc]	269.4 °C	299.3 °C
[Mox][Dxc]	125.3 °C	131.7 °C

* T_onset_ and T_peak_ above differential scanning calorimeter’s maximum threshold of 600 °C.

**Table 5:** Thermal characterization parameters of GUMBOS on TGA.

GUMBOS	T_start_	T_onset_	T_peak_	Residue at 500 °C
[Cip][HCl] *	150 °C	300 °C	318 °C	N/A
[Cip][BETI]	266.25 °C	284.61 °C	294.04 °C	35.43%
[Cip][NTF_2_]	263.57 °C	287.79 °C	295.18 °C	39.88%
[Cip][TPB]	98.94 °C	117.85 °C	122.95 °C	36.93%
[Cip][Doc]	252.18 °C	286.49 °C	286.64 °C	24.26%
[Cip][Dxc]	211.93 °C	326.23 °C	332.30 °C	19.75%
[Mox][HCl] *	220 °C	240 °C	260 °C	N/A
[Mox][BETI]	266.98 °C	307.46 °C	322.97 °C	32.31%
[Mox][NTF_2_]	297.72 °C	319.64 °C	327.51 °C	24.57%
[Mox][TPB]	144.02 °C	176.37 °C	177.21 °C	42.13%
[Mox][Doc]	249.91 °C	271.04 °C	275.90 °C	33.55%
[Mox][Dxc]	208.92 °C	275.22 °C	284.97 °C	15.60%

* Parent compounds data were obtained from [62] for [Cip][HCl] and [45] for [Mox][HCl].

**Table 6:** Logarithm of the octanol/water partition coefficients (log K_O/W_) of GUMBOS.

	${\mathrm{logK}}_{O/W}$ ± SD *	
Anion Counterpart	[Cip]-Based GUMBOS	[Mox]-Based GUMBOS
[BETI]	−0.314 ± 0.094	−0.170 ± 0.116
[NTF_2_]	0.145 ± 0.075	−0.191 ± 0.031
[TPB]	−0.464 ± 0.006	0.487 ± 0.075
[Doc]	0.162 ± 0.015	0.187 ± 0.027
[Dxc]	−1.110 ± 0.018	1.086 ± 0.136

* Data are shown as mean ± standard deviation of two independent experiments. For [Cip], ${\mathrm{logK}}_{O/W}$ = −0.13 [65] and for [Mox], ${\mathrm{logK}}_{O/W}$ = −0.28 [45].

## Data Availability

Not applicable.

## Supplementary Materials

The following supporting information can be downloaded at: https://www.mdpi.com/article/10.3390/ijms242115714/s1.
