# Supplementary material for: Fluoroquinolone-Based Organic Salts (GUMBOS) with Antibacterial Potential
Source: Int J Mol Sci. 2023 Oct 28;24(21):15714. doi: 10.3390/ijms242115714 (PMC10650486; doi:10.3390/ijms242115714)
Supplement: Supplementary file 1 [file ijms-24-15714-s001.zip › ijms-2666158-supplementary.pdf]

Article

Fluoroquinolone-Based Organic Salts (GUMBOS) with Antibacterial Potential

Fábio M. S. Costa [1], Andreia Granja [1], Rocío L. Pérez [2,3], Isiah M. Warner [2,4], Salette Reis [1], Marieta L. C. Passos [1,*] and M. Lúcia M. F. S. Saraiva [1,*]

1 LAQV, REQUIMTE, Laboratory of Applied Pharmacy, Department of Chemical Sciences, Faculty of Pharmacy, Porto University, Rua de Jorge Viterbo Ferreira, 4050-313 Porto, Portugal; up201206985@up.pt (F.M.S.C.); aagranja@ff.up.pt (A.G.); shreis@ff.up.pt (S.R.)
2 Department of Chemistry, Louisiana State University, Baton Rouge, LA 70803, USA; rperez@georgiasouthern.edu (R.L.P.); iwarner@lsu.edu (I.M.W.)
3 Department of Chemistry and Biochemistry, Georgia Southern University, Statesboro, GA 30458, USA
4 Department of Chemistry, Cincinnati University, Cincinnati, OH 45221, USA
* Correspondence: mlpassos@ff.up.pt (M. L.C.P.); lsaraiva@ff.up.pt (M.L.M.F.S.S.); Tel.: +351-220428643 (M.L.C.P.); +351-220428674 (M.L.M.F.S.S.)

Supplementary information

NMR spectra

Parent compounds

Ciprofloxacin hydrochloride [Cip][HCl]

	**Ciprofloxacin [Cip]**
^{1}H	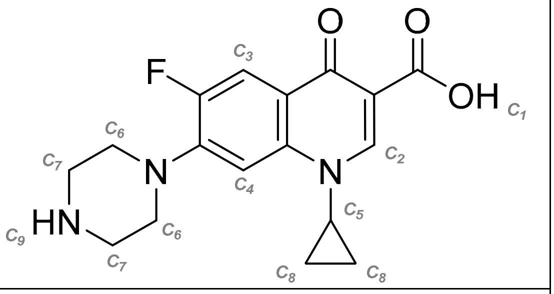
^{13}C	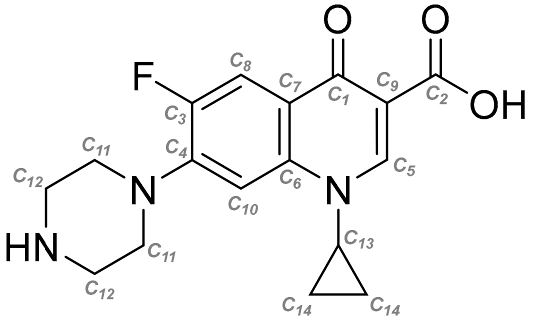

Off-white solid powder, parent material. ^{1}H NMR (400.15 MHz, DMSO-*d*$_6$, ppm) δ 15.11 (s, 1H, C1), 9.41 (s, 2H, NH_2), 8.68 (s, 1H, C2), 7.95 (d, J = 13.19 Hz, 1H, C3), 7.61 (d, J = 7.39 Hz, 1H), 3.86 (septet, J_1 = 2.06 Hz, J_2 = 1.59 Hz, J_3 = 1.87 Hz, 1H, C5), 3.60-3.53 (m, 4H, C6), 3.31 (d, 4H, C7), 1.36-1.29 (m, 2H, C8), 1.23-1.15 (m, 2H, C8). ^{13}C NMR (100.62 MHz, DMSO-*d*$_6$, ppm) δ 176.40 (C1), 165.84 (C2), 152.89 (d, *J* = 249.4 Hz, C3), 148.21 (C4), 144.14 (d, *J* = 10.3 Hz, C5), 139.10

(C6), 119.36 (d, *J* = 7.8 Hz, C7), 111.19 (d, *J* = 23.1 Hz, C8), 106.88 (d, *J* = 6.1 Hz, C9-C10), 46.36 (d, *J* = 4.9 Hz, C11), 42.50 (C12), 35.99 (C13), 7.62 (C14).

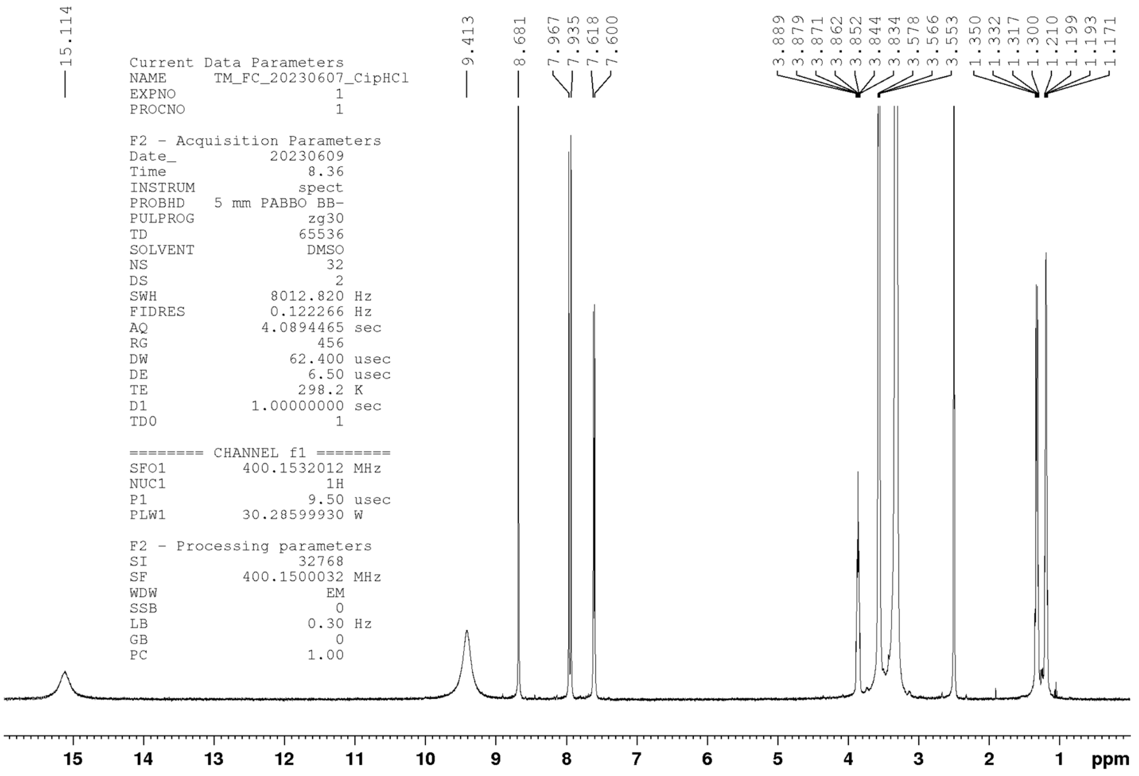

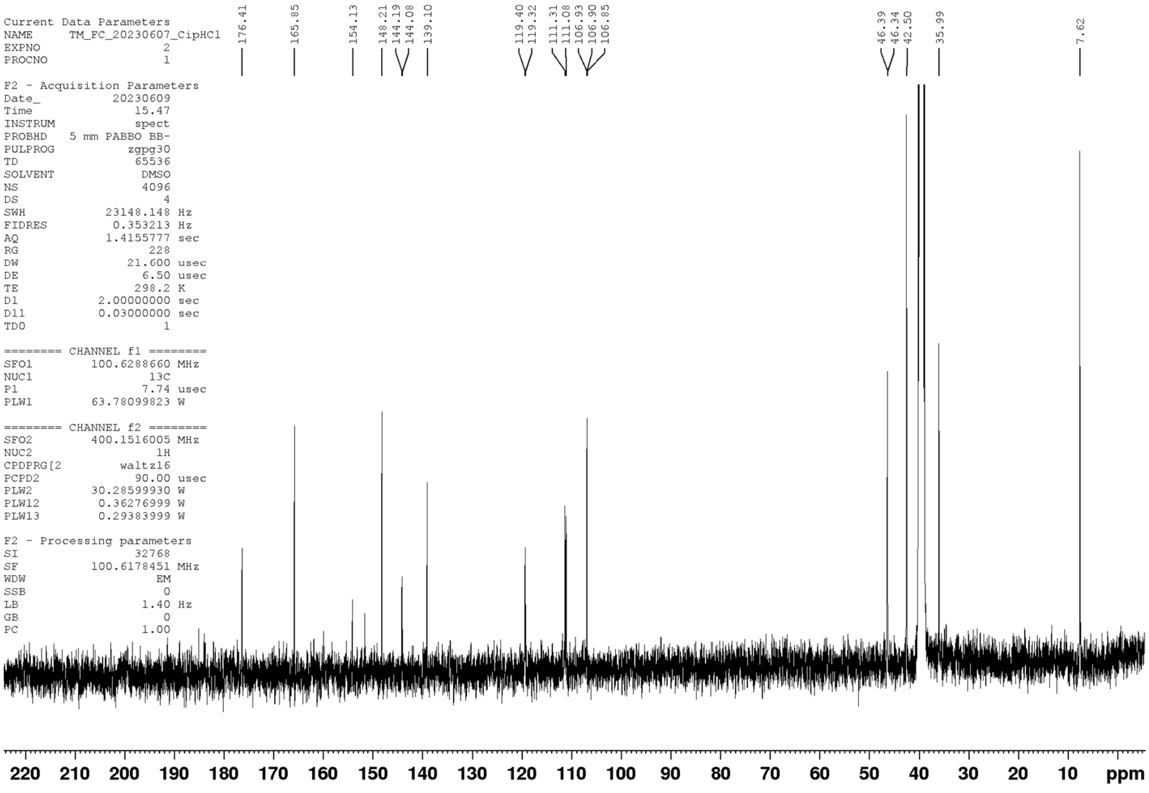

Moxifloxacin hydrochloride [Mox][HCl]

	**Moxifloxacin [Mox]**	**Bis(pentafluoroethanesulfonyl)imide [BETI]**
**^{1}H**		
**^{13}C**		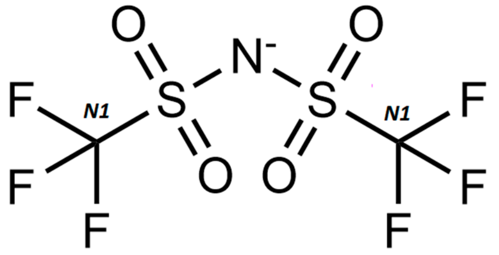

Off-yellow solid powder, parent material. ^{1}H NMR (400.15 MHz, DMSO-d_6, ppm), δ 15.13 (s, 1H, M1), 9.99 (s, 1H, M10), 8.66 (s, 1H, M2), 7.68 (d, J =14.14 Hz, 1H, M3), 4.14 (septet, J_1 = 3.76 Hz, J_2 = 3.22 Hz, J_3 = 4.10 Hz, 1H, M4), 4.10-4.03 (dd, J_1 = 5.49 Hz, J_2 = 12.32 Hz, 1H, M6), 3.91-3.71 (m, 3H, M7 and M9), 3.62-3.58 (m, 3H, M5), 3.20-2.89 (m, 2H, M8), 2.70-2.61 (m, 1H, M12), 1.88-1.65 (m, 4H, M11), 1.24-0.82 (m, 3H, M13), 0.97-0.92 (m, 1H, M13). ^{13}C NMR (100.62 MHz, DMSO-d_6, ppm), δ 176.01 (M1), 165.84 (M2), 153.72-151.24 (M3), 150.36 (M4), 140.41 (M5), 136.68 (M6), 134.51 (M7), 117.35 (M8), 106.66 (M9), 106.42 (M10), 61.86 (M13), 54.46 (M12), 54.11 (M11), 51.87 (M12), 41.51 (M14), 40.61 (M16), 34.13 (M15), 20.50 (M18), 17.52 (M17), 9.57-8.36 (2C, M19).

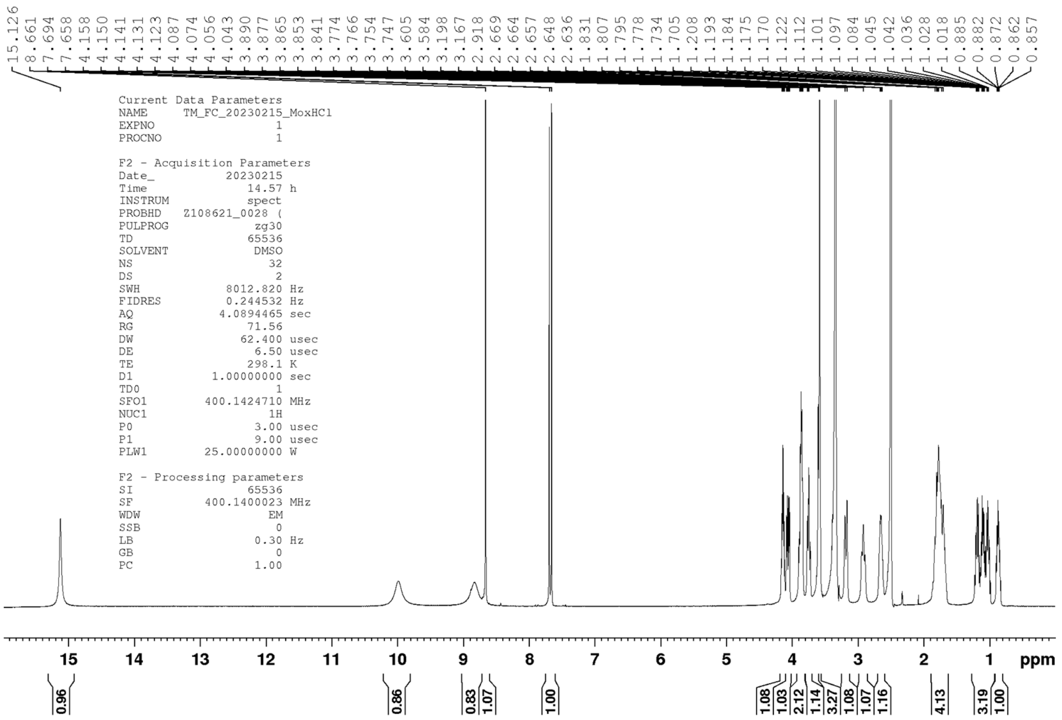
15.126 8.661 7.694 7.658 4.158 4.150 4.141 4.131 4.123 4.087 4.074 4.056 4.043 3.890 3.877 3.865 3.853 3.841 3.774 3.766 3.754 3.747 3.605 3.584 3.198 3.167 2.918 2.669 2.664 2.657 2.648 2.636 1.831 1.807 1.795 1.778 1.734 1.705 1.208 1.193 1.184 1.175 1.170 1.122 1.112 1.101 1.097 1.084 1.045 1.042 1.036 1.028 1.018 0.885 0.882 0.872 0.862 0.857
Current Data Parameters
NAME TM_FC_20230215_MoxHCl
EXPNO 1
PROCNO 1
F2 - Acquisition Parameters
Date_ 20230215
Time 14.57 h
INSTRUM spect
PROBHD Z108621_0028 (
PULPROG zg30
TD 65536
SOLVENT DMSO
NS 32
DS 2
SWH 8012.820 Hz
FIDRES 0.244532 Hz
AQ 4.0894465 sec
RG 71.56
DW 62.400 usec
DE 6.50 usec
TE 298.1 K
D1 1.00000000 sec
TD0 1
SFO1 400.1424710 MHz
NUC1 1H
P0 3.00 usec
P1 9.00 usec
PLW1 25.00000000 W
F2 - Processing parameters
SI 65536
SF 400.1400023 MHz
WDW EM
SSB 0
LB 0.30 Hz
GB 0
PC 1.00
15 14 13 12 11 10 9 8 7 6 5 4 3 2 1 ppm
0.96 0.86 0.83 1.07 1.00 1.08 1.03 2.12 1.14 3.27 1.08 1.07 1.16 4.13 3.19 1.00

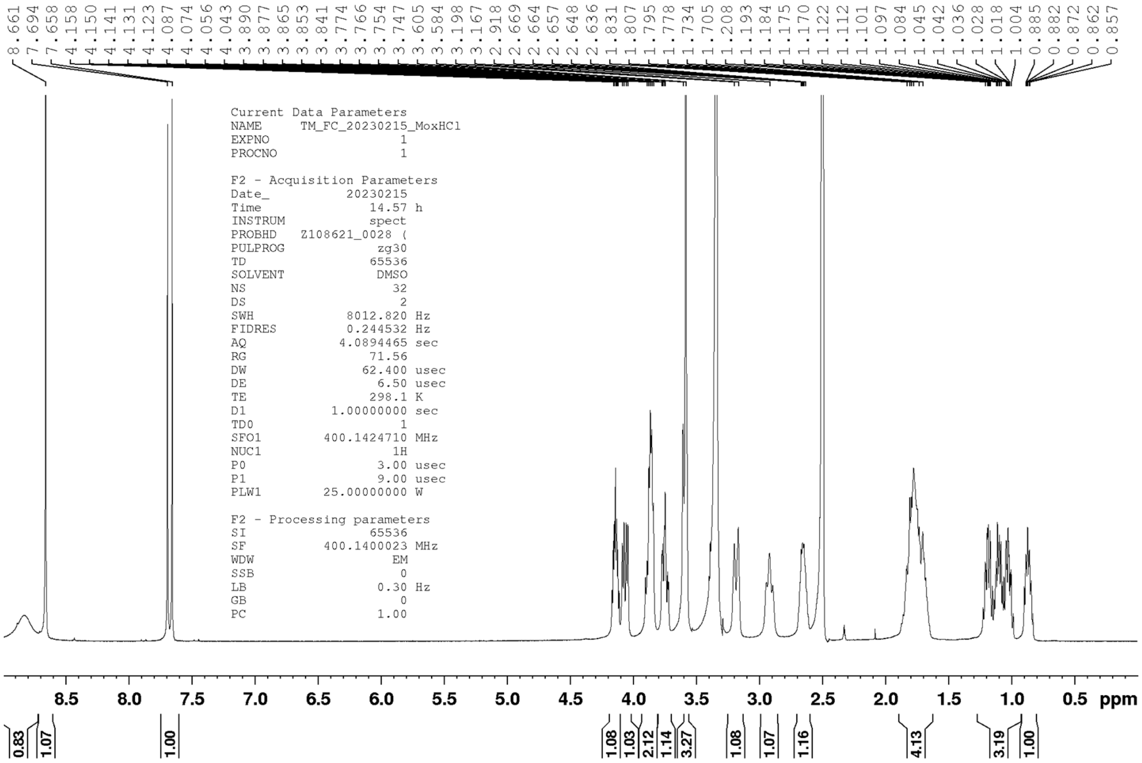
8.661 7.694 7.658 4.158 4.150 4.141 4.131 4.123 4.087 4.074 4.056 4.043 3.890 3.877 3.865 3.853 3.841 3.774 3.766 3.754 3.747 3.605 3.584 3.198 3.167 2.918 2.669 2.664 2.657 2.648 2.636 1.831 1.807 1.795 1.778 1.734 1.705 1.208 1.193 1.184 1.175 1.170 1.122 1.112 1.101 1.097 1.084 1.045 1.042 1.036 1.028 1.018 1.004 0.885 0.882 0.872 0.862 0.857
Current Data Parameters
NAME TM_FC_20230215_MoxHCl
EXPNO 1
PROCNO 1
F2 - Acquisition Parameters
Date_ 20230215
Time 14.57 h
INSTRUM spect
PROBHD Z108621_0028 (
PULPROG zg30
TD 65536
SOLVENT DMSO
NS 32
DS 2
SWH 8012.820 Hz
FIDRES 0.244532 Hz
AQ 4.0894465 sec
RG 71.56
DW 62.400 usec
DE 6.50 usec
TE 298.1 K
D1 1.00000000 sec
TD0 1
SFO1 400.1424710 MHz
NUC1 1H
P0 3.00 usec
P1 9.00 usec
PLW1 25.00000000 W
F2 - Processing parameters
SI 65536
SF 400.1400023 MHz
WDW EM
SSB 0
LB 0.30 Hz
GB 0
PC 1.00
8.5 8.0 7.5 7.0 6.5 6.0 5.5 5.0 4.5 4.0 3.5 3.0 2.5 2.0 1.5 1.0 0.5 ppm
0.83 1.07 1.00 1.08 1.03 2.12 1.14 3.27 1.08 1.07 1.16 4.13 3.19 1.00

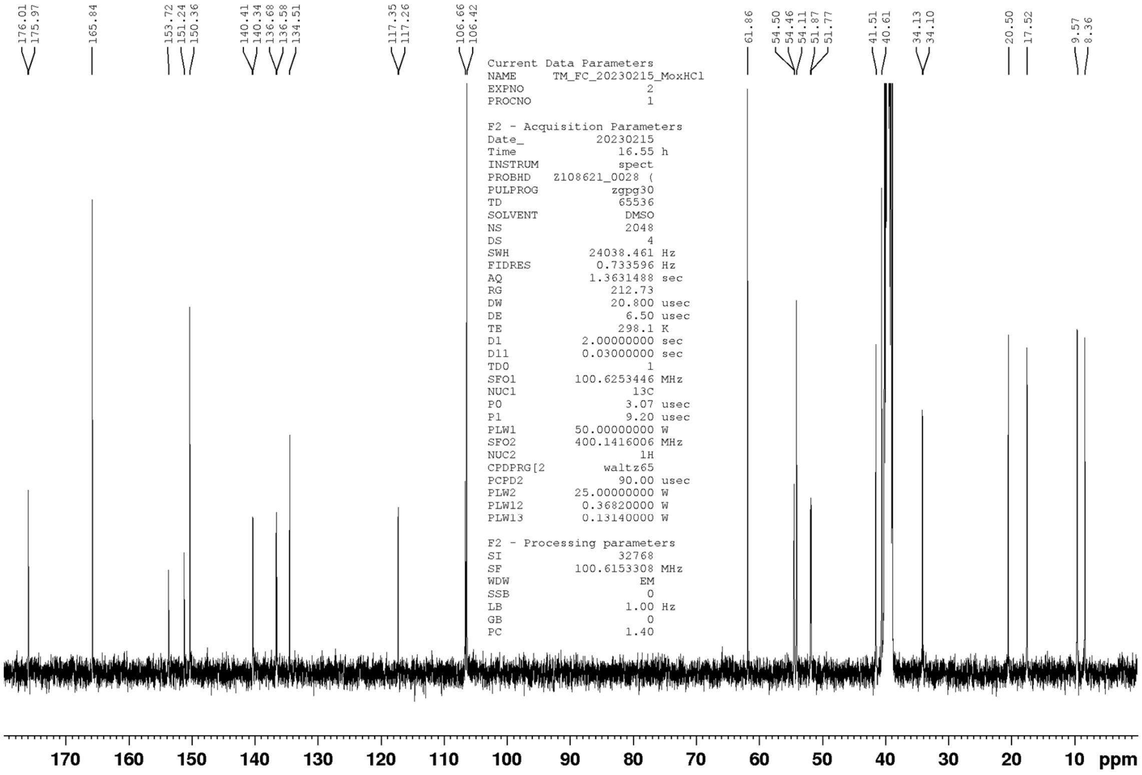
176.01
175.97
165.84
153.72
151.24
150.36
140.41
140.34
136.68
136.58
134.51
117.35
117.26
106.66
106.42
61.86
54.50
54.46
54.11
51.87
51.77
41.51
40.61
34.13
34.10
20.50
17.52
9.57
8.36
Current Data Parameters
NAME TM_FC_20230215_MoxHCl
EXPNO 2
PROCNO 1
F2 - Acquisition Parameters
Date_ 20230215
Time 16.55 h
INSTRUM spect
PROBHD Z108621_0028 (
PULPROG zgpg30
TD 65536
SOLVENT DMSO
NS 2048
DS 4
SWH 24038.461 Hz
FIDRES 0.733596 Hz
AQ 1.3631488 sec
RG 212.73
DW 20.800 usec
DE 6.50 usec
TE 298.1 K
D1 2.00000000 sec
D11 0.03000000 sec
TD0 1
SFO1 100.6253446 MHz
NUC1 13C
P0 3.07 usec
P1 9.20 usec
PLW1 50.00000000 W
SFO2 400.1416006 MHz
NUC2 1H
CPDPRG[2 waltz65
PCPD2 90.00 usec
PLW2 25.00000000 W
PLW12 0.36820000 W
PLW13 0.13140000 W
F2 - Processing parameters
SI 32768
SF 100.6153308 MHz
WDW EM
SSB 0
LB 1.00 Hz
GB 0
PC 1.40
170 160 150 140 130 120 110 100 90 80 70 60 50 40 30 20 10 ppm

Lithium bis(pentafluoroethanesulfonyl)imide [Li][BETI]

	**Bis(pentafluoroethanesulfonyl)imide [BETI]**
**^{1}H**	
**^{13}C**	

Off-white solid powder, parent material. ^{13}C NMR (100.62 MHz, DMSO-d_6, ppm) δ 122.18, 121.84, 119.65, 119.31, 118.98, 116.79, 116.45, 116.12, 114.73, 114.35, 113.97, 113.60, 113.26, 111.81, 111.44, 110.68, 108.90, 108.52, 108.15, 107.77 (m, LiBETI).

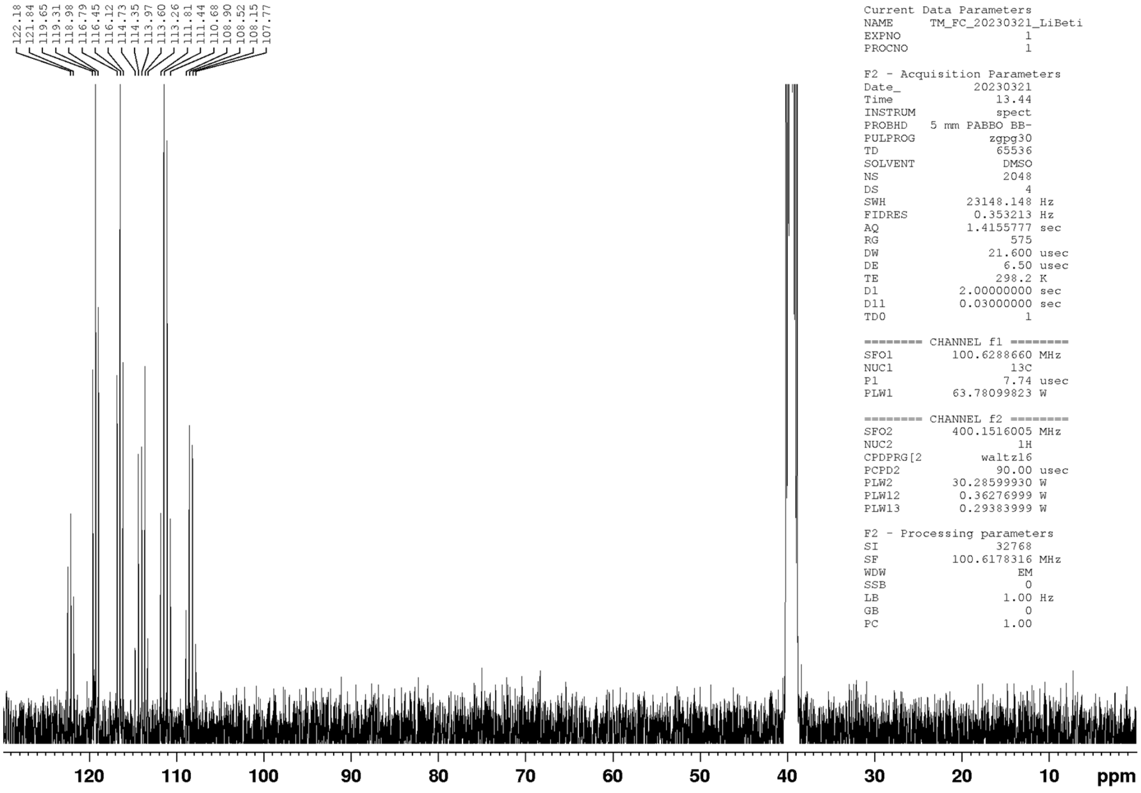

Lithium bis(trifluoromethane)sulfonimide [Li][NTF$_2$]

	**Bis(trifluoromethane)sulfonimide [NTF$_2$]**
**^{1}H**	
**^{13}C**	

Off-white solid powder, parent material. ^{13}C NMR (100.62 MHz, DMSO-d_6, ppm) δ 124.57-121.38-118.18-114.98 (q, J = 321.7 Hz, 2C).

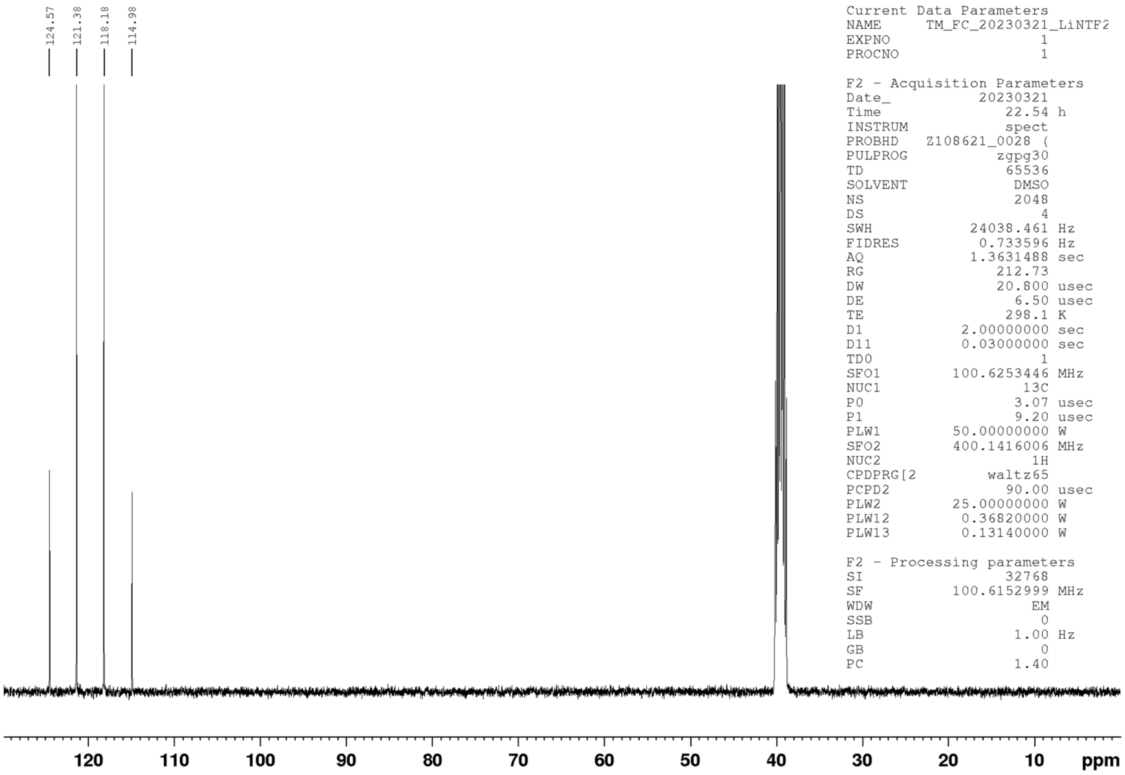

Sodium tetraphenylborate [Na][TPB]

	**Tetraphenylborate [TPB]**
**^{1}H**	
**^{13}C**	

Off-brown solid powder, parent material. ^{1}H NMR (400.15 MHz, DMSO-d_6, ppm) δ 7.26-7.21 (m, 8H, T1), 6.97 (t, J_1 = 7.31 Hz, J_2 = 7.50 Hz, 8H, T2), 6.84 (t, J_1 = 7.10 Hz, J_2 = 7.14 Hz, 4H, T3). ^{13}C NMR (100.62 MHz, DMSO-d_6, ppm) δ 164.18-163.68-163.19-162.72 (8C, T1), 135.62 (8C, T2), 128.99-125.34 (8C, T3), 121.60 (8C, T4).

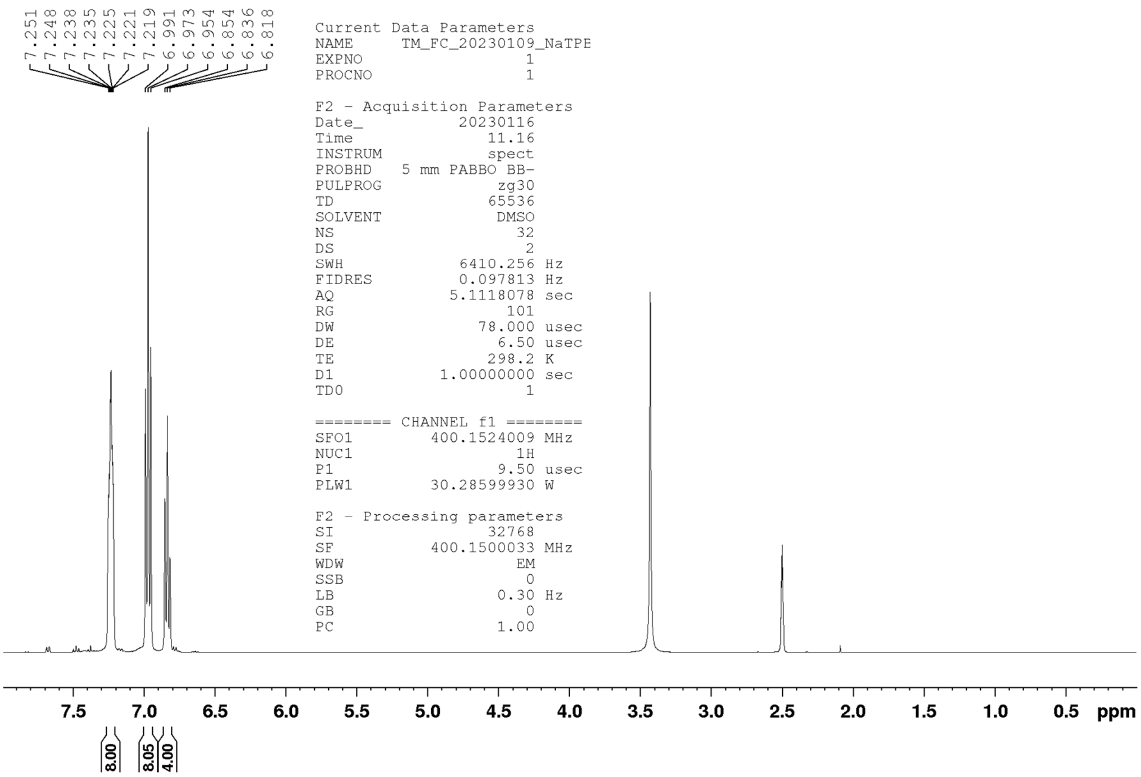

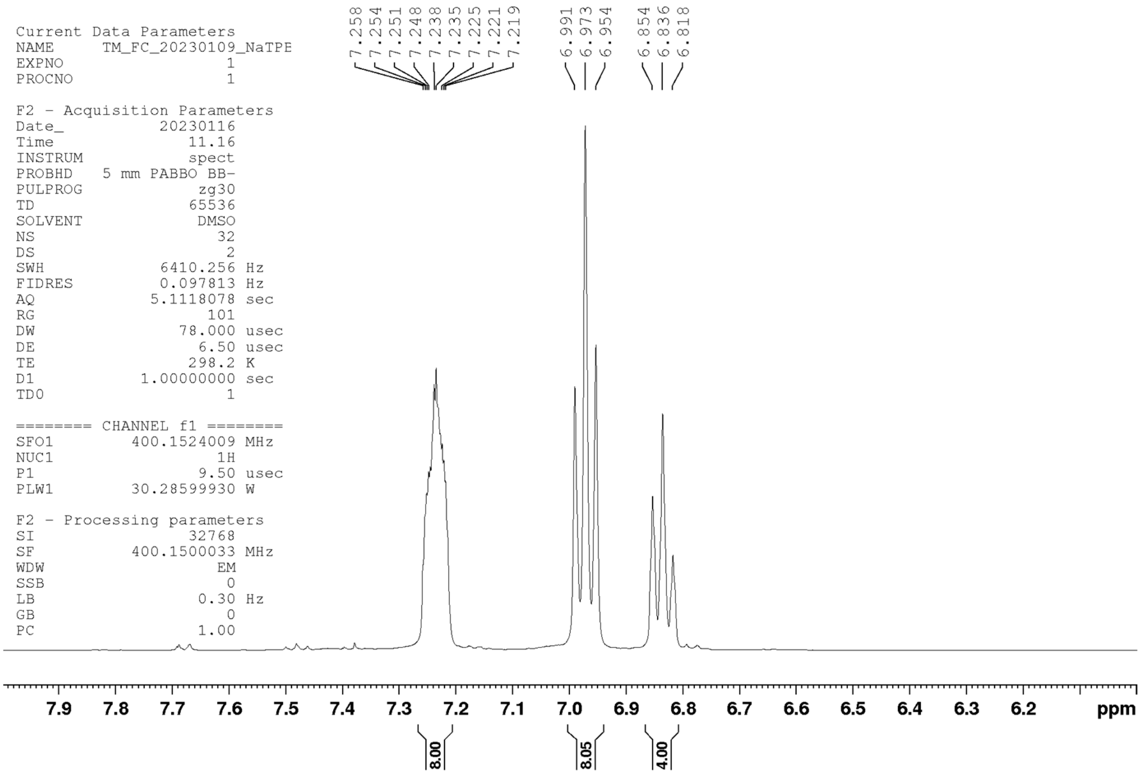
Current Data Parameters
NAME TM_FC_20230109_NaTPE
EXPNO 1
PROCNO 1
F2 - Acquisition Parameters
Date_ 20230116
Time 11.16
INSTRUM spect
PROBHD 5 mm PABBO BB-
PULPROG zg30
TD 65536
SOLVENT DMSO
NS 32
DS 2
SWH 6410.256 Hz
FIDRES 0.097813 Hz
AQ 5.1118078 sec
RG 101
DW 78.000 usec
DE 6.50 usec
TE 298.2 K
D1 1.00000000 sec
TD0 1
======== CHANNEL f1 ========
SFO1 400.1524009 MHz
NUC1 1H
P1 9.50 usec
PLW1 30.28599930 W
F2 - Processing parameters
SI 32768
SF 400.1500033 MHz
WDW EM
SSB 0
LB 0.30 Hz
GB 0
PC 1.00
7.258
7.254
7.251
7.248
7.238
7.235
7.225
7.221
7.219
6.991
6.973
6.954
6.854
6.836
6.818
7.9 7.8 7.7 7.6 7.5 7.4 7.3 7.2 7.1 7.0 6.9 6.8 6.7 6.6 6.5 6.4 6.3 6.2 ppm
8.00
8.05
4.00

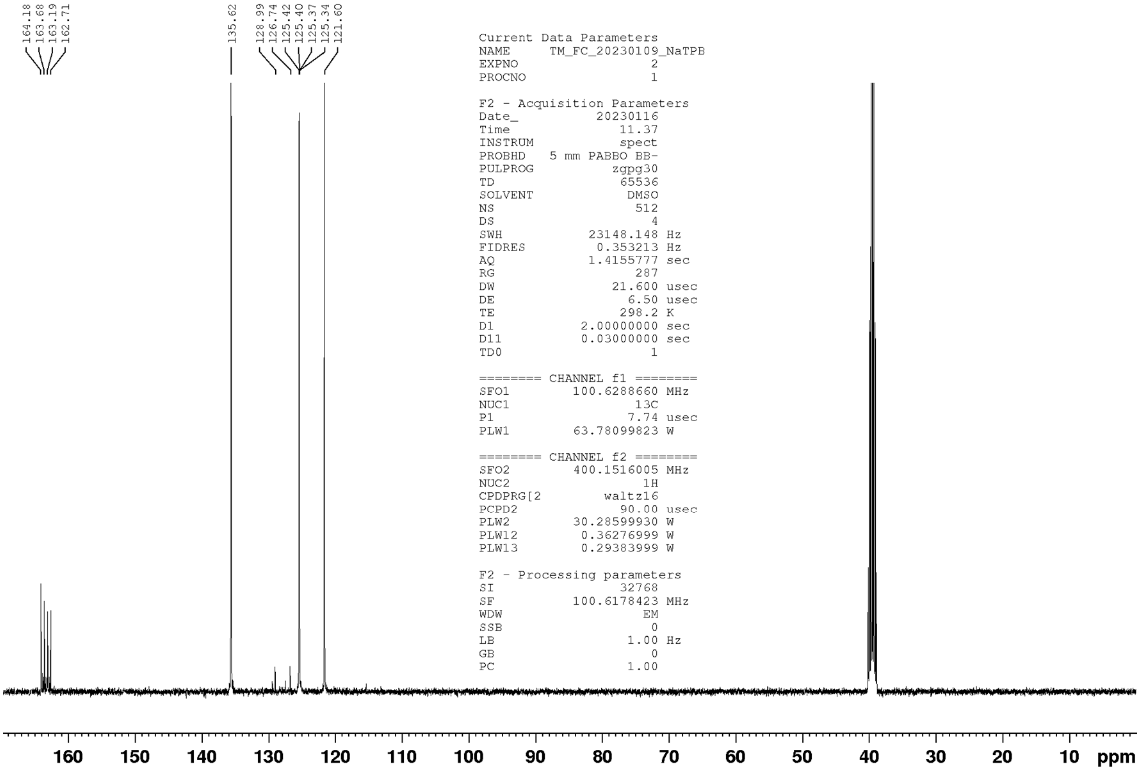
164.18
163.68
163.19
162.71
135.62
128.99
126.74
125.42
125.40
125.37
125.34
121.60
Current Data Parameters
NAME TM_FC_20230109_NaTPB
EXPNO 2
PROCNO 1
F2 - Acquisition Parameters
Date_ 20230116
Time 11.37
INSTRUM spect
PROBHD 5 mm PABBO BB-
PULPROG zgpg30
TD 65536
SOLVENT DMSO
NS 512
DS 4
SWH 23148.148 Hz
FIDRES 0.353213 Hz
AQ 1.4155777 sec
RG 287
DW 21.600 usec
DE 6.50 usec
TE 298.2 K
D1 2.00000000 sec
D11 0.03000000 sec
TD0 1
======== CHANNEL f1 ========
SFO1 100.6288660 MHz
NUC1 13C
P1 7.74 usec
PLW1 63.78099823 W
======== CHANNEL f2 ========
SFO2 400.1516005 MHz
NUC2 1H
CPDPRG[2 waltz16
PCPD2 90.00 usec
PLW2 30.28599930 W
PLW12 0.36276999 W
PLW13 0.29383999 W
F2 - Processing parameters
SI 32768
SF 100.6178423 MHz
WDW EM
SSB 0
LB 1.00 Hz
GB 0
PC 1.00
160 150 140 130 120 110 100 90 80 70 60 50 40 30 20 10 ppm

Sodium docusate [Na][Doc]

	**Docusate [Doc]**
**^{1}H**	
**^{13}C**	

Off-white solid paste, parent material. ^{1}H NMR (400.15 MHz, DMSO-d_6, ppm), δ 3.96-3.81 (m, 4H, D3), 3.65 (dd, J_1 = 11.5 Hz, J_2 = 3.6 Hz, 1H, D2), 2.92 (dd, J_1 = 17.2, J_2 = 11.5 Hz, 1H, D4), 2.80 (dd, J_1 = 17.2 Hz, J_2 = 3.7 Hz, 1H, D4), 1.55-1.43 (m, 2H, D5), 1.41-1.22 (m, 16H, D6-D9), 0.90-0.78 (m, 12H, D10-D11). ^{13}C NMR (100.62 MHz, DMSO-d_6, ppm) δ 171.04 (D1), 168.41 (D2), 66.22-66.15-66.10-66.06 (2C, D3-D4), 61.40 (D5), 38.18-38.14-38.11 (2C, D6), 34.07 (2C, D7), 29.74-29.62-29.56 (2C, D8), 28.36-28.34 (D9), 23.19-23.17-23.01-22.99 (2C, D10), 22.42-22.39 (2C, D11), 13.92-13.89 (2C, D12), 10.81-10.78-10.74 (2C, D13).

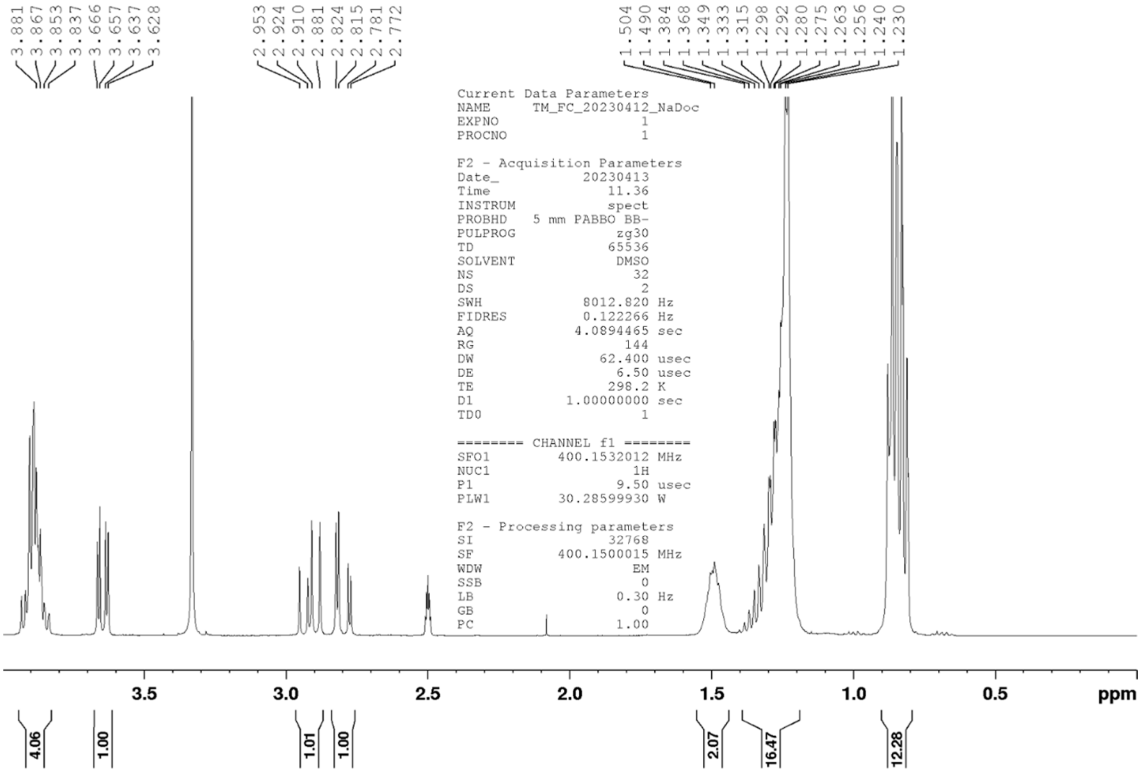

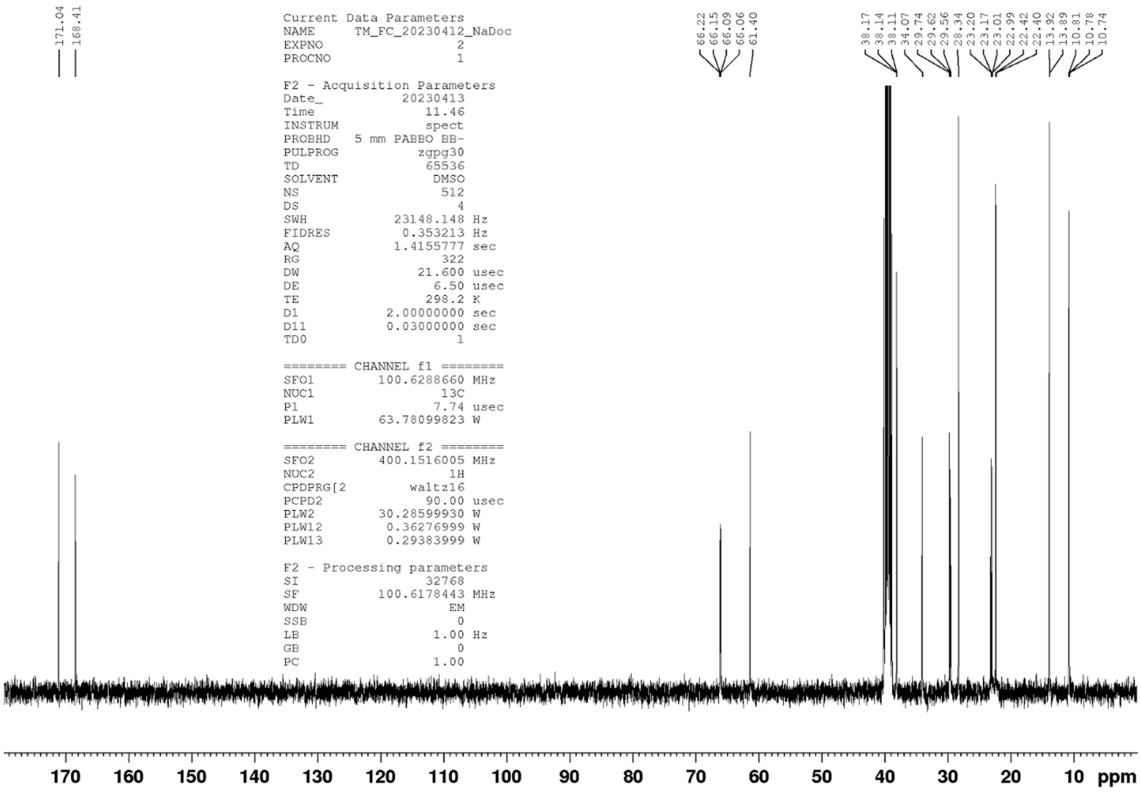
171.04
168.41
Current Data Parameters
NAME TM_FC_20230412_NaDoc
EXPNO 2
PROCNO 1
F2 - Acquisition Parameters
Date_ 20230413
Time 11.46
INSTRUM spect
PROBHD 5 mm PABBO BB-
PULPROG zgpg30
TD 65536
SOLVENT DMSO
NS 512
DS 4
SWH 23148.148 Hz
FIDRES 0.353213 Hz
AQ 1.4155777 sec
RG 322
DW 21.600 usec
DE 6.50 usec
TE 298.2 K
D1 2.00000000 sec
D11 0.03000000 sec
TD0 1
======== CHANNEL f1 ========
SFO1 100.6288660 MHz
NUC1 13C
P1 7.74 usec
PLW1 63.78099823 W
======== CHANNEL f2 ========
SFO2 400.1516005 MHz
NUC2 1H
CPDPRG[2 waltz16
PCPD2 90.00 usec
PLW2 30.28599930 W
PLW12 0.36276999 W
PLW13 0.29383999 W
F2 - Processing parameters
SI 32768
SF 100.6178443 MHz
WDW EM
SSB 0
LB 1.00 Hz
GB 0
PC 1.00
66.22
66.15
66.09
66.06
61.40
38.17
38.14
38.11
34.07
29.74
29.62
29.56
28.34
23.20
23.17
23.01
22.99
22.42
22.40
13.92
13.89
10.81
10.78
10.74
170 160 150 140 130 120 110 100 90 80 70 60 50 40 30 20 10 ppm

Sodium deoxycholate [Na][Dxc]

	**Deoxycholate [Dxc]**
**^{1}H**	
**^{13}C**	

Off-white solid powder, yield 64%. ^{1}H NMR (400.15 MHz, DMSO-d_6, ppm), δ 4.56 (s, 1H, D1), 4.22 (s, 1H, D2), 3.79 (d, *J* = 2.8 Hz, 1H, D4), 3.34 (m, 1H, D4), 1.92-1.01 (m, 24H, D5-D15), 0.89 (d, J = 6.42 Hz, 3H, D16), 0.84 (s, 3H, D17), 0.58 (s, 3H, D18). ^{13}C NMR (100.62 MHz, DMSO-*d_6*) δ 178.01 (D1), 71.12 (D2), 69.98 (D3), 47.48 (D4), 46.60 (D5), 46.00 (D6), 41.67 (D7), 36.33 (D8), 35.71 (D9), 35.59 (D10), 35.48 (D11), 35.20 (D12), 33.86 (D13), 32.95 (D14), 32.82 (D15), 30.25 (D16), 28.65 (D17), 27.35 (D18), 27.04 (D19), 26.16 (D20), 23.62 (D21), 23.13 (D22), 17.30 (D23), 12.57 (D24).

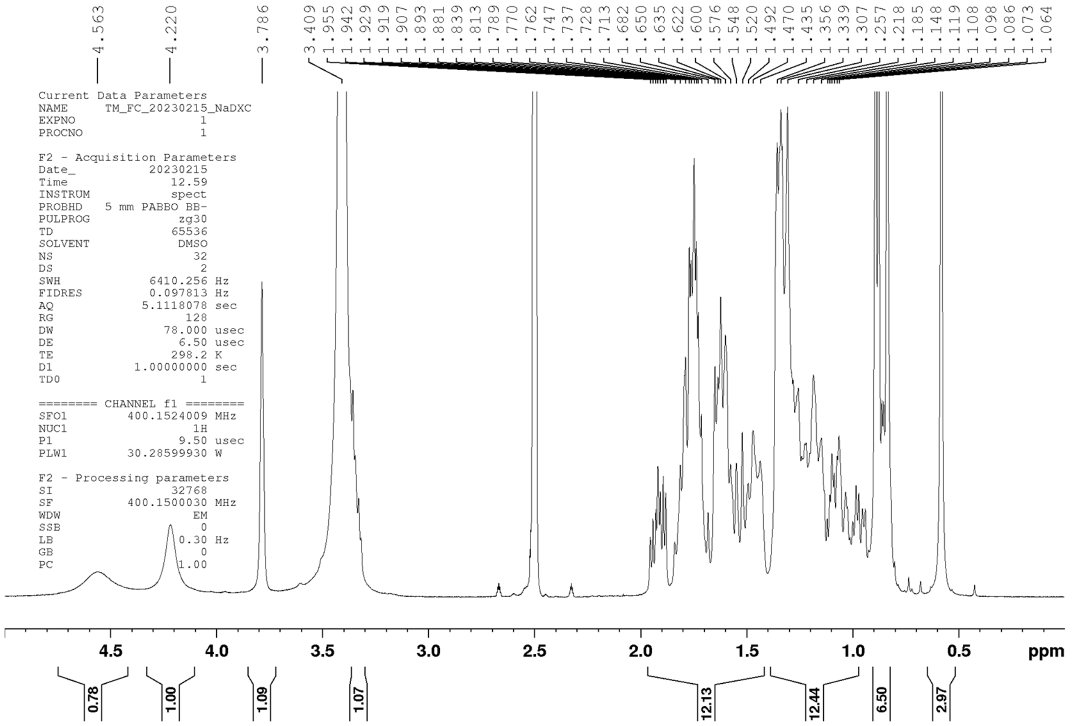

GUMBOS

Ciprofloxacin bis(pentafluoroethanesulfonyl)imide [Cip][BETI]

	**Ciprofloxacin [Cip]**	**Bis(pentafluoroethanesulfonyl)imide [BETI]**
**^{1}H**	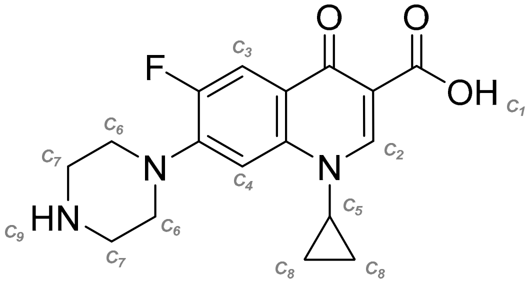	
**^{13}C**	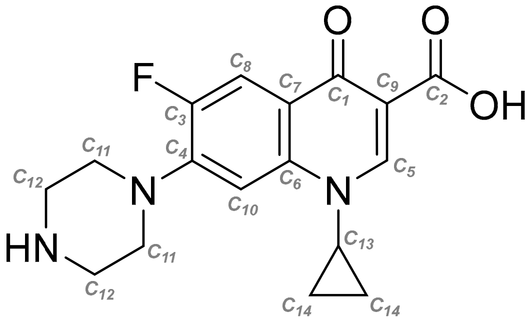	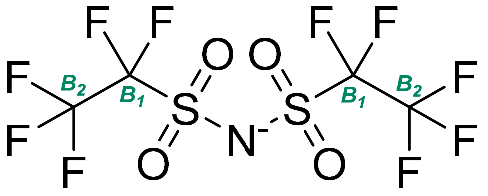

Off-white solid powder, yield 74%. ^{1}H NMR (400.15 MHz, DMSO-*d*$_6$, ppm), δ 15.10 (s, 1H, C1), 8.71 (s, 1H, C2), 7.98 (d, J =13.14 Hz, 1H, C3), 7.62 (d, J = 7.37 Hz, 1H, C4), 3.86 (septet, J_1 = 3.88 Hz, J_2 = 3.10 Hz, J_3 = 4.12 Hz, 1H, C5), 3.65 (m, 4H, C6), 3.36 (m, 4H, C7), 1.33 (m, 2H, C8), 1.21 (m, 2H, C8). ^{13}C NMR (100.62 MHz, DMSO-*d*$_6$, ppm), δ 176.36 (d, J = 2.6 Hz, C1), 165.82 (C2), 152.88 (d, J = 249.4 Hz, C3), 148.17 (C4), 144.05 (d, J = 10.3 Hz, C5), 139.05 (C6), 122.40-121.73 (1C, B1) 119.53–118.87 (m, C7), 116.68-116.01 (1C, B1), 114.23-113.48 (B2), 111.29-110.94 (C8), 108.40-108.03 (1C, B2), 106.93-106.86 (m, 2C, C9-C10), 46.46 (d, J = 5.0 Hz, 2C, C11), 42.75 (C12), 35.95 (C13), 7.60 (C14).

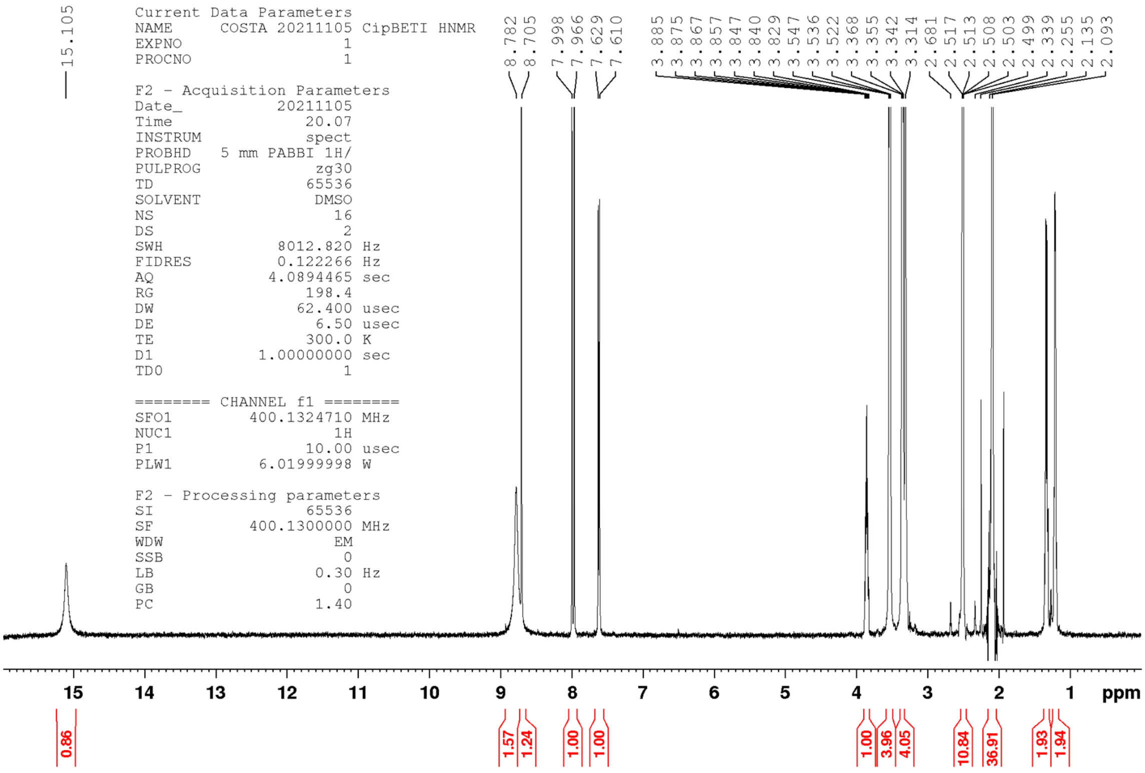
15.105
Current Data Parameters
NAME COSTA 20211105 CipBETI HNMR
EXPNO 1
PROCNO 1
F2 - Acquisition Parameters
Date_ 20211105
Time 20.07
INSTRUM spect
PROBHD 5 mm PABBI 1H/
PULPROG zg30
TD 65536
SOLVENT DMSO
NS 16
DS 2
SWH 8012.820 Hz
FIDRES 0.122266 Hz
AQ 4.0894465 sec
RG 198.4
DW 62.400 usec
DE 6.50 usec
TE 300.0 K
D1 1.00000000 sec
TD0 1
======== CHANNEL f1 ========
SFO1 400.1324710 MHz
NUC1 1H
P1 10.00 usec
PLW1 6.01999998 W
F2 - Processing parameters
SI 65536
SF 400.1300000 MHz
WDW EM
SSB 0
LB 0.30 Hz
GB 0
PC 1.40
8.782 8.705 7.998 7.966 7.629 7.610
3.885 3.875 3.867 3.857 3.847 3.840 3.829 3.547 3.536 3.522 3.368 3.355 3.342 3.314
2.681 2.517 2.513 2.508 2.503 2.499 2.339 2.255 2.135 2.093
15 14 13 12 11 10 9 8 7 6 5 4 3 2 1 ppm
0.86 1.57 1.24 1.00 1.00 1.00 3.96 4.05 10.84 36.91 1.93 1.94

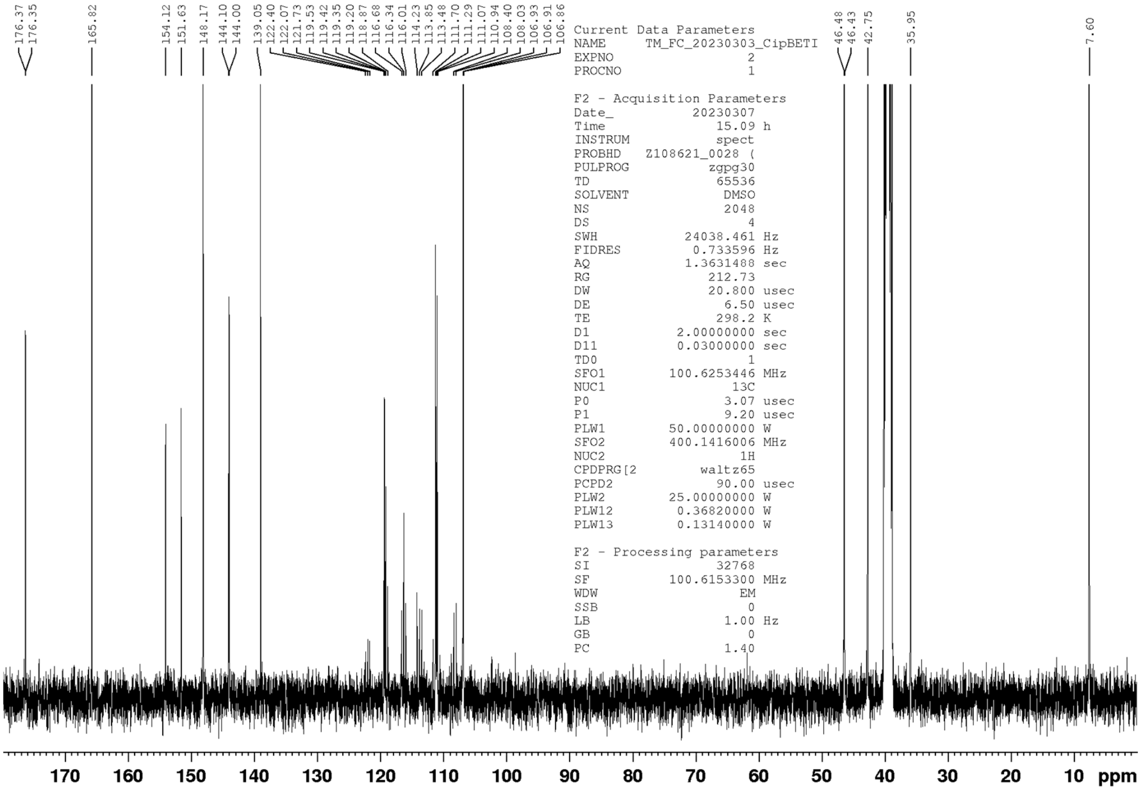
176.37 176.35 165.82 154.12 151.63 148.17 144.10 144.00 139.05 122.40 122.07 121.73 119.53 119.42 119.35 119.20 118.87 116.68 116.34 116.01 114.23 113.85 113.48 111.70 111.29 111.07 110.94 108.40 108.03 106.93 106.91 106.86
46.48 46.43 42.75 35.95 7.60
Current Data Parameters
NAME TM_FC_20230303_CipBETI
EXPNO 2
PROCNO 1
F2 - Acquisition Parameters
Date_ 20230307
Time 15.09 h
INSTRUM spect
PROBHD Z108621_0028 (
PULPROG zgpg30
TD 65536
SOLVENT DMSO
NS 2048
DS 4
SWH 24038.461 Hz
FIDRES 0.733596 Hz
AQ 1.3631488 sec
RG 212.73
DW 20.800 usec
DE 6.50 usec
TE 298.2 K
D1 2.00000000 sec
D11 0.03000000 sec
TD0 1
SFO1 100.6253446 MHz
NUC1 13C
P0 3.07 usec
P1 9.20 usec
PLW1 50.00000000 W
SFO2 400.1416006 MHz
NUC2 1H
CPDPRG[2 waltz65
PCPD2 90.00 usec
PLW2 25.00000000 W
PLW12 0.36820000 W
PLW13 0.13140000 W
F2 - Processing parameters
SI 32768
SF 100.6153300 MHz
WDW EM
SSB 0
LB 1.00 Hz
GB 0
PC 1.40
170 160 150 140 130 120 110 100 90 80 70 60 50 40 30 20 10 ppm

Ciprofloxacin bis(trifluoromethane)sulfonimide [Cip][NTF_2]

	**Ciprofloxacin [Cip]**	**Bis(trifluoromethane)sulfonimide [NTF_2]**
**^{1}H**		
**^{13}C**		

Off-white solid powder, yield 74%. ^{1}H NMR (400.15 MHz, DMSO-d_6, ppm), δ 15.10 (s, 1H, C1), 8.70 (s, 1H, C2), 7.97 (d, J =13.12 Hz, 1H, C3), 7.62 (d, J = 7.47 Hz, 1H, C4), 3.85 (septet, J_1 = 3.78 Hz, J_2 = 3.16 Hz, J_3 = 4.12 Hz, 1H, C5), 3.52 (m, 4H, C6), 3.48 (m, 4H, C7), 1.33 (m, 2H, C8), 1.20 (m, 2H, C8). ^{13}C NMR (100.62 MHz, DMSO-d_6, ppm), δ 176.43 (C1), 165.86 (C2), 151.67 (C3), 148.28 (C4), 144.12 (C5), 139.09 (C6), 121.10 (N1), 119.38 (d, J = 7.7 Hz, C7), 117.90 (N2), 111.18 (d, J = 23.0 Hz, C8), 106.94-106.85 (2C, C9-C10), 46.46 (d, J = 4.9 Hz, 2C, C11), 42.76 (C12), 35.97 (C13), 7.62 (2C, C14).

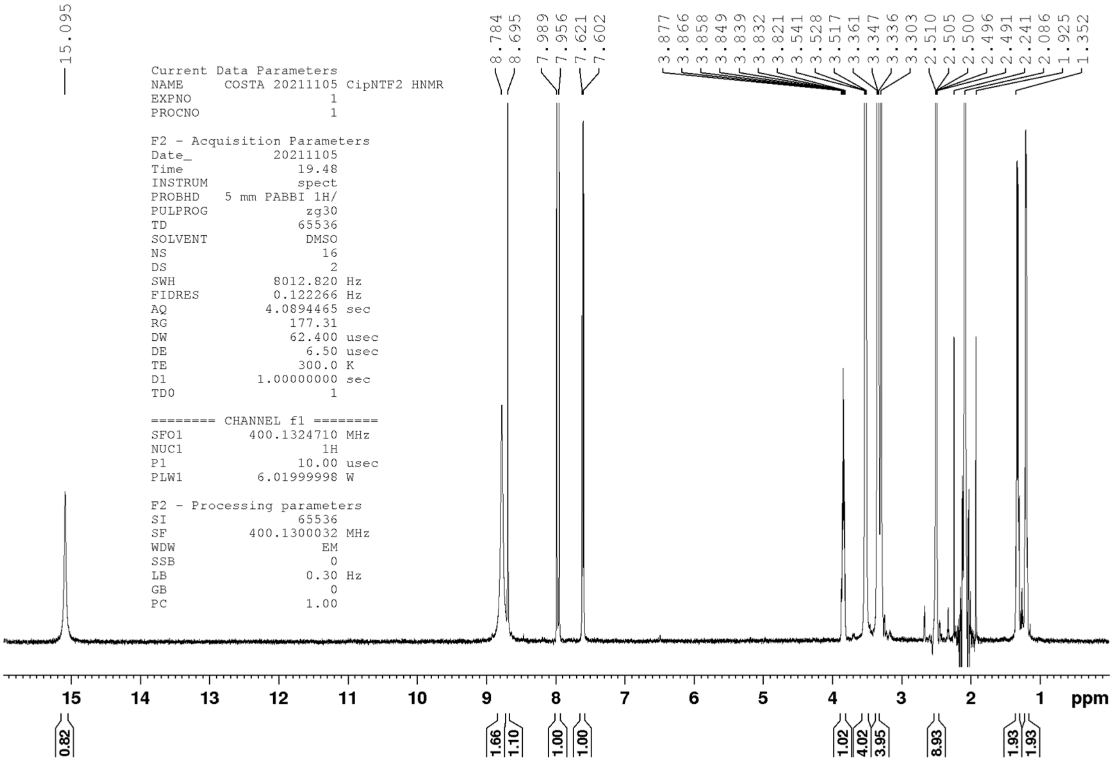

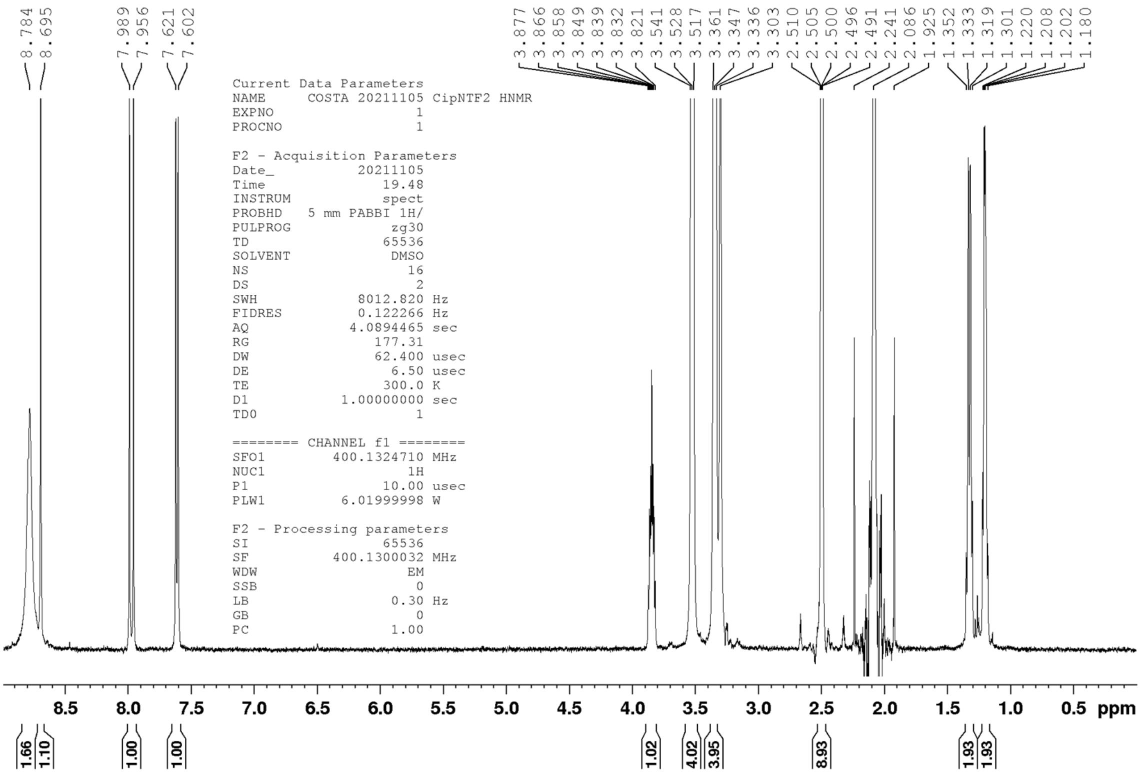
8.784
8.695
7.989
7.956
7.621
7.602
3.877
3.866
3.858
3.849
3.839
3.832
3.821
3.541
3.528
3.517
3.361
3.347
3.336
3.303
2.510
2.505
2.500
2.496
2.491
2.241
2.086
1.925
1.352
1.333
1.319
1.301
1.220
1.208
1.202
1.180
Current Data Parameters
NAME COSTA 20211105 CipNTF2 HNMR
EXPNO 1
PROCNO 1
F2 - Acquisition Parameters
Date_ 20211105
Time 19.48
INSTRUM spect
PROBHD 5 mm PABBI 1H/
PULPROG zg30
TD 65536
SOLVENT DMSO
NS 16
DS 2
SWH 8012.820 Hz
FIDRES 0.122266 Hz
AQ 4.0894465 sec
RG 177.31
DW 62.400 usec
DE 6.50 usec
TE 300.0 K
D1 1.00000000 sec
TD0 1
======== CHANNEL f1 ========
SFO1 400.1324710 MHz
NUC1 1H
P1 10.00 usec
PLW1 6.01999998 W
F2 - Processing parameters
SI 65536
SF 400.1300032 MHz
WDW EM
SSB 0
LB 0.30 Hz
GB 0
PC 1.00
8.5 8.0 7.5 7.0 6.5 6.0 5.5 5.0 4.5 4.0 3.5 3.0 2.5 2.0 1.5 1.0 0.5 ppm
1.66
1.10
1.00
1.00
1.02
4.02
3.95
8.93
1.93
1.93

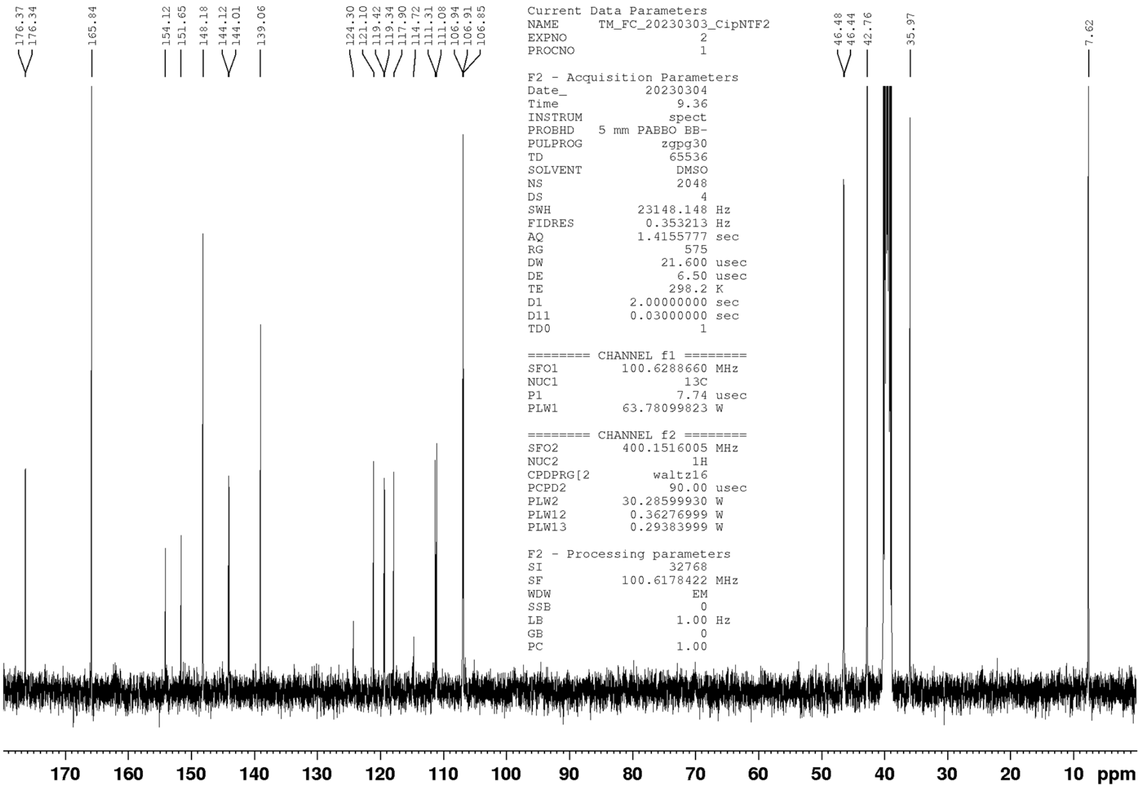
176.37
176.34
165.84
154.12
151.65
148.18
144.12
144.01
139.06
124.30
121.10
119.42
119.34
117.90
114.72
111.31
111.08
106.94
106.91
106.85
46.48
46.44
42.76
35.97
7.62
Current Data Parameters
NAME TM_FC_20230303_CipNTF2
EXPNO 2
PROCNO 1
F2 - Acquisition Parameters
Date_ 20230304
Time 9.36
INSTRUM spect
PROBHD 5 mm PABBO BB-
PULPROG zgpg30
TD 65536
SOLVENT DMSO
NS 2048
DS 4
SWH 23148.148 Hz
FIDRES 0.353213 Hz
AQ 1.4155777 sec
RG 575
DW 21.600 usec
DE 6.50 usec
TE 298.2 K
D1 2.00000000 sec
D11 0.03000000 sec
TD0 1
======== CHANNEL f1 ========
SFO1 100.6288660 MHz
NUC1 13C
P1 7.74 usec
PLW1 63.78099823 W
======== CHANNEL f2 ========
SFO2 400.1516005 MHz
NUC2 1H
CPDPRG[2 waltz16
PCPD2 90.00 usec
PLW2 30.28599930 W
PLW12 0.36276999 W
PLW13 0.29383999 W
F2 - Processing parameters
SI 32768
SF 100.6178422 MHz
WDW EM
SSB 0
LB 1.00 Hz
GB 0
PC 1.00
170 160 150 140 130 120 110 100 90 80 70 60 50 40 30 20 10 ppm

Ciprofloxacin tetraphenylborate [Cip][TPB]

	**Ciprofloxacin [Cip]**	**Tetraphenylborate [TPB]**
**^{1}H**		
**^{13}C**		

Off-white solid powder, yield 52%. ^{1}H NMR (400.15 MHz, DMSO-*d*$_6$, ppm), δ 8.66 (s, 1H, C2), 7.96 (d, J =13.20 Hz, 1H, C3), 7.59 (d, J = 7.49 Hz, 1H, C4), 7.49-7.42 (m, 8H, T1), 6.92 (t, J_1 = 7.18 Hz, J_2 = 7.29 Hz, 8H, T2), 6.78 (t, J_1 = 7.33 Hz, J_2 = 7.44 Hz, 8H, T3), 3.84 (septet, J_1 = 3.70 Hz, J_2 = 3.56 Hz, J_3 = 4.24 Hz, 1H, C5), 3.47-3.43 (m, 4H, C6), 3.26-3.21 (m, 4H,C7), 1.36-1.27 (m, 2H, C8), 1.23-1.15 (m, 2H, C8). ^{13}C NMR (100.63 MHz, DMSO-*d*$_6$) δ 165.89 (C2), 164.09-163.61 -163.12-162.62 (8C, T1), 155.99 (C3), 148.21 (C4), 144.52 (C5), 139.13 (C6), 135.54 (8C, T2), 130.92-126.86-125.30 (8C, T3), 121.52 (8C, T4), 119.86 (C7), 111.06 (C8), 106.84 (C9), 106.77 (C10), 47.55-47.53 (2C, C11), 43.38 (2C, C12), 35.95 (C13), 7.61 (2C, C14).

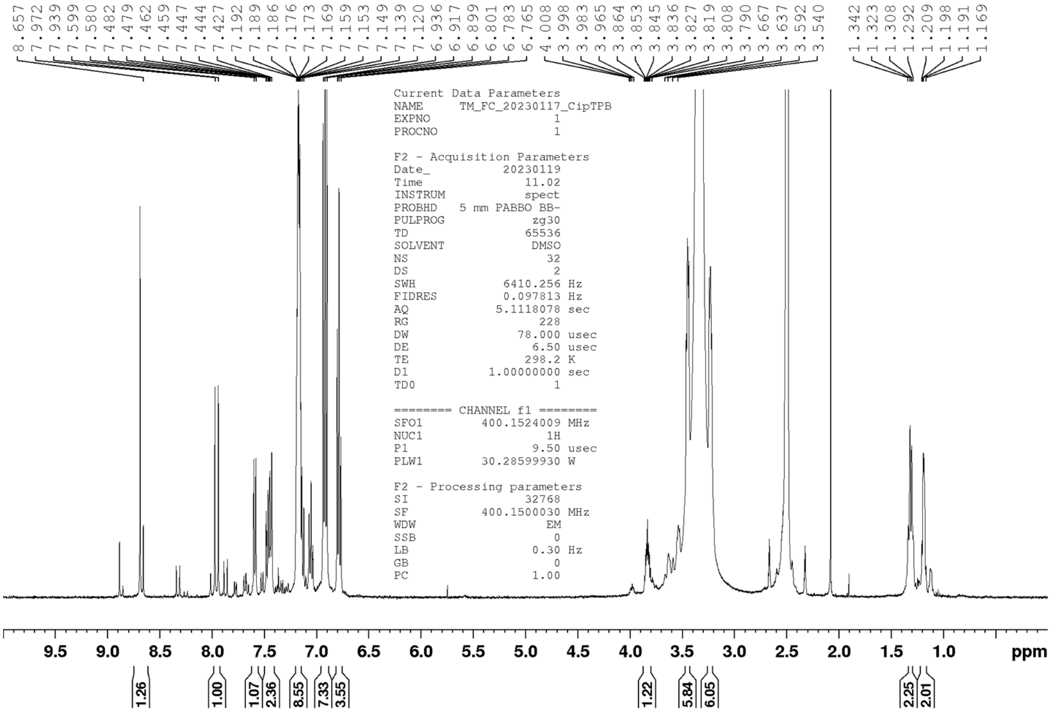

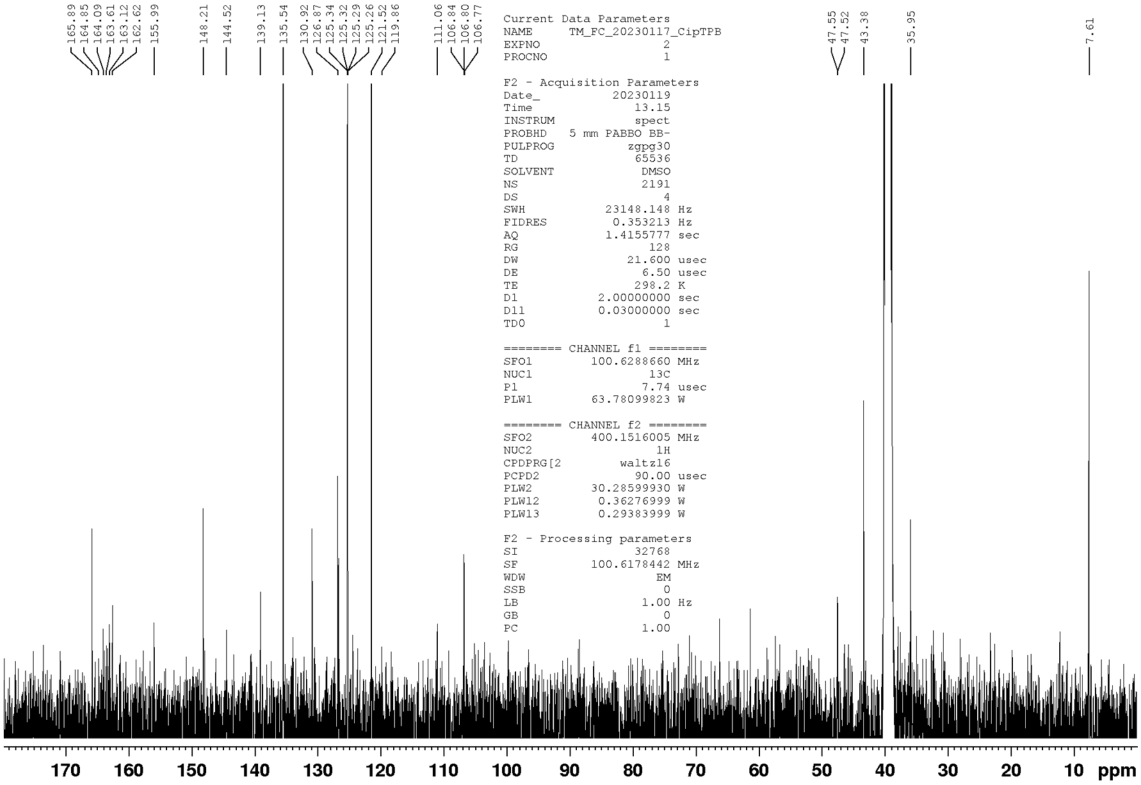
165.89
164.85
164.09
163.61
163.12
162.62
155.99
148.21
144.52
139.13
135.54
130.92
126.87
125.34
125.32
125.29
125.26
121.52
119.86
111.06
106.84
106.80
106.77
Current Data Parameters
NAME TM_FC_20230117_CipTPB
EXPNO 2
PROCNO 1
F2 - Acquisition Parameters
Date_ 20230119
Time 13.15
INSTRUM spect
PROBHD 5 mm PABBO BB-
PULPROG zgpg30
TD 65536
SOLVENT DMSO
NS 2191
DS 4
SWH 23148.148 Hz
FIDRES 0.353213 Hz
AQ 1.4155777 sec
RG 128
DW 21.600 usec
DE 6.50 usec
TE 298.2 K
D1 2.00000000 sec
D11 0.03000000 sec
TD0 1
======== CHANNEL f1 ========
SFO1 100.6288660 MHz
NUC1 13C
P1 7.74 usec
PLW1 63.78099823 W
======== CHANNEL f2 ========
SFO2 400.1516005 MHz
NUC2 1H
CPDPRG[2 waltz16
PCPD2 90.00 usec
PLW2 30.28599930 W
PLW12 0.36276999 W
PLW13 0.29383999 W
F2 - Processing parameters
SI 32768
SF 100.6178442 MHz
WDW EM
SSB 0
LB 1.00 Hz
GB 0
PC 1.00
47.55
47.52
43.38
35.95
7.61
170
160
150
140
130
120
110
100
90
80
70
60
50
40
30
20
10
ppm

Ciprofloxacin docusate [Cip][Doc]

	**Ciprofloxacin [Cip]**	**Docusate [Doc]**
**^{1}H**		
**^{13}C**		

Off-white solid powder, yield 81%. ^{1}H NMR (400.15 MHz, DMSO-d_6, ppm), δ 15.10 (s, 1H, C1), 8.69 (s, 1H, C2), 7.96 (d, J = 13.13 Hz, 1H, C3), 7.61 (d, J = 7.53 Hz, 1H, C4), 3.93-3.82 (m, 5H, C5 and D2), 3.63 (dd, J_1= 3.73 Hz, J_2= 11.40 Hz, 1H, D2), 3.54 (t, J = 4.94 Hz, 4H, C6), 3.35 (t, J = 5.13 Hz, 4H, C7), 2.91 (dd, J_1= 11.55 Hz, J_2= 17.56 Hz, 1H, D4), 2.78 (dd, J1= 3.83 Hz, J2= 17.23 Hz, 1H, D4), 1.53-1.44 (m, 2H, D5), 1.48-1.20 (m, 21H, C8-9 and D5-8), 0.87-0.80 (m, 12H, D9-10). ^{13}C NMR (100.62 MHz, DMSO-d_6, ppm), δ 176.39 (C1), 171.01 (D1), 168.34 (C2), 165.79 (D2), 154.12-151.62 (C3), 148.21 (C4), 144.08 (C5), 139.05 (C6), 119.45 (C7), 111.30 (C8), 111.09 (C9), 106.98 (C10), 66.16 (D3), 66.03 (D4), 61.42 (D5), 46.46 (C11), 42.71 (C12), 38.16 (D6), 35.94 (C13), 34.09 (D7), 29.59 (D9), 28.31 (D8), 23.15 (D10), 22.36 (D11), 13.89 (D12), 10.79 (D13), 7.60 (C14).

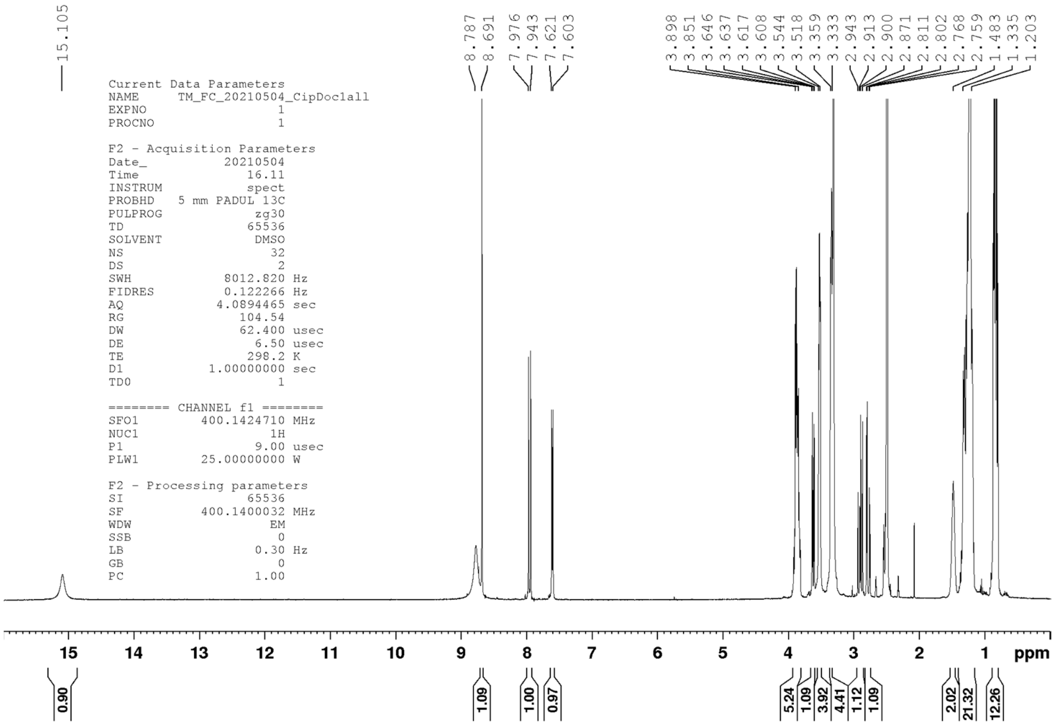

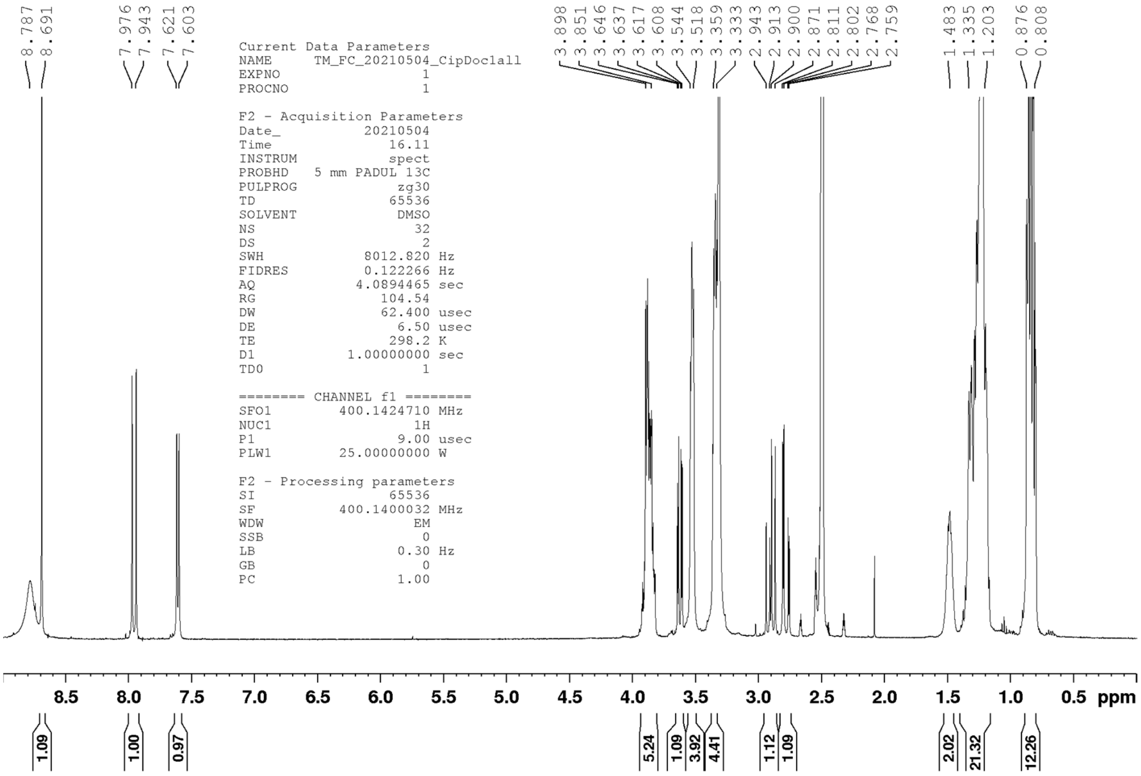
8.787
8.691
7.976
7.943
7.621
7.603
Current Data Parameters
NAME TM_FC_20210504_CipDoclall
EXPNO 1
PROCNO 1
F2 - Acquisition Parameters
Date_ 20210504
Time 16.11
INSTRUM spect
PROBHD 5 mm PADUL 13C
PULPROG zg30
TD 65536
SOLVENT DMSO
NS 32
DS 2
SWH 8012.820 Hz
FIDRES 0.122266 Hz
AQ 4.0894465 sec
RG 104.54
DW 62.400 usec
DE 6.50 usec
TE 298.2 K
D1 1.00000000 sec
TD0 1
======== CHANNEL f1 ========
SFO1 400.1424710 MHz
NUC1 1H
P1 9.00 usec
PLW1 25.00000000 W
F2 - Processing parameters
SI 65536
SF 400.1400032 MHz
WDW EM
SSB 0
LB 0.30 Hz
GB 0
PC 1.00
3.898
3.851
3.646
3.637
3.617
3.608
3.544
3.518
3.359
3.333
2.943
2.913
2.900
2.871
2.811
2.802
2.768
2.759
1.483
1.335
1.203
0.876
0.808
8.5 8.0 7.5 7.0 6.5 6.0 5.5 5.0 4.5 4.0 3.5 3.0 2.5 2.0 1.5 1.0 0.5 ppm
1.09
1.00
0.97
5.24
1.09
3.92
4.41
1.12
1.09
2.02
21.32
12.26

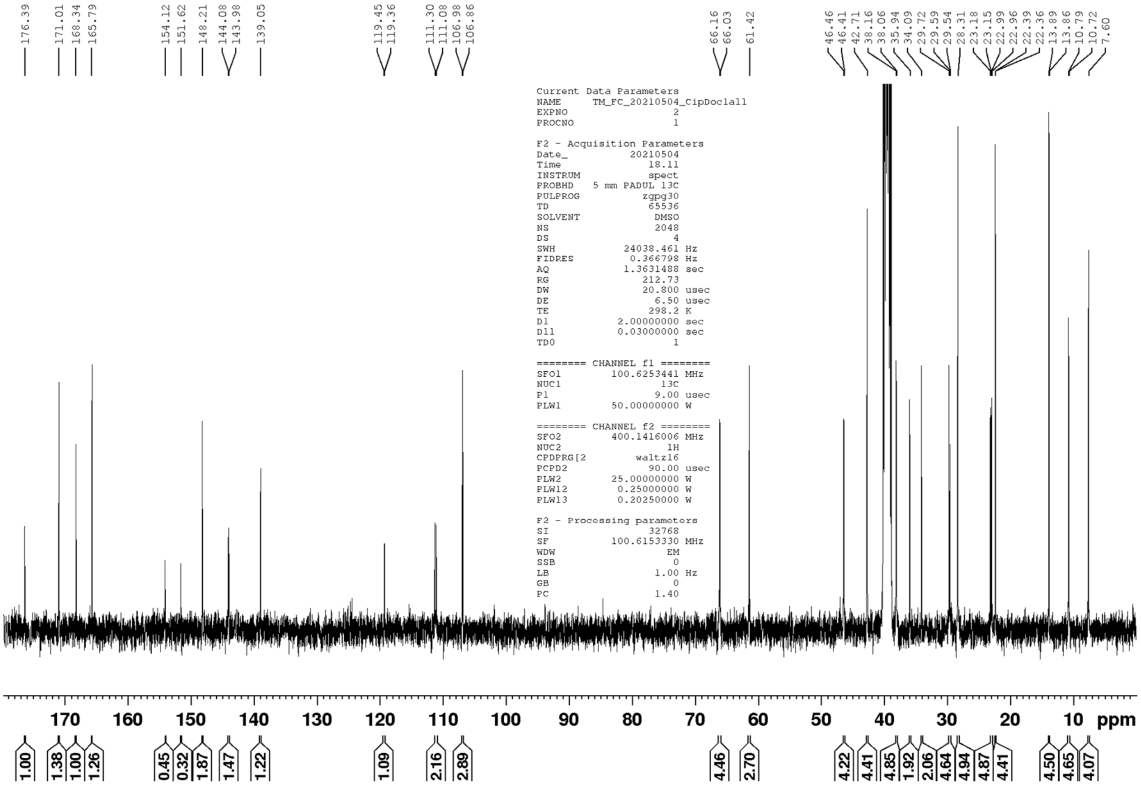
176.39
171.01
168.34
165.79
154.12
151.62
148.21
144.08
143.98
139.05
119.45
119.36
111.30
111.08
106.98
106.86
66.16
66.03
61.42
46.46
46.41
42.71
38.16
38.06
35.94
34.09
29.72
29.59
29.54
28.31
23.18
23.15
22.99
22.96
22.39
22.36
13.89
13.86
10.79
10.72
7.60
Current Data Parameters
NAME TM_FC_20210504_CipDoclall
EXPNO 2
PROCNO 1
F2 - Acquisition Parameters
Date_ 20210504
Time 18.11
INSTRUM spect
PROBHD 5 mm PADUL 13C
PULPROG zgpg30
TD 65536
SOLVENT DMSO
NS 2048
DS 4
SWH 24038.461 Hz
FIDRES 0.366798 Hz
AQ 1.3631488 sec
RG 212.73
DW 20.800 usec
DE 6.50 usec
TE 298.2 K
D1 2.00000000 sec
D11 0.03000000 sec
TD0 1
======== CHANNEL f1 ========
SFO1 100.6253441 MHz
NUC1 13C
P1 9.00 usec
PLW1 50.00000000 W
======== CHANNEL f2 ========
SFO2 400.1416006 MHz
NUC2 1H
CPDPRG[2 waltz16
PCPD2 90.00 usec
PLW2 25.00000000 W
PLW12 0.25000000 W
PLW13 0.20250000 W
F2 - Processing parameters
SI 32768
SF 100.6153330 MHz
WDW EM
SSB 0
LB 1.00 Hz
GB 0
PC 1.40
170 160 150 140 130 120 110 100 90 80 70 60 50 40 30 20 10 ppm
1.00
1.38
1.00
1.26
0.45
0.32
1.87
1.47
1.22
1.09
2.16
2.89
4.46
2.70
4.22
4.41
4.85
1.92
2.06
4.64
4.94
4.87
4.41
4.50
4.65
4.07

Ciprofloxacin deoxycholate [Cip][Dxc]

	**Ciprofloxacin [Cip]**	**Deoxycholate [Dxc]**
**^{1}H**	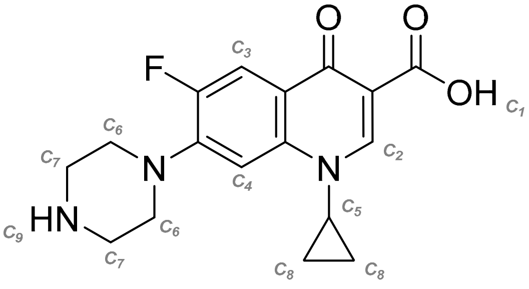	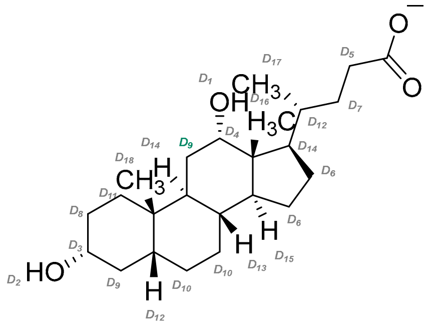
**^{13}C**	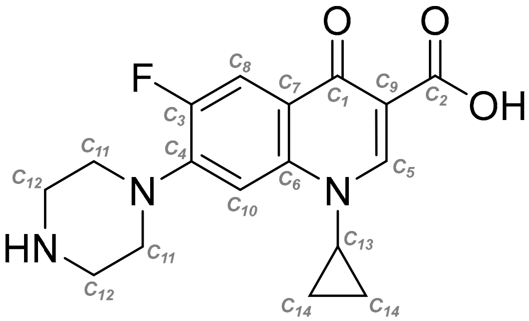	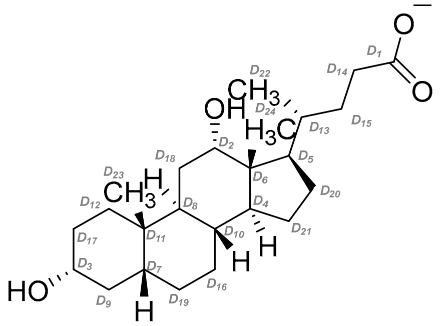

Off-white solid powder, yield 64%. ^{1}H NMR (400.15 MHz, DMSO-d_6, ppm), δ 8.66 (s, 1H, C2), 7.89 (d, J = 13.49 Hz, 1H, C3), 7.54 (d, J = 7.46 Hz, 1H, C4), 4.45 (s, 1H, D1), 4.20 (s, 1H, D2), 3.84 (quin, J = 3.74 Hz, 1H, C5), 3.78 (s, 1H, D3), 3.24 (t, J = 4.74 Hz, 5H, C6 and D4), 2.90 (t, J = 5.05 Hz, 4H, C7), 2.21-2.08 (m, 2H, D5), 1.84-0.96 (m, 28H, C8 and D6-D15, 0.91 (d, J = 6.43 Hz, 3H, D16), 0.84 (s, 3H, D17), 0.59 (s, 3H, D18). ^{13}C NMR (100.62 MHz, DMSO-d_6, ppm), δ 176.34 (D1), 175.00 (C1), 165.97 (C2), 151.86 (C3), 147.92 (C4), 145.71 (C5), 139.26 (C6), 118.33 (C7), 111.03-110.78 (C8), 106.71-106.10-106.06 (2C, C9-C10), 70.98 (D2), 69.92 (D3), 50.72-50.67 (2C, C11), 47.44 (D4), 46.18 (D5), 45.98 (D6), 45.34 (2C, C12), 41.60 (D7), 36.29 (D8), 35.83 (C13), 35.63-35.13-34.96 (D9-D13), 33.80 (D14), 32.90 (D15), 30.96-30.79-30.22 (D16), 28.58 (D17), 27.17 (D18), 26.97 (D19), 26.08 (D20), 23.49 (D21), 23.08 (D22), 16.91 (D23), 12.43 (D24), 7.55 (C14).

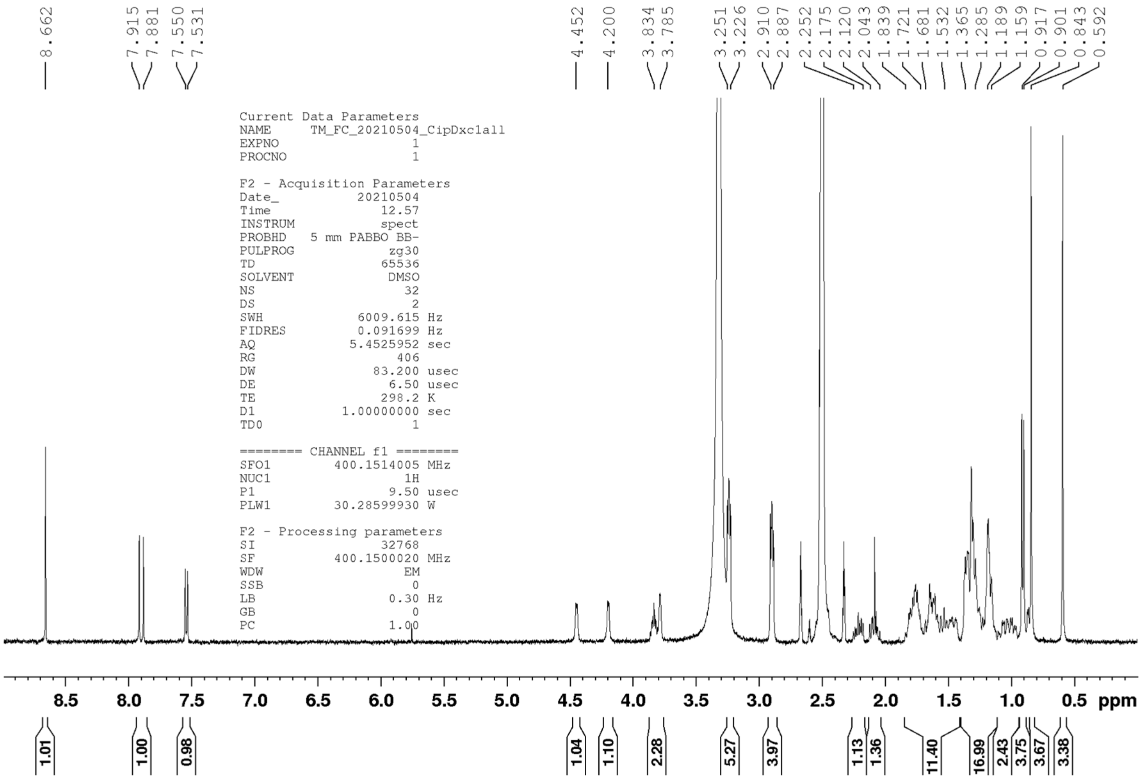

8.662
7.915
7.881
7.550
7.531
4.452
4.200
3.834
3.785
3.251
3.226
2.910
2.887
2.252
2.175
2.120
2.043
1.839
1.721
1.681
1.532
1.365
1.285
1.189
1.159
0.917
0.901
0.843
0.592
Current Data Parameters
NAME TM_FC_20210504_CipDxcl1all
EXPNO 1
PROCNO 1
F2 - Acquisition Parameters
Date_ 20210504
Time 12.57
INSTRUM spect
PROBHD 5 mm PABBO BB-
PULPROG zg30
TD 65536
SOLVENT DMSO
NS 32
DS 2
SWH 6009.615 Hz
FIDRES 0.091699 Hz
AQ 5.4525952 sec
RG 406
DW 83.200 usec
DE 6.50 usec
TE 298.2 K
D1 1.00000000 sec
TD0 1
======== CHANNEL f1 ========
SFO1 400.1514005 MHz
NUC1 1H
P1 9.50 usec
PLW1 30.28599930 W
F2 - Processing parameters
SI 32768
SF 400.1500020 MHz
WDW EM
SSB 0
LB 0.30 Hz
GB 0
PC 1.00
8.5
8.0
7.5
7.0
6.5
6.0
5.5
5.0
4.5
4.0
3.5
3.0
2.5
2.0
1.5
1.0
0.5
ppm
1.01
1.00
0.98
1.04
1.10
2.28
5.27
3.97
1.13
1.36
11.40
16.99
2.43
3.75
3.67
3.38

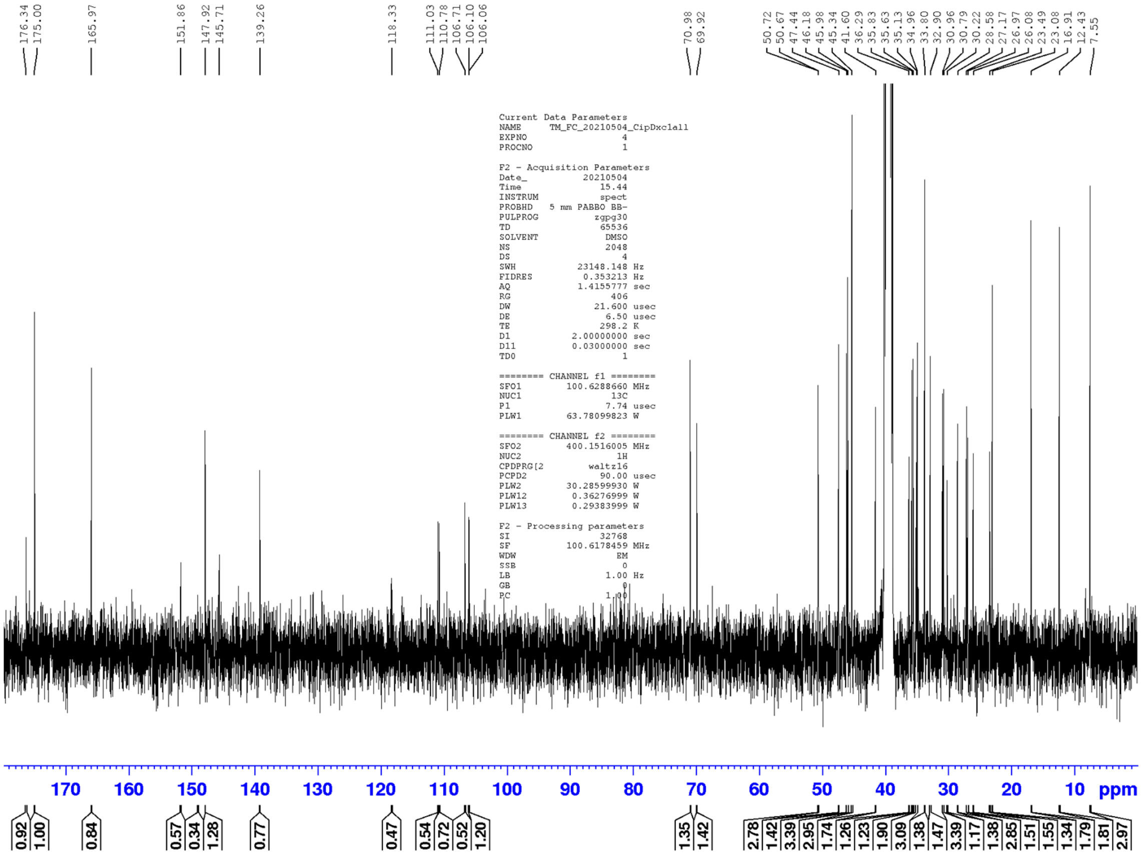

176.34
175.00
165.97
151.86
147.92
145.71
139.26
118.33
111.03
110.78
106.71
106.10
106.06
70.98
69.92
50.72
50.67
47.44
46.18
45.98
45.34
41.60
36.29
35.83
35.63
35.13
34.96
33.80
32.90
30.96
30.79
30.22
28.58
27.17
26.97
26.08
23.49
23.08
16.91
12.43
7.55
Current Data Parameters
NAME TM_FC_20210504_CipDxcl1all
EXPNO 4
PROCNO 1
F2 - Acquisition Parameters
Date_ 20210504
Time 15.44
INSTRUM spect
PROBHD 5 mm PABBO BB-
PULPROG zgpg30
TD 65536
SOLVENT DMSO
NS 2048
DS 4
SWH 23148.148 Hz
FIDRES 0.353213 Hz
AQ 1.4155777 sec
RG 406
DW 21.600 usec
DE 6.50 usec
TE 298.2 K
D1 2.00000000 sec
D11 0.03000000 sec
TD0 1
======== CHANNEL f1 ========
SFO1 100.6288660 MHz
NUC1 13C
P1 7.74 usec
PLW1 63.78099823 W
======== CHANNEL f2 ========
SFO2 400.1516005 MHz
NUC2 1H
CPDPRG[2 waltz16
PCPD2 90.00 usec
PLW2 30.28599930 W
PLW12 0.36276999 W
PLW13 0.29383999 W
F2 - Processing parameters
SI 32768
SF 100.6178459 MHz
WDW EM
SSB 0
LB 1.00 Hz
GB 0
PC 1.00
170
160
150
140
130
120
110
100
90
80
70
60
50
40
30
20
10
ppm
0.92
1.00
0.84
0.57
0.34
1.28
0.77
0.47
0.54
0.72
0.52
1.20
1.35
1.42
2.78
1.42
3.39
2.95
1.74
1.26
1.23
1.90
3.09
1.38
1.47
3.39
1.17
1.38
2.85
1.51
1.55
1.34
1.79
1.81
2.97

Moxifloxacin bis(pentafluoroethanesulfonyl)imide [Mox][BETI]

	**Moxifloxacin [Mox]**	**Bis(pentafluoroethanesulfonyl)imide [BETI]**
**^{1}H**	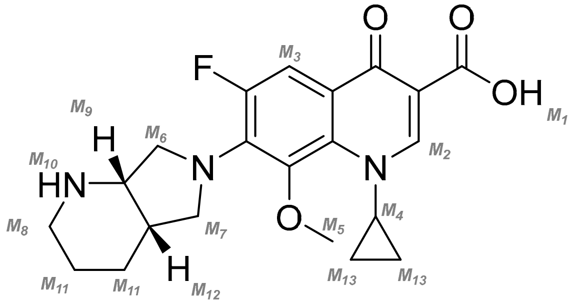	
**^{13}C**	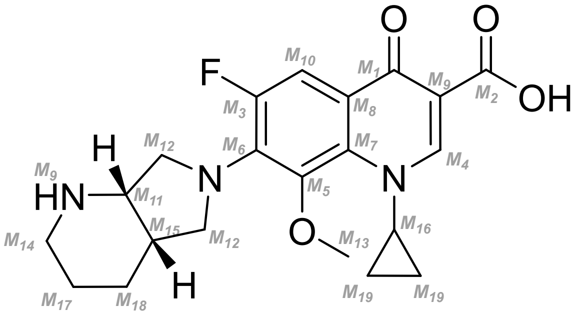	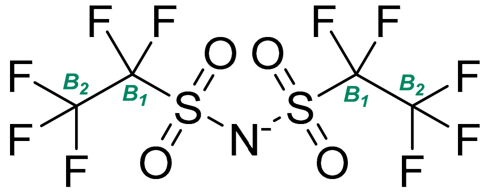

Off-yellow solid powder, yield 64%. ^{1}H NMR (400.15 MHz, DMSO-d_6, ppm), δ 15.10 (s, 1H, M1), 8.68 (s, 1H, M2), 7.71 (d, J =14.09 Hz, 1H, M3), 4.14 (septet, J_1 = 3.76 Hz, J_2 = 3.32 Hz, J_3 = 4.04 Hz, 1H, M4), 4.05 (dd, J_1 = 5.51 Hz, J_2 = 12.14 Hz, 1H, M6), 3.92-3.72 (m, 3H, M6-M7 and M9), 3.57 (s, 2H, M5), 3.50 (d, J = 12.05 Hz, 1H, M7), 3.29-3.21 (m, 1H, M8), 3.03-2.88 (m, 1H, M8), 2.71-2.62 (m, 1H, M12), 1.89-1.65 (m, 4H, M11), 1.24-0.83 (m, 3H, M13), 0.92-0.83 (m, 1H, M13). ^{13}C NMR (100.62 MHz, DMSO-d_6, ppm), δ 176.05 (M1), 165.81 (M2), 153.82-151.32 (M3), 150.45 (M4), 140.61 (M5), 136.56 (M6), 134.50 (M7), 119.21 (2C, B1), 117.56 (M8), 116.37 (2C, B2), 106.71 (M10), 106.47 (M9), 61.81 (M13), 54.50 (M12), 54.42 (M11), 51.65 (M12), 41.90 (M14), 40.62 (M16), 34.15 (M15), 20.49 (M18), 17.63 (M17), 9.56-8.38 (2C, M19).

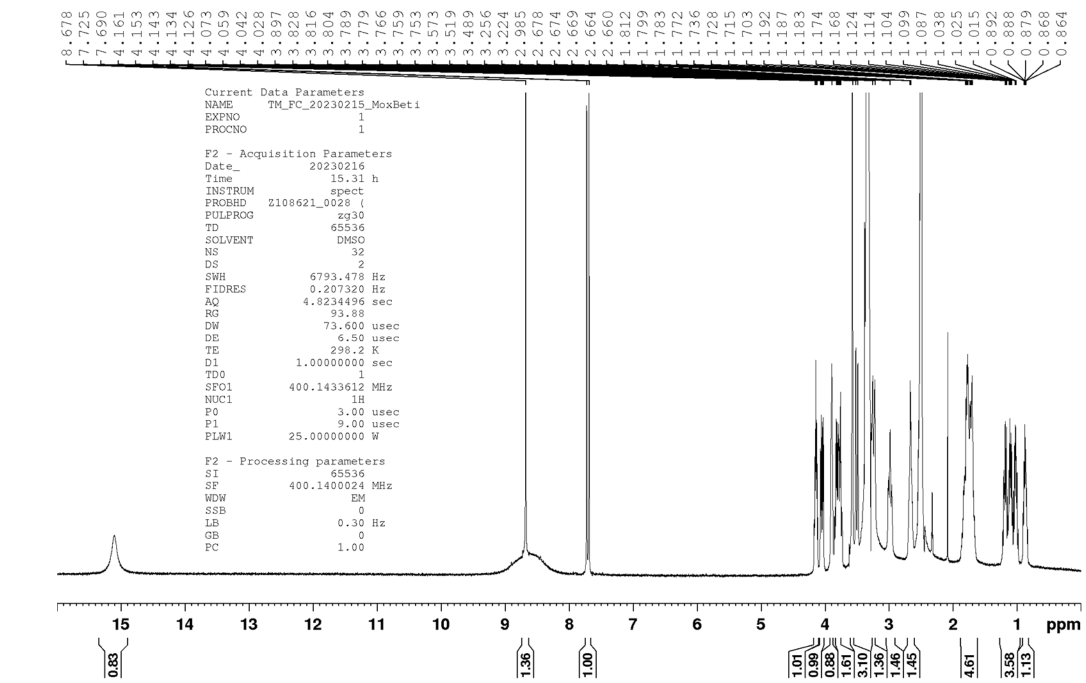
8.678
7.725
7.690
4.161
4.153
4.143
4.134
4.126
4.073
4.059
4.042
4.028
3.897
3.828
3.816
3.804
3.789
3.779
3.766
3.759
3.753
3.573
3.519
3.489
3.256
3.224
2.985
2.678
2.674
2.669
2.664
2.660
1.812
1.799
1.783
1.772
1.736
1.728
1.715
1.703
1.192
1.187
1.183
1.174
1.168
1.124
1.114
1.104
1.099
1.087
1.038
1.025
1.015
0.892
0.888
0.879
0.868
0.864
Current Data Parameters
NAME TM_FC_20230215_MoxBeti
EXPNO 1
PROCNO 1
F2 - Acquisition Parameters
Date_ 20230216
Time 15.31 h
INSTRUM spect
PROBHD Z108621_0028 (
PULPROG zg30
TD 65536
SOLVENT DMSO
NS 32
DS 2
SWH 6793.478 Hz
FIDRES 0.207320 Hz
AQ 4.8234496 sec
RG 93.88
DW 73.600 usec
DE 6.50 usec
TE 298.2 K
D1 1.00000000 sec
TD0 1
SFO1 400.1433612 MHz
NUC1 1H
P0 3.00 usec
P1 9.00 usec
PLW1 25.00000000 W
F2 - Processing parameters
SI 65536
SF 400.1400024 MHz
WDW EM
SSB 0
LB 0.30 Hz
GB 0
PC 1.00
15 14 13 12 11 10 9 8 7 6 5 4 3 2 1 ppm
0.83
1.36
1.00
1.01
0.99
0.88
1.61
3.10
1.36
1.46
1.45
4.61
3.58
1.13

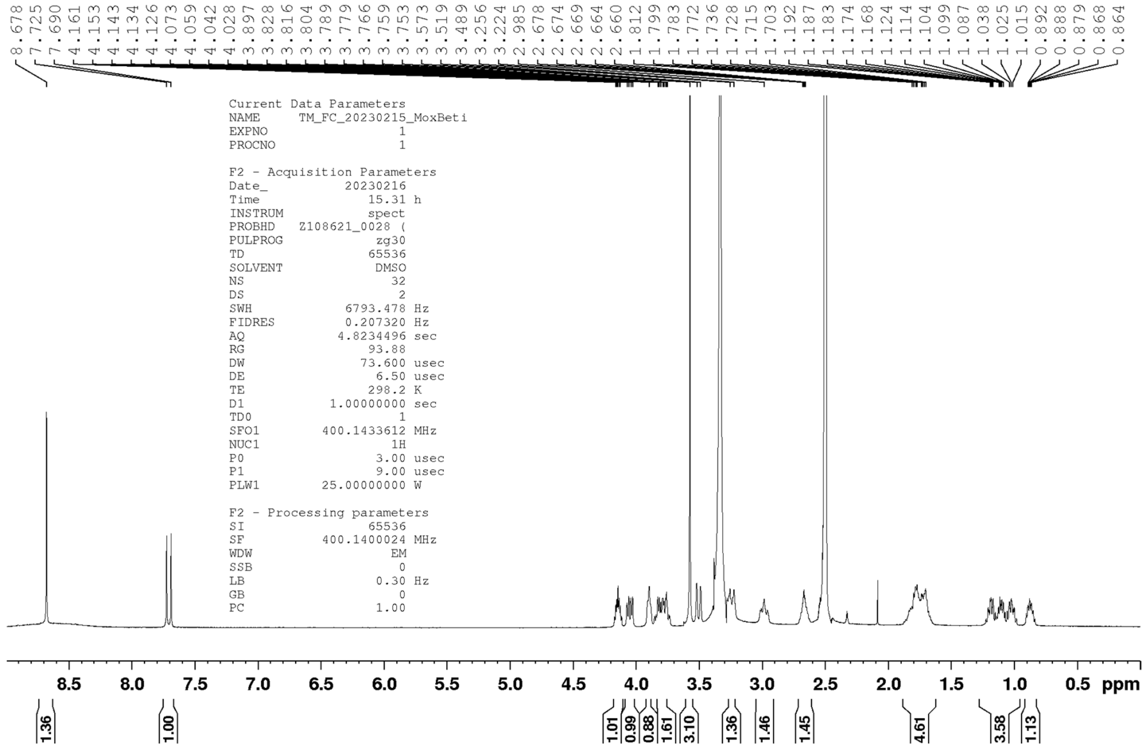
8.678
7.725
7.690
4.161
4.153
4.143
4.134
4.126
4.073
4.059
4.042
4.028
3.897
3.828
3.816
3.804
3.789
3.779
3.766
3.759
3.753
3.573
3.519
3.489
3.256
3.224
2.985
2.678
2.674
2.669
2.664
2.660
1.812
1.799
1.783
1.772
1.736
1.728
1.715
1.703
1.192
1.187
1.183
1.174
1.168
1.124
1.114
1.104
1.099
1.087
1.038
1.025
1.015
0.892
0.888
0.879
0.868
0.864
Current Data Parameters
NAME TM_FC_20230215_MoxBeti
EXPNO 1
PROCNO 1
F2 - Acquisition Parameters
Date_ 20230216
Time 15.31 h
INSTRUM spect
PROBHD Z108621_0028 (
PULPROG zg30
TD 65536
SOLVENT DMSO
NS 32
DS 2
SWH 6793.478 Hz
FIDRES 0.207320 Hz
AQ 4.8234496 sec
RG 93.88
DW 73.600 usec
DE 6.50 usec
TE 298.2 K
D1 1.00000000 sec
TD0 1
SFO1 400.1433612 MHz
NUC1 1H
P0 3.00 usec
P1 9.00 usec
PLW1 25.00000000 W
F2 - Processing parameters
SI 65536
SF 400.1400024 MHz
WDW EM
SSB 0
LB 0.30 Hz
GB 0
PC 1.00
8.5 8.0 7.5 7.0 6.5 6.0 5.5 5.0 4.5 4.0 3.5 3.0 2.5 2.0 1.5 1.0 0.5 ppm
1.36
1.00
1.01
0.99
0.88
1.61
3.10
1.36
1.46
1.45
4.61
3.58
1.13

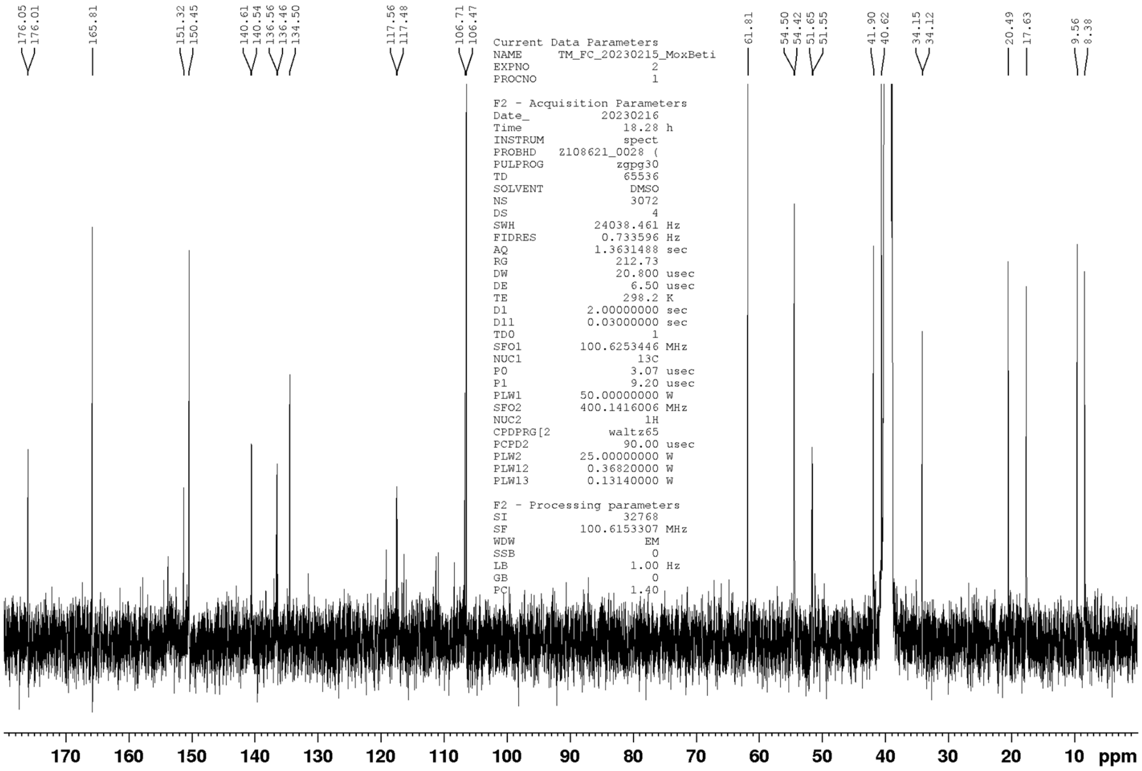
176.05
176.01
165.81
151.32
150.45
140.61
140.54
136.56
136.46
134.50
117.56
117.48
106.71
106.47
61.81
54.50
54.42
51.65
51.55
41.90
40.62
34.15
34.12
20.49
17.63
9.56
8.38
Current Data Parameters
NAME TM_FC_20230215_MoxBeti
EXPNO 2
PROCNO 1
F2 - Acquisition Parameters
Date_ 20230216
Time 18.28 h
INSTRUM spect
PROBHD Z108621_0028 (
PULPROG zgpg30
TD 65536
SOLVENT DMSO
NS 3072
DS 4
SWH 24038.461 Hz
FIDRES 0.733596 Hz
AQ 1.3631488 sec
RG 212.73
DW 20.800 usec
DE 6.50 usec
TE 298.2 K
D1 2.00000000 sec
D11 0.03000000 sec
TD0 1
SFO1 100.6253446 MHz
NUC1 13C
P0 3.07 usec
P1 9.20 usec
PLW1 50.00000000 W
SFO2 400.1416006 MHz
NUC2 1H
CPDPRG[2 waltz65
PCPD2 90.00 usec
PLW2 25.00000000 W
PLW12 0.36820000 W
PLW13 0.13140000 W
F2 - Processing parameters
SI 32768
SF 100.6153307 MHz
WDW EM
SSB 0
LB 1.00 Hz
GB 0
PC 1.40
170 160 150 140 130 120 110 100 90 80 70 60 50 40 30 20 10 ppm

Moxifloxacin bis(trifluoromethane)sulfonamide (MoxNTF$_2$)

	**Moxifloxacin [Mox]**	**Bis(trifluoromethane)sulfonamide [NTF$_2$]**
**^{1}H**		
**^{13}C**		

Off-yellow solid powder, yield 64%. ^{1}H NMR (400.15 MHz, DMSO-d_6, ppm), δ 15.10 (s, 1H, M1), 8.68 (s, 1H, M2), 7.97 (d, J =14.16 Hz, 1H, M3), 4.18-4.09 (m, 1H, M4), 4.05 (dd, J1 = 5.03 Hz, J2 = 12.41 Hz, 1H, M6), 3.93-3.70 (m, 3H, M7 and M9), 3.58 (m, 3H, M5), 3.05-2.93 (m, 2H, M8), 2.66 (s, 1H, M12), 1.89-1.59 (m, 4H, M11), 1.24-0.83 (m, 4H, M13). ^{13}C NMR (400.15 MHz, DMSO-d_6, ppm), δ 176.04 (d, *J* = 3.1 Hz, M1), 165.86 (M2), 153.83-151.36 (M3), 150.45 (M4), 140.58 (M5), 136.57 (M6), 134.53 (M7), 121.10 (N1), 117.90 (N2), 117.47 (M8), 106.71 (M10), 106.47 (M9), 61.80 (M11), 54.67 (M12), 54.52 (M11), 51.63 (M12), 42.02 (M14), 40.64 (M16), 34.24 (M15), 20.60 (M18), 17.80 (M17), 9.59-8.38 (2C, M19).

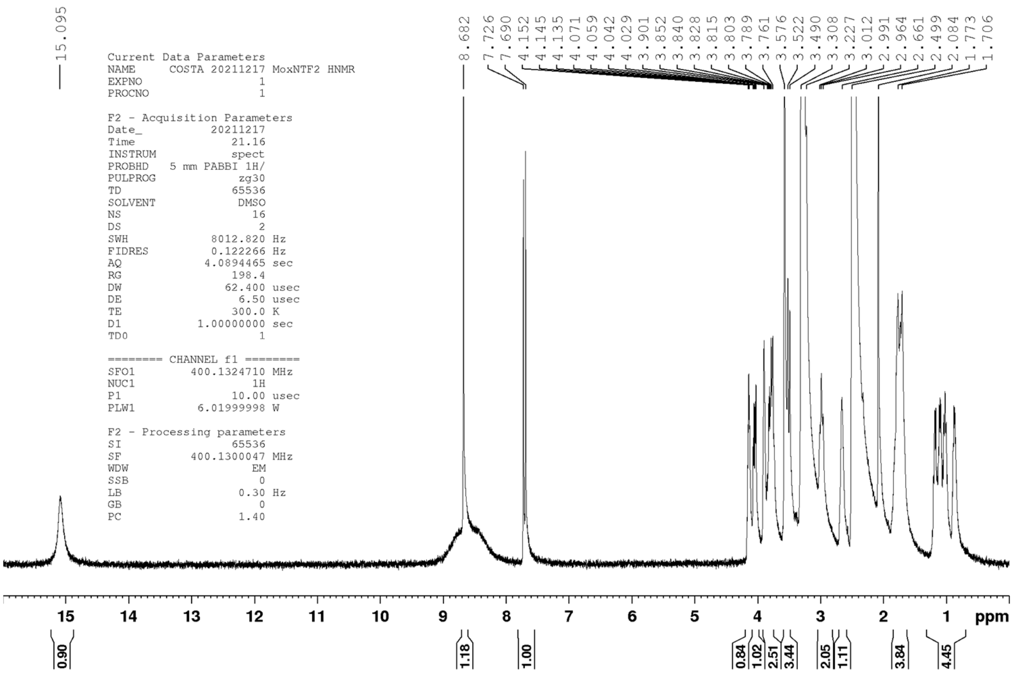

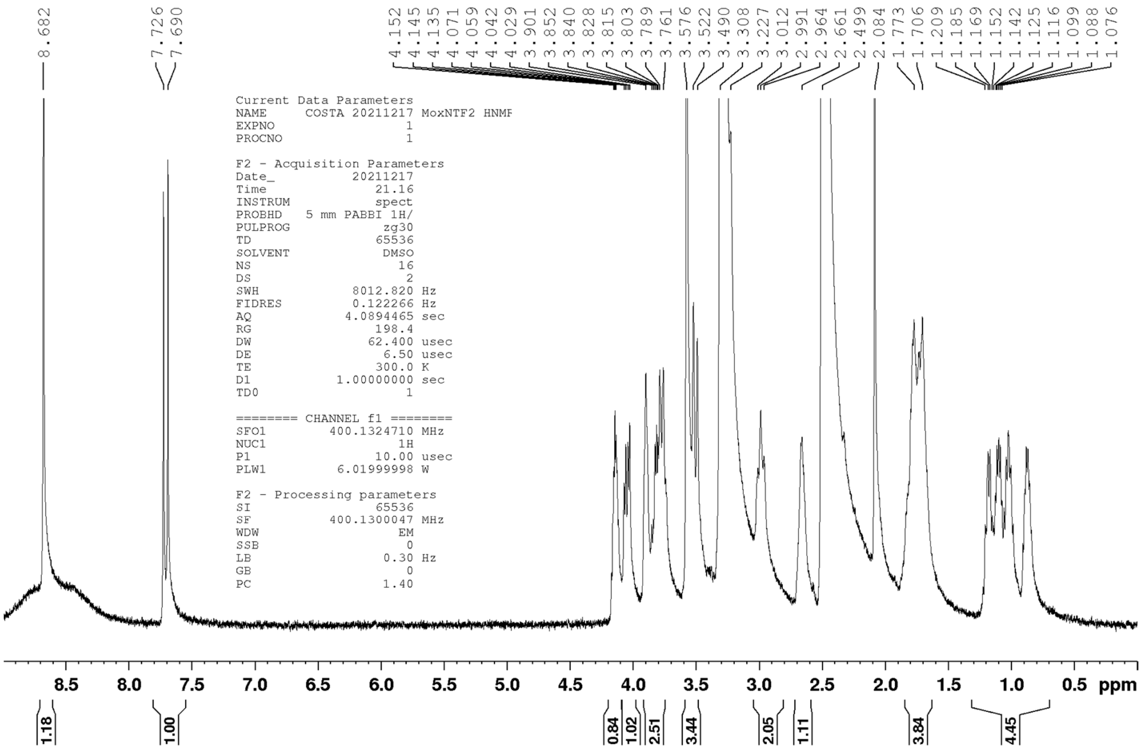
8.682
7.726
7.690
4.152
4.145
4.135
4.071
4.059
4.042
4.029
3.901
3.852
3.840
3.828
3.815
3.803
3.789
3.761
3.576
3.522
3.490
3.308
3.227
3.012
2.991
2.964
2.661
2.499
2.084
1.773
1.706
1.209
1.185
1.169
1.152
1.142
1.125
1.116
1.099
1.088
1.076
Current Data Parameters
NAME COSTA 20211217 MoxNTF2 HNMF
EXPNO 1
PROCNO 1
F2 - Acquisition Parameters
Date_ 20211217
Time 21.16
INSTRUM spect
PROBHD 5 mm PABBI 1H/
PULPROG zg30
TD 65536
SOLVENT DMSO
NS 16
DS 2
SWH 8012.820 Hz
FIDRES 0.122266 Hz
AQ 4.0894465 sec
RG 198.4
DW 62.400 usec
DE 6.50 usec
TE 300.0 K
D1 1.00000000 sec
TD0 1
======== CHANNEL f1 ========
SFO1 400.1324710 MHz
NUC1 1H
P1 10.00 usec
PLW1 6.01999998 W
F2 - Processing parameters
SI 65536
SF 400.1300047 MHz
WDW EM
SSB 0
LB 0.30 Hz
GB 0
PC 1.40
8.5
8.0
7.5
7.0
6.5
6.0
5.5
5.0
4.5
4.0
3.5
3.0
2.5
2.0
1.5
1.0
0.5 ppm
1.18
1.00
0.84
1.02
2.51
3.44
2.05
1.11
3.84
4.45

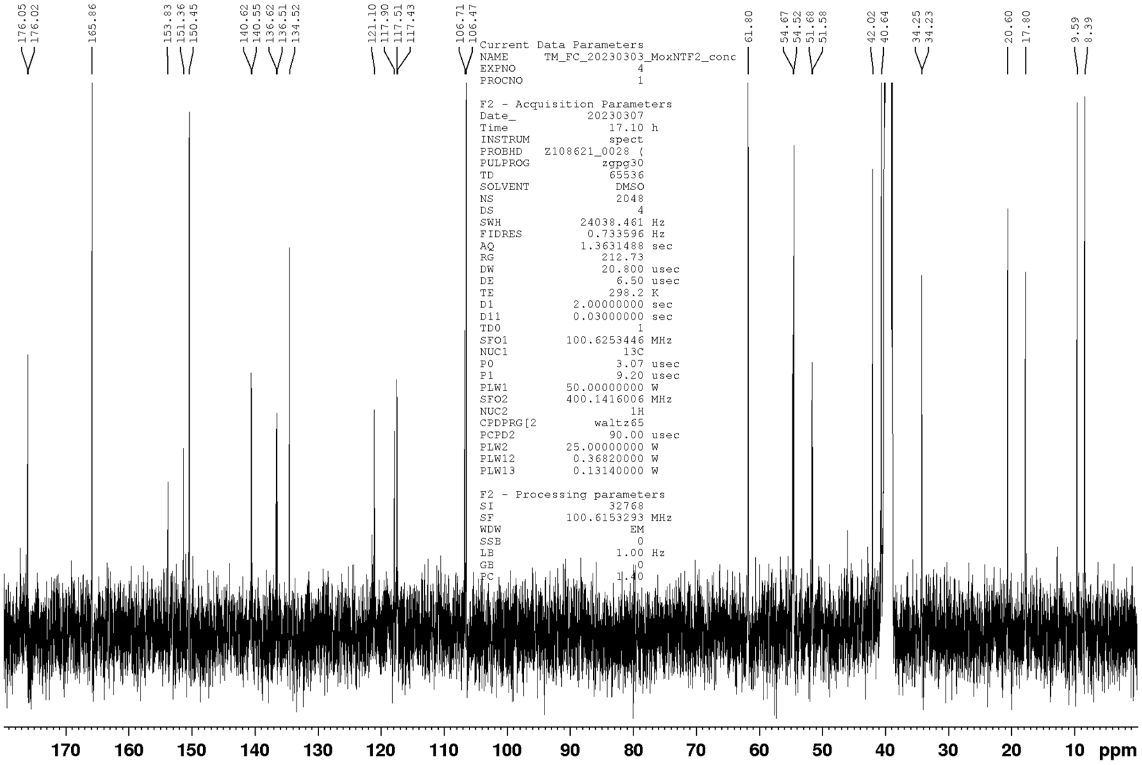
176.05
176.02
165.86
153.83
151.36
150.45
140.62
140.55
136.62
136.51
134.52
121.10
117.90
117.51
117.43
106.71
106.47
61.80
54.67
54.52
51.68
51.58
42.02
40.64
34.25
34.23
20.60
17.80
9.59
8.39
Current Data Parameters
NAME TM_FC_20230303_MoxNTF2_conc
EXPNO 4
PROCNO 1
F2 - Acquisition Parameters
Date_ 20230307
Time 17.10 h
INSTRUM spect
PROBHD Z108621_0028 (
PULPROG zgpg30
TD 65536
SOLVENT DMSO
NS 2048
DS 4
SWH 24038.461 Hz
FIDRES 0.733596 Hz
AQ 1.3631488 sec
RG 212.73
DW 20.800 usec
DE 6.50 usec
TE 298.2 K
D1 2.00000000 sec
D11 0.03000000 sec
TD0 1
SFO1 100.6253446 MHz
NUC1 13C
P0 3.07 usec
P1 9.20 usec
PLW1 50.00000000 W
SFO2 400.1416006 MHz
NUC2 1H
CPDPRG[2 waltz65
PCPD2 90.00 usec
PLW2 25.00000000 W
PLW12 0.36820000 W
PLW13 0.13140000 W
F2 - Processing parameters
SI 32768
SF 100.6153293 MHz
WDW EM
SSB 0
LB 1.00 Hz
GB 0
PC 1.40
170
160
150
140
130
120
110
100
90
80
70
60
50
40
30
20
10 ppm

Moxifloxacin tetraphenylborate [Mox][TPB]

	**Moxifloxacin [Mox]**	**Tetraphenylborate [TPB]**
**^{1}H**	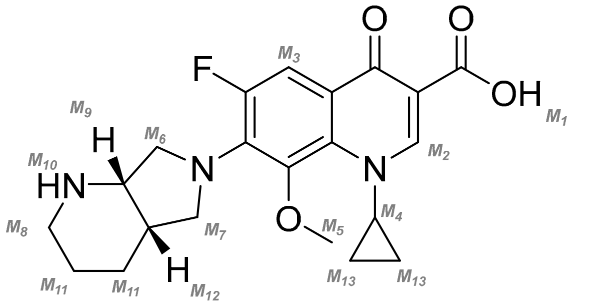	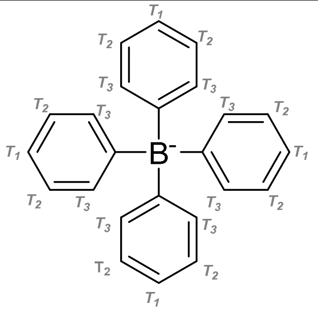
**^{13}C**	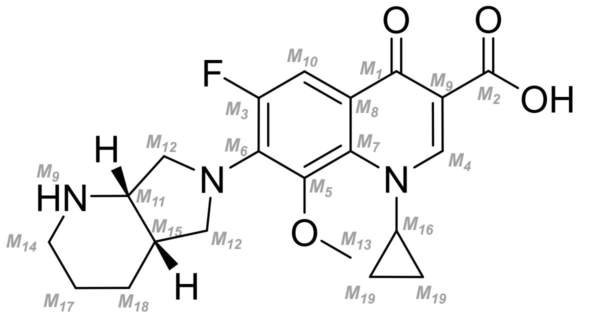	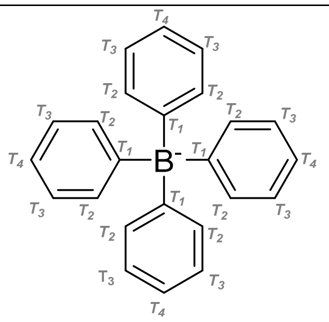

Off-yellow solid powder, yield 53%. ^{1}H NMR (400.15 MHz, DMSO-d_6, ppm), δ 15.11 (s, 1H, M1), 8.68 (s, 1H, M2), 7.71 (d, J = 14.1 Hz, 1H, M3), 7.14-7.20 (m, 8H, T3), 6.92 (t, J_1 = 7.4, J_2 = 7.4 Hz, 8H, T2), 6.83-6.74 (m, 4H, T1), 4.14 (septet, J_1 = 3.82 Hz, J_2 = 3.26 Hz, J_3 = 4.08 Hz, 1H, M4), 4.05 (dd, J_1 = 5.47 Hz, J_2 = 12.28 Hz, 1H, M6), 3.91-3.71 (m, 3H, M6-M7 and M9), 3.57 (s, 3H, M5), 3.50 (d, J = 11.98 Hz, 1H, M7), 3.23 (d, J = 12.73 Hz, 1H, M8), 3.02-2.93 (m, 1H, M8), 2.70-2.61 (m, 1H, M12), 1.85-1.67 (m, 4H, M11), 1.24-0,96 (m, 3H, M13), 0.92-0.81 (m, 1H, M13). ^{13}C NMR (100.62 MHz, DMSO-d_6, ppm) δ 176.05 (M1), 165.84 (M2), 164.10 (1C, T1), 163.61 (1C, T1), 163.12 (1C, T1), 162.63 (1C, T1), 153.82-151.34 (M3), 150.46 (M4), 140.61 (M5), 136.57 (M6), 135.54 (8C, T2), 134.51 (M7), 125.29 (dd, J_1 = 5.6, J_2 = 2.7 Hz, 8C, T3), 121.52 (4C, T4), 117.54 (M8), 106.60 (M9), 61.81 (M13), 54.50 (M11), 51.60 (M12), 41.94 (M14), 40.63 (M16), 34.17 (M15), 20.52 (M18), 17.68 (M17), 9.58-8.39 (2C, M19).

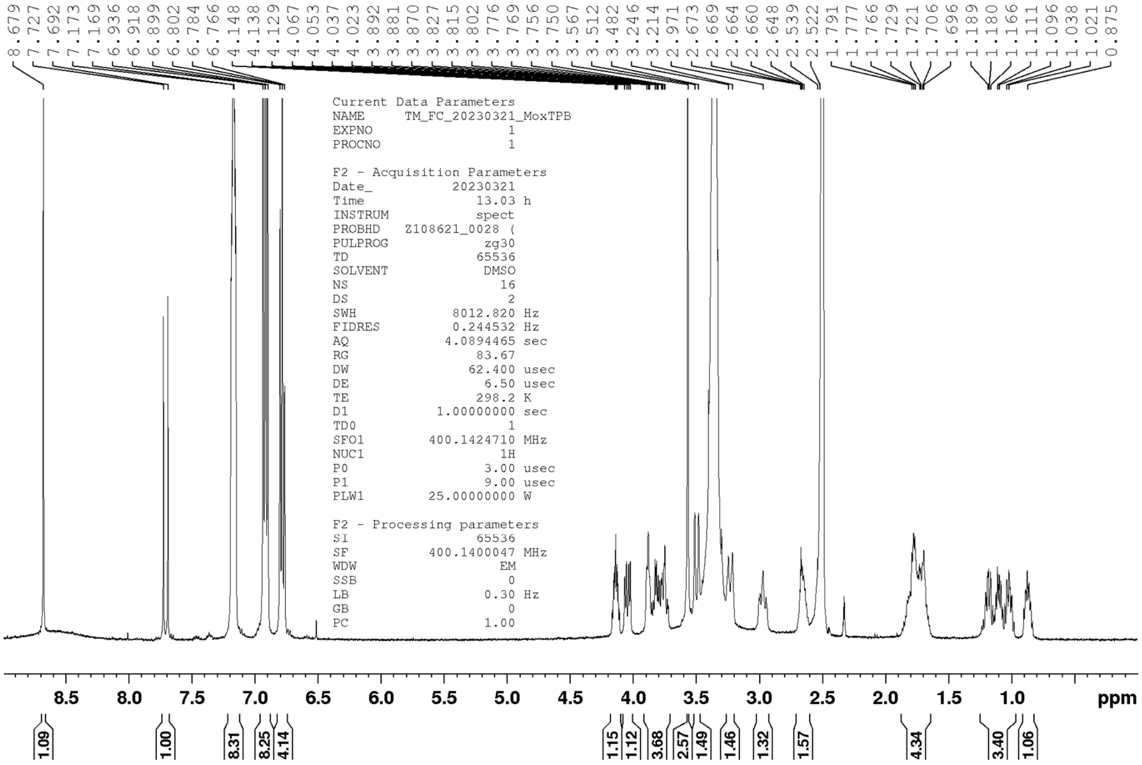
8.679 7.727 7.692 7.173 7.169 6.936 6.918 6.899 6.802 6.784 6.766 4.148 4.138 4.129 4.067 4.053 4.037 4.023 3.892 3.881 3.870 3.827 3.815 3.802 3.776 3.769 3.756 3.750 3.567 3.512 3.482 3.246 3.214 2.971 2.673 2.669 2.664 2.660 2.648 2.539 2.522 1.791 1.777 1.766 1.729 1.721 1.706 1.696 1.189 1.180 1.166 1.111 1.096 1.038 1.021 0.875
Current Data Parameters
NAME TM_FC_20230321_MoxTPB
EXPNO 1
PROCNO 1
F2 - Acquisition Parameters
Date_ 20230321
Time 13.03 h
INSTRUM spect
PROBHD Z108621_0028 (
PULPROG zg30
TD 65536
SOLVENT DMSO
NS 16
DS 2
SWH 8012.820 Hz
FIDRES 0.244532 Hz
AQ 4.0894465 sec
RG 83.67
DW 62.400 usec
DE 6.50 usec
TE 298.2 K
D1 1.00000000 sec
TD0 1
SFO1 400.1424710 MHz
NUC1 1H
P0 3.00 usec
P1 9.00 usec
PLW1 25.00000000 W
F2 - Processing parameters
SI 65536
SF 400.1400047 MHz
WDW EM
SSB 0
LB 0.30 Hz
GB 0
PC 1.00
8.5 8.0 7.5 7.0 6.5 6.0 5.5 5.0 4.5 4.0 3.5 3.0 2.5 2.0 1.5 1.0 ppm
1.09 1.00 8.31 8.25 4.14 1.15 1.12 3.68 2.57 1.49 1.46 1.32 1.57 4.34 3.40 1.06

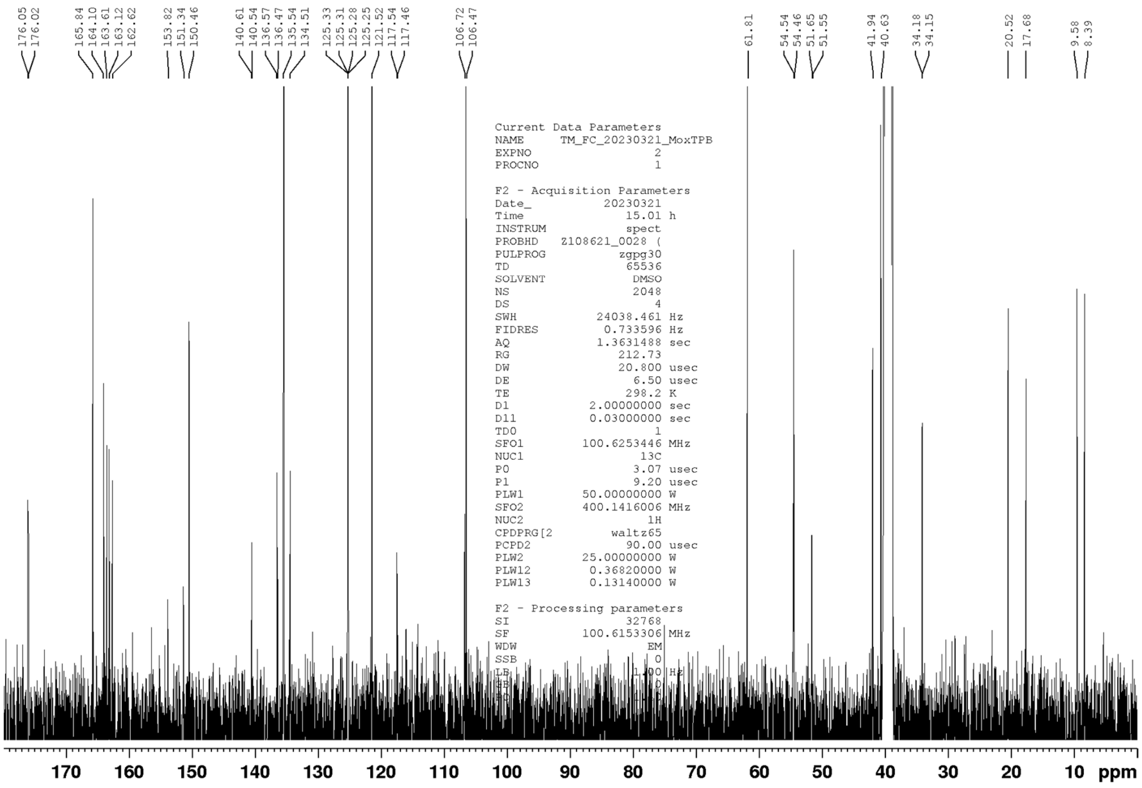
176.05 176.02 165.84 164.10 163.61 163.12 162.62 153.82 151.34 150.46 140.61 140.54 136.57 136.47 135.54 134.51 125.33 125.31 125.28 125.25 121.52 117.54 117.46 106.72 106.47 61.81 54.54 54.46 51.65 51.55 41.94 40.63 34.18 34.15 20.52 17.68 9.58 8.39
Current Data Parameters
NAME TM_FC_20230321_MoxTPB
EXPNO 2
PROCNO 1
F2 - Acquisition Parameters
Date_ 20230321
Time 15.01 h
INSTRUM spect
PROBHD Z108621_0028 (
PULPROG zgpg30
TD 65536
SOLVENT DMSO
NS 2048
DS 4
SWH 24038.461 Hz
FIDRES 0.733596 Hz
AQ 1.3631488 sec
RG 212.73
DW 20.800 usec
DE 6.50 usec
TE 298.2 K
D1 2.00000000 sec
D11 0.03000000 sec
TD0 1
SFO1 100.6253446 MHz
NUC1 13C
P0 3.07 usec
P1 9.20 usec
PLW1 50.00000000 W
SFO2 400.1416006 MHz
NUC2 1H
CPDPRG[2 waltz65
PCPD2 90.00 usec
PLW2 25.00000000 W
PLW12 0.36820000 W
PLW13 0.13140000 W
F2 - Processing parameters
SI 32768
SF 100.6153306 MHz
WDW EM
SSB 0
LB 1.00 Hz
170 160 150 140 130 120 110 100 90 80 70 60 50 40 30 20 10 ppm

Moxifloxacin docusate [Mox][Doc]

	**Moxifloxacin [Mox]**	**Docusate [Doc]**
**^{1}H**	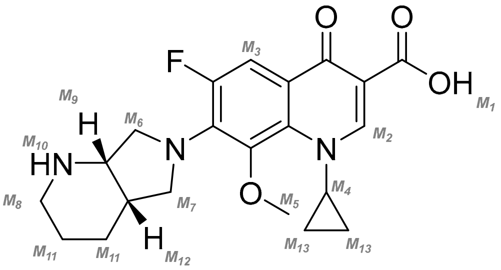	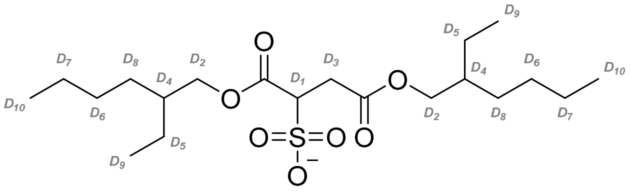
**^{13}C**	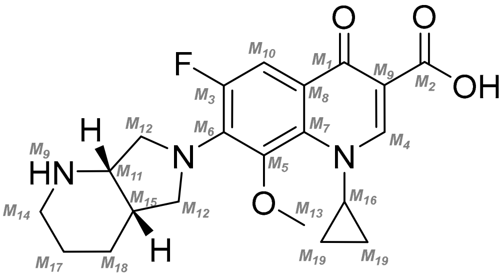	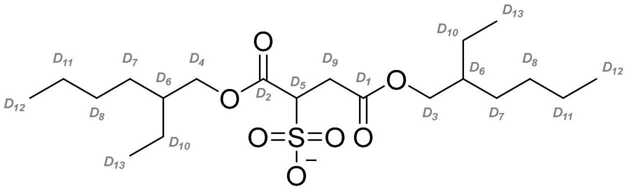

Off-yellow solid powder, yield 67%. ^{1}H NMR (400.15 MHz, DMSO-d_6, ppm), δ 15.10 (s, 1H, M1), 8.68 (s, 1H, M2), 7.71 (d, J = 14.1 Hz, 1H, M3), 4.15 (septet, J1= 3.78 Hz, J2 = 3.38 Hz, J3 = 4.00 Hz, 1H, M4), 4.05 (dd, J1= 5.61 Hz, J1= 12.06 Hz, 1H, D2), 3.94-3.73 (m, 6H, D3, M6 and M9), 3.62 (dd, J1 = 3.55 Hz, J2 = 11.48 Hz, 1H, M7), 3.58 (s, 3H, M5), 3.51 (d, J = 11.77 Hz, 1H, M7), 3.27-3.21 (m, 1H, M8), 2.90 (dd, J = 17.2, 11.5 Hz, 1H, D4), 2.78 (dd, J = 17.2, 3.7 Hz, 1H, D4), 2.71-2.63 (m, 1H, M12), 1.88-1.65 (m, 4H, M11), 1.49 (s, 2H, D5), 1.41-0.98 (m, 20H, M13 and D6-D9), 0.93-0.78 (m, 12H, D10-D11). ^{13}C NMR (100.62 MHz, DMSO-d_6, ppm), δ 176.01 (M1), 171.01 (D1), 168.31 (M2), 165.77 (D2), 153.76-151.28 (M3), 150.41 (M4), 140.56-140.49 (M5), 136.53-136.43 (M6), 134.47 (M7), 117.51-117.42 (M8), 106.68 (M10), 106.45 (M9), 66.15-66.07 (D3), 61.79 (M13), 61.41 (D4), 54.39 (M12), 54.23 (M11), 51.63-51.54 (M12), 41.85 (M14), 40.59 (M16), 38.15 (D6), 34.09 (M15), 29.71-28.31 (D9), 23.17-22.97 (D10), 22.38-22.37 (D11), 20.45 (M18), 17.58 (M17), 13.89-13.86 (D12), 10.79-10.72 (D13), 9.55-8.36 (2C, M19).

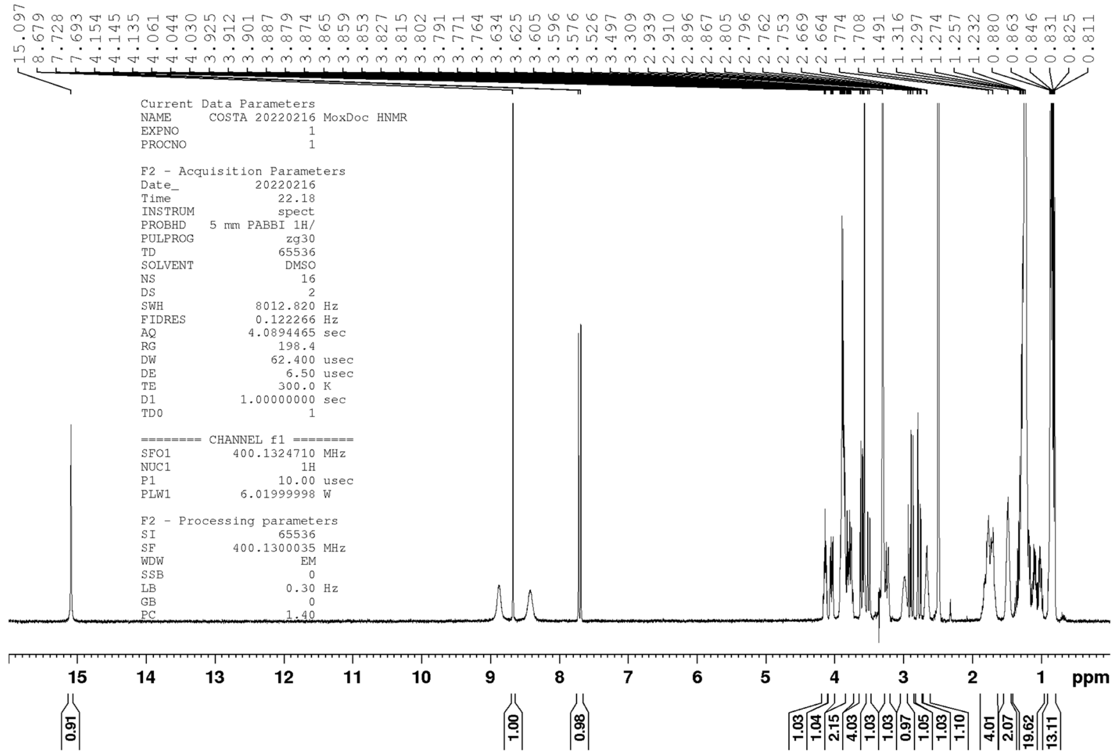
15.097 8.679 7.728 7.693 4.154 4.145 4.135 4.061 4.044 4.030 3.925 3.912 3.901 3.887 3.879 3.874 3.865 3.859 3.853 3.827 3.815 3.802 3.791 3.771 3.764 3.634 3.625 3.605 3.596 3.576 3.526 3.497 3.309 2.939 2.910 2.896 2.867 2.805 2.796 2.762 2.753 2.669 2.664 1.774 1.708 1.491 1.316 1.297 1.274 1.257 1.232 0.880 0.863 0.846 0.831 0.825 0.811
Current Data Parameters
NAME COSTA 20220216 MoxDoc HNMR
EXPNO 1
PROCNO 1
F2 - Acquisition Parameters
Date_ 20220216
Time 22.18
INSTRUM spect
PROBHD 5 mm PABBI 1H/
PULPROG zg30
TD 65536
SOLVENT DMSO
NS 16
DS 2
SWH 8012.820 Hz
FIDRES 0.122266 Hz
AQ 4.0894465 sec
RG 198.4
DW 62.400 usec
DE 6.50 usec
TE 300.0 K
D1 1.00000000 sec
TD0 1
======== CHANNEL f1 ========
SFO1 400.1324710 MHz
NUC1 1H
P1 10.00 usec
PLW1 6.01999998 W
F2 - Processing parameters
SI 65536
SF 400.1300035 MHz
WDW EM
SSB 0
LB 0.30 Hz
GB 0
PC 1.40
15 14 13 12 11 10 9 8 7 6 5 4 3 2 1 ppm
0.91 1.00 0.98 1.03 1.04 2.15 4.03 1.03 1.03 0.97 1.05 1.03 1.10 4.01 2.07 19.62 13.11

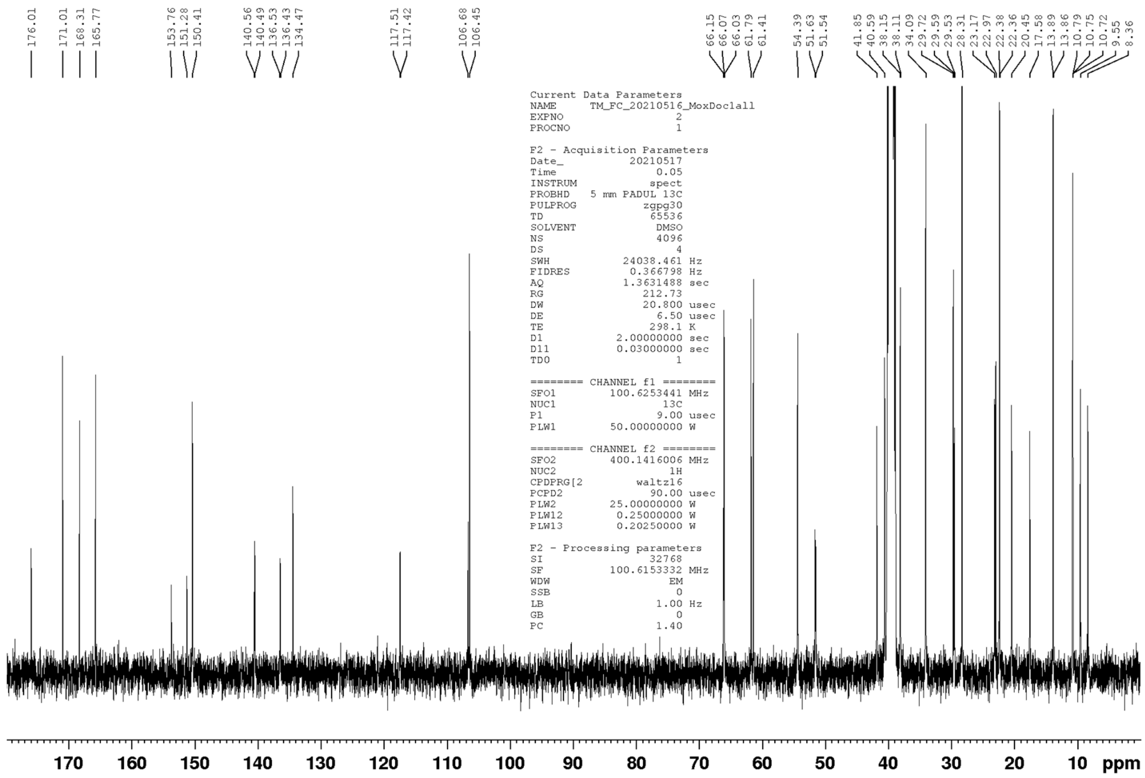
176.01 171.01 168.31 165.77 153.76 151.28 150.41 140.56 140.49 136.53 136.43 134.47 117.51 117.42 106.68 106.45 66.15 66.07 66.03 61.79 61.41 54.39 51.63 51.54 41.85 40.59 38.15 38.11 34.09 29.72 29.59 29.53 28.31 23.17 22.97 22.38 22.36 20.45 17.58 13.89 13.86 10.79 10.75 10.72 9.55 8.36
Current Data Parameters
NAME TM_FC_20210516_MoxDoclall
EXPNO 2
PROCNO 1
F2 - Acquisition Parameters
Date_ 20210517
Time 0.05
INSTRUM spect
PROBHD 5 mm PADUL 13C
PULPROG zgpg30
TD 65536
SOLVENT DMSO
NS 4096
DS 4
SWH 24038.461 Hz
FIDRES 0.366798 Hz
AQ 1.3631488 sec
RG 212.73
DW 20.800 usec
DE 6.50 usec
TE 298.1 K
D1 2.00000000 sec
D11 0.03000000 sec
TD0 1
======== CHANNEL f1 ========
SFO1 100.6253441 MHz
NUC1 13C
P1 9.00 usec
PLW1 50.00000000 W
======== CHANNEL f2 ========
SFO2 400.1416006 MHz
NUC2 1H
CPDPRG[2 waltz16
PCPD2 90.00 usec
PLW2 25.00000000 W
PLW12 0.25000000 W
PLW13 0.20250000 W
F2 - Processing parameters
SI 32768
SF 100.6153332 MHz
WDW EM
SSB 0
LB 1.00 Hz
GB 0
PC 1.40
170 160 150 140 130 120 110 100 90 80 70 60 50 40 30 20 10 ppm

Moxifloxacin deoxycholate [Mox][Dxc]

	**Moxifloxacin [Mox]**	**Deoxycholate [Dxc]**
**^{1}H**	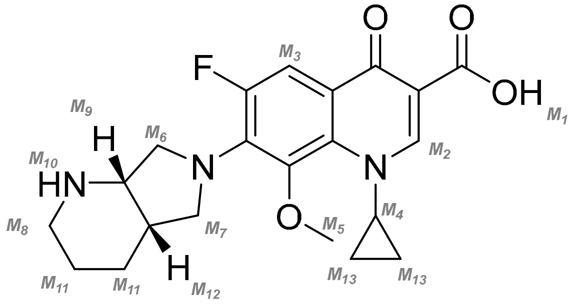	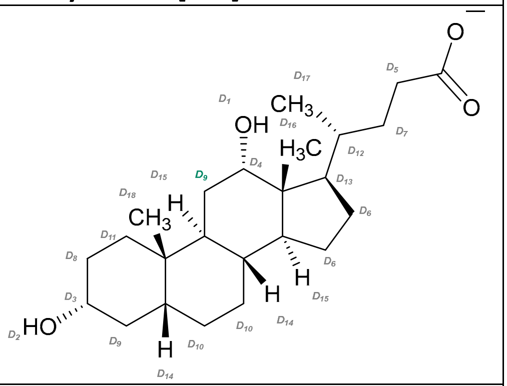
**^{13}C**	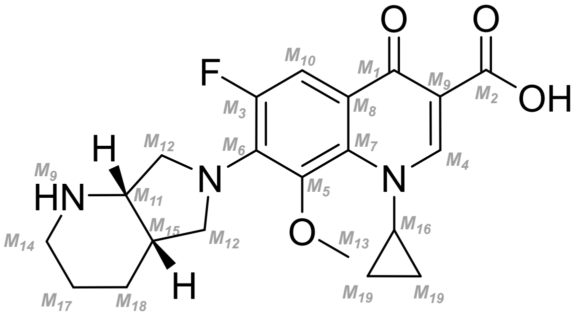	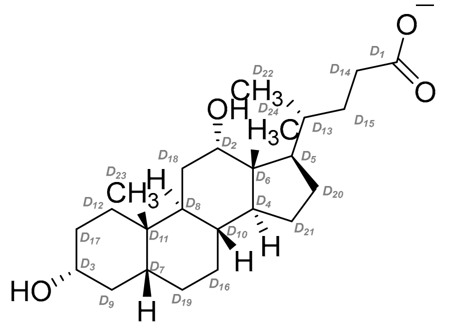

Off-yellow solid powder, yield 48%. ^{1}H NMR (400.15 MHz, DMSO-d_6, ppm), δ 8.64 (s, 1H, M2), 7.64 (d, J = 14.28 Hz, 1H, M3), 4.44 (m, 1H, D1), 4.19 (m, 1H, D2), 4.12 (septet, J_1 = 3.68 Hz, J_2 = 3.44 Hz, J_3 = 4.08 Hz, 1H, M4), 4.00-3.94 (m, 1H, M6), 3.90-3.86 (m, 1H, M9), 3.78 (s, 2H, M6-M7), 3.55 (s, 3H, M5), 3.24 (m, 2H, M8), 2.89-2.85 (m, 1H, M12), 2.27-2.17 (m, 4H, D6), 2.13-2.04 (m, 2H, D5), 1.84-0.95 (m, 24H, M10-11, M13 and D7-15), 0.91 (d, J_1 = 6,43 Hz, 3H, D16), 0.86-0.82 (m, 3H, D17), 0.59 (s, 3H, D18). ^{13}C NMR (100.62 MHz, DMSO-d_6, ppm), δ 176.32 (M1), 175.02 (D1), 165.85 (M2), 153.98 (M3), 150.09 (M4), 140.30 (M5), 137.43 (M6), 134.69 (M7), 116.53 (M8), 106.44 (M10), 106.22 (M9), 70.99 (D2), 69.92 (D3), 61.21 (M13), 58.62 (M12), 56.05 (M11), 52.42 (M12), 47.46 (D4), 46.17 (D5), 45.98 (D6), 44.53 (M14), 41.60 (D7), 40.73 (M16), 36.29 (2C, D8-D9), 35.63 (D10), 35.13 (D11), 34.96 (D12), 33.82 (2C, D13-D14), 32.91 (D15), 30.91 (M15), 30.24 (D16), 28.58 (D17), 27.17 (D18), 26.97 (D19), 26.09 (D20), 23.49 (D21), 23.08 (D22), 22.97 (M18), 21.51 (D23), 16.91 (M17), 12.43 (D24), 9.72-8.17 (M19).

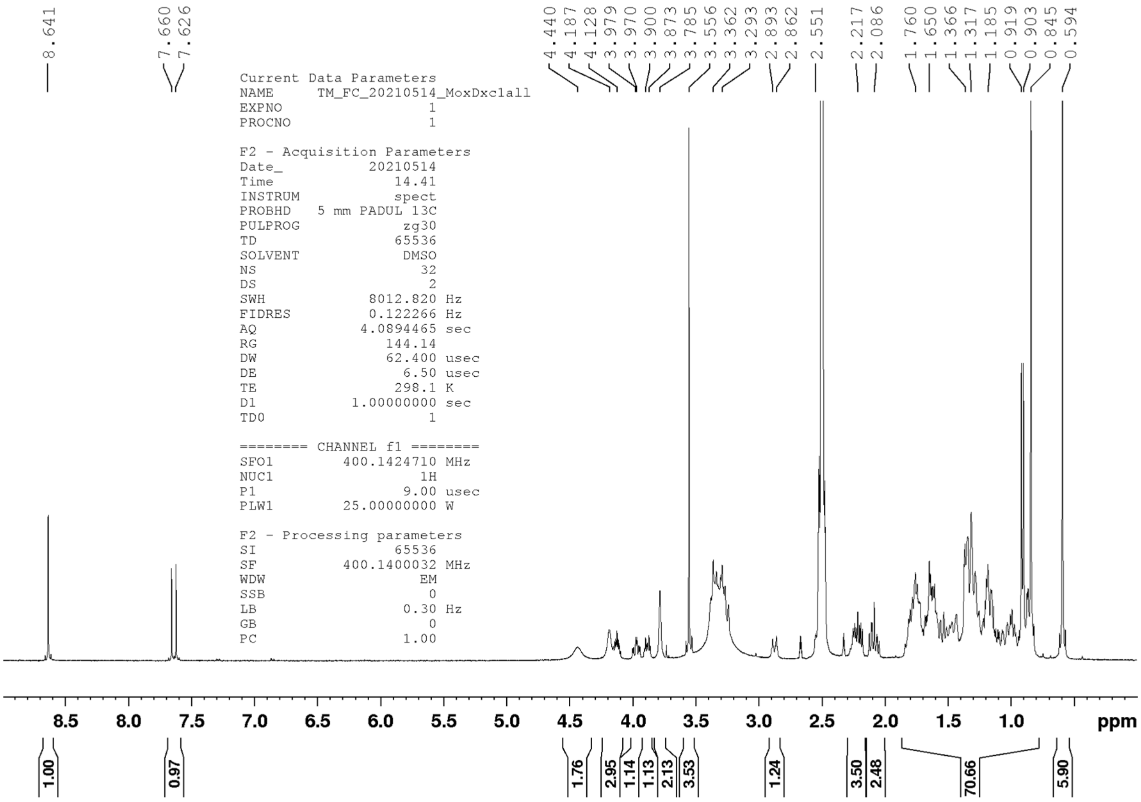
8.641
7.660
7.626
4.440 4.187 4.128 3.979 3.970 3.900 3.873 3.785 3.556 3.362 3.293 2.893 2.862 2.551 2.217 2.086 1.760 1.650 1.366 1.317 1.185 0.919 0.903 0.845 0.594
Current Data Parameters
NAME TM_FC_20210514_MoxDxcl1all
EXPNO 1
PROCNO 1
F2 - Acquisition Parameters
Date_ 20210514
Time 14.41
INSTRUM spect
PROBHD 5 mm PADUL 13C
PULPROG zg30
TD 65536
SOLVENT DMSO
NS 32
DS 2
SWH 8012.820 Hz
FIDRES 0.122266 Hz
AQ 4.0894465 sec
RG 144.14
DW 62.400 usec
DE 6.50 usec
TE 298.1 K
D1 1.00000000 sec
TD0 1
======== CHANNEL f1 ========
SFO1 400.1424710 MHz
NUC1 1H
P1 9.00 usec
PLW1 25.00000000 W
F2 - Processing parameters
SI 65536
SF 400.1400032 MHz
WDW EM
SSB 0
LB 0.30 Hz
GB 0
PC 1.00
8.5 8.0 7.5 7.0 6.5 6.0 5.5 5.0 4.5 4.0 3.5 3.0 2.5 2.0 1.5 1.0 ppm
1.00 0.97 1.76 2.95 1.14 1.13 2.13 3.53 1.24 3.50 2.48 70.66 5.90

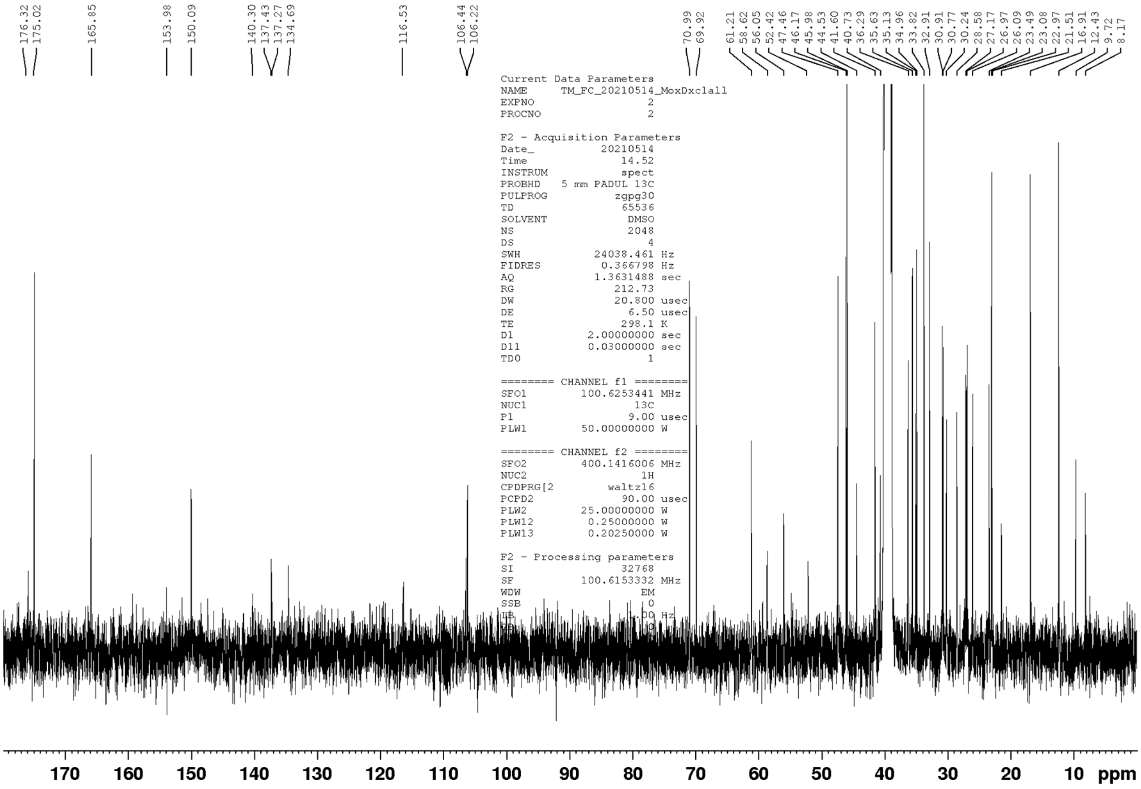
176.32 175.02 165.85 153.98 150.09 140.30 137.43 137.27 134.69 116.53 106.44 106.22
70.99 69.92 61.21 58.62 56.05 52.42 47.46 46.17 45.98 44.53 41.60 40.73 36.29 35.63 35.13 34.96 33.82 32.91 30.91 30.77 30.24 28.58 27.17 26.97 26.09 23.49 23.08 22.97 21.51 16.91 12.43 9.72 8.17
Current Data Parameters
NAME TM_FC_20210514_MoxDxcl1all
EXPNO 2
PROCNO 2
F2 - Acquisition Parameters
Date_ 20210514
Time 14.52
INSTRUM spect
PROBHD 5 mm PADUL 13C
PULPROG zgpg30
TD 65536
SOLVENT DMSO
NS 2048
DS 4
SWH 24038.461 Hz
FIDRES 0.366798 Hz
AQ 1.3631488 sec
RG 212.73
DW 20.800 usec
DE 6.50 usec
TE 298.1 K
D1 2.00000000 sec
D11 0.03000000 sec
TD0 1
======== CHANNEL f1 ========
SFO1 100.6253441 MHz
NUC1 13C
P1 9.00 usec
PLW1 50.00000000 W
======== CHANNEL f2 ========
SFO2 400.1416006 MHz
NUC2 1H
CPDPRG[2 waltz16
PCPD2 90.00 usec
PLW2 25.00000000 W
PLW12 0.25000000 W
PLW13 0.20250000 W
F2 - Processing parameters
SI 32768
SF 100.6153332 MHz
WDW EM
SSB 0
170 160 150 140 130 120 110 100 90 80 70 60 50 40 30 20 10 ppm

DSC thermograms

Parent compounds

[Cip][HCl]

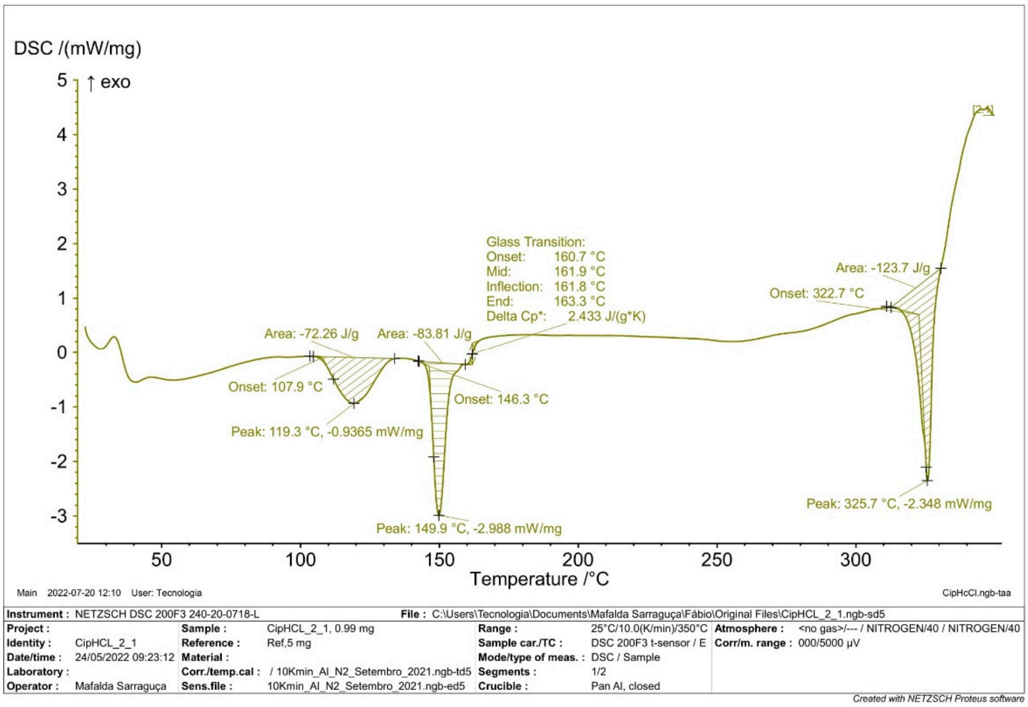

[Mox][HCl]

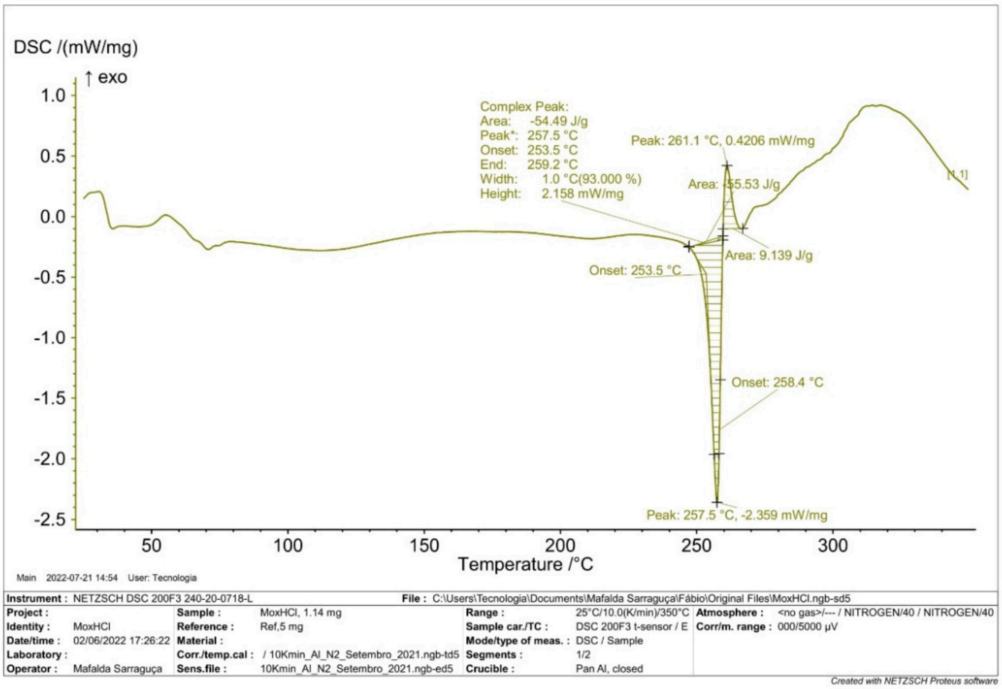

[Li][BETI]

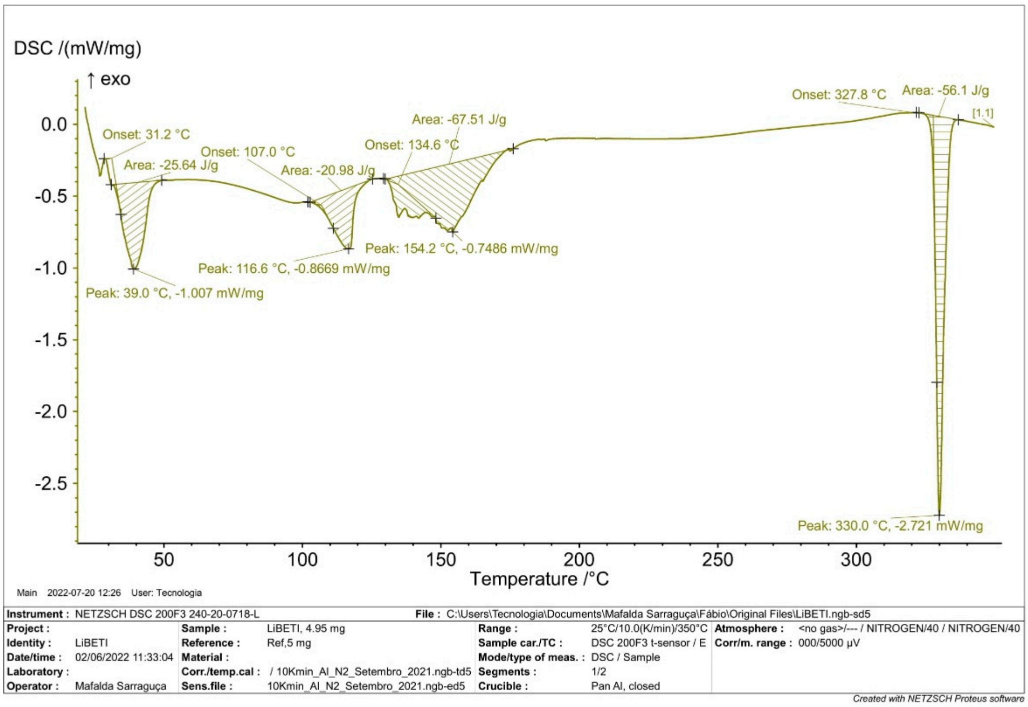

[Li][NTF₂]

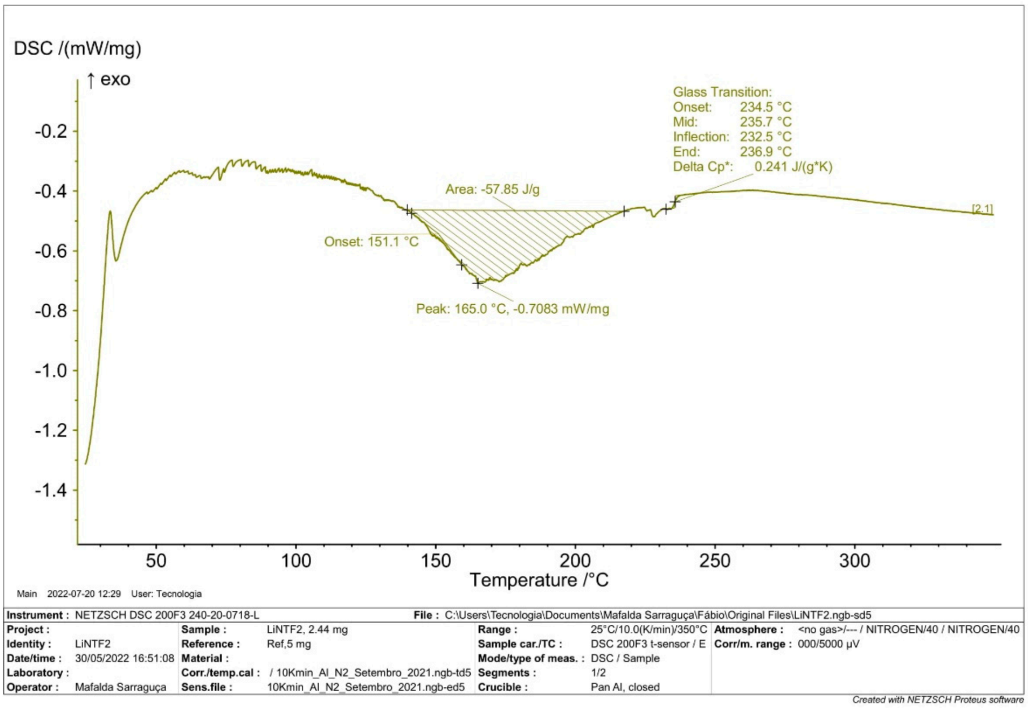

[Na][TPB]

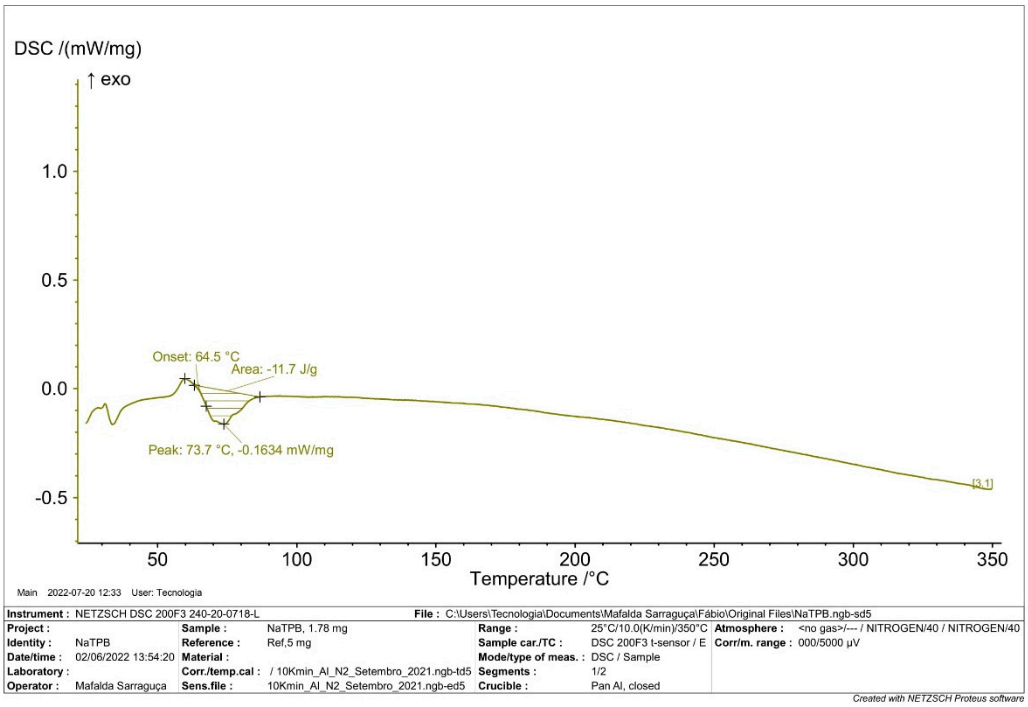
DSC /(mW/mg)
↑ exo
1.0
0.5
0.0
-0.5
Onset: 64.5 °C
Area: -11.7 J/g
Peak: 73.7 °C, -0.1634 mW/mg
[3.1]
50
100
150
200
250
300
350
Temperature /°C
Main 2022-07-20 12:33 User: Tecnologia
Instrument : NETZSCH DSC 200F3 240-20-0718-L
File : C:\Users\Tecnologia\Documents\Mafalda Sarraguça\Fábio\Original Files\NaTPB.ngb-sd5
Project :
Identity : NaTPB
Date/time : 02/06/2022 13:54:20
Laboratory :
Operator : Mafalda Sarraguça
Sample : NaTPB, 1.78 mg
Reference : Ref,5 mg
Material :
Corr./temp.cal : / 10Kmin_Al_N2_Setembro_2021.ngb-td5
Sens.file : 10Kmin_Al_N2_Setembro_2021.ngb-ed5
Range : 25°C/10.0(K/min)/350°C
Sample car./TC : DSC 200F3 t-sensor / E
Mode/type of meas. : DSC / Sample
Segments : 1/2
Crucible : Pan Al, closed
Atmosphere : <no gas>/--- / NITROGEN/40 / NITROGEN/40
Corr/m. range : 000/5000 µV
Created with NETZSCH Proteus software

[Na][Doc]

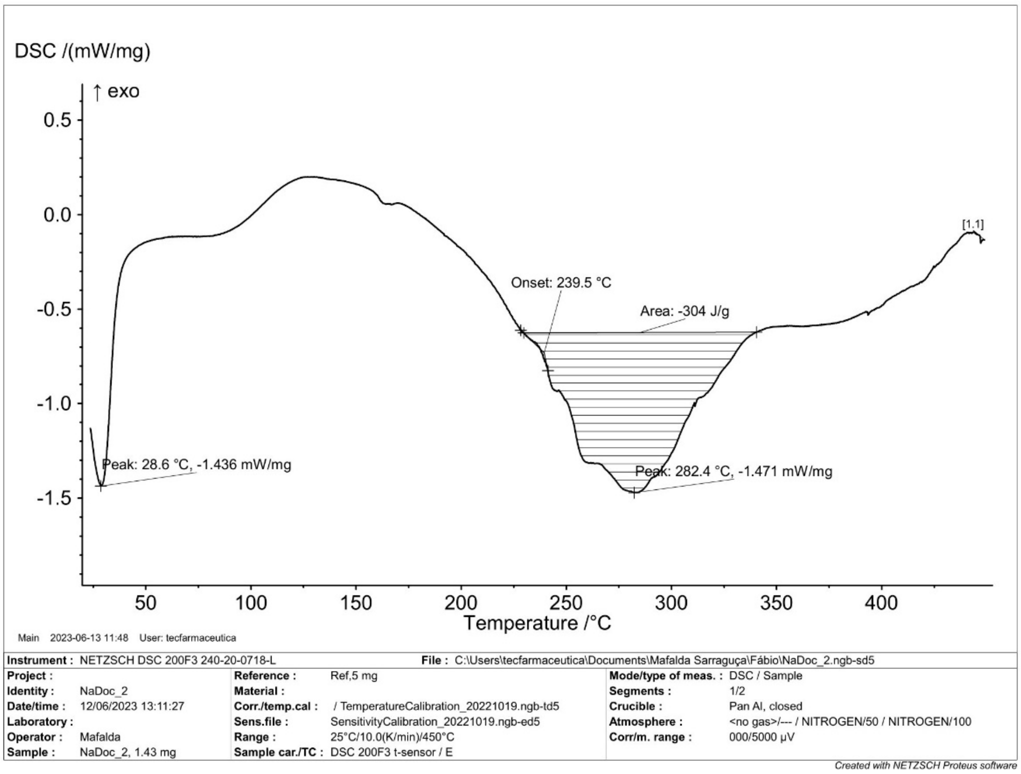
DSC /(mW/mg)
↑ exo
0.5
0.0
-0.5
-1.0
-1.5
Onset: 239.5 °C
Area: -304 J/g
Peak: 28.6 °C, -1.436 mW/mg
Peak: 282.4 °C, -1.471 mW/mg
[1.1]
50
100
150
200
250
300
350
400
Temperature /°C
Main 2023-06-13 11:48 User: tecfarmaceutica
Instrument : NETZSCH DSC 200F3 240-20-0718-L
File : C:\Users\tecfarmaceutica\Documents\Mafalda Sarraguça\Fábio\NaDoc_2.ngb-sd5
Project :
Identity : NaDoc_2
Date/time : 12/06/2023 13:11:27
Laboratory :
Operator : Mafalda
Sample : NaDoc_2, 1.43 mg
Reference : Ref,5 mg
Material :
Corr./temp.cal : / TemperatureCalibration_20221019.ngb-td5
Sens.file : SensitivityCalibration_20221019.ngb-ed5
Range : 25°C/10.0(K/min)/450°C
Sample car./TC : DSC 200F3 t-sensor / E
Mode/type of meas. : DSC / Sample
Segments : 1/2
Crucible : Pan Al, closed
Atmosphere : <no gas>/--- / NITROGEN/50 / NITROGEN/100
Corr/m. range : 000/5000 µV
Created with NETZSCH Proteus software

[Na][Dxc]

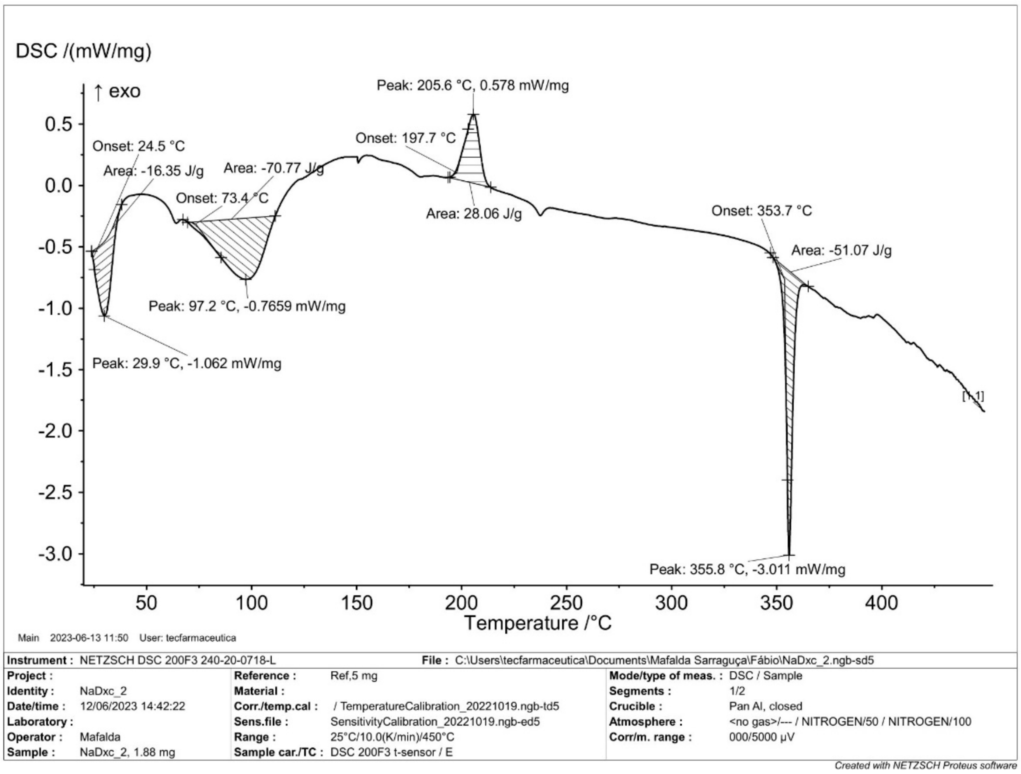
DSC /(mW/mg)
↑ exo
Peak: 205.6 °C, 0.578 mW/mg
Onset: 197.7 °C
Area: 28.06 J/g
Onset: 24.5 °C
Area: -16.35 J/g
Area: -70.77 J/g
Onset: 73.4 °C
Peak: 97.2 °C, -0.7659 mW/mg
Peak: 29.9 °C, -1.062 mW/mg
Onset: 353.7 °C
Area: -51.07 J/g
Peak: 355.8 °C, -3.011 mW/mg
[1.1]
0.5
0.0
-0.5
-1.0
-1.5
-2.0
-2.5
-3.0
50
100
150
200
250
300
350
400
Temperature /°C
Main 2023-06-13 11:50 User: tecfarmaceutica
Instrument : NETZSCH DSC 200F3 240-20-0718-L
File : C:\Users\tecfarmaceutica\Documents\Mafalda Sarraguça\Fábio\NaDxc_2.ngb-sd5
Project :
Identity : NaDxc_2
Date/time : 12/06/2023 14:42:22
Laboratory :
Operator : Mafalda
Sample : NaDxc_2, 1.88 mg
Reference : Ref,5 mg
Material :
Corr./temp.cal : / TemperatureCalibration_20221019.ngb-td5
Sens.file : SensitivityCalibration_20221019.ngb-ed5
Range : 25°C/10.0(K/min)/450°C
Sample car./TC : DSC 200F3 t-sensor / E
Mode/type of meas. : DSC / Sample
Segments : 1/2
Crucible : Pan Al, closed
Atmosphere : <no gas>/--- / NITROGEN/50 / NITROGEN/100
Corr/m. range : 000/5000 µV
Created with NETZSCH Proteus software

GUMBOS

[Cip][BETI]

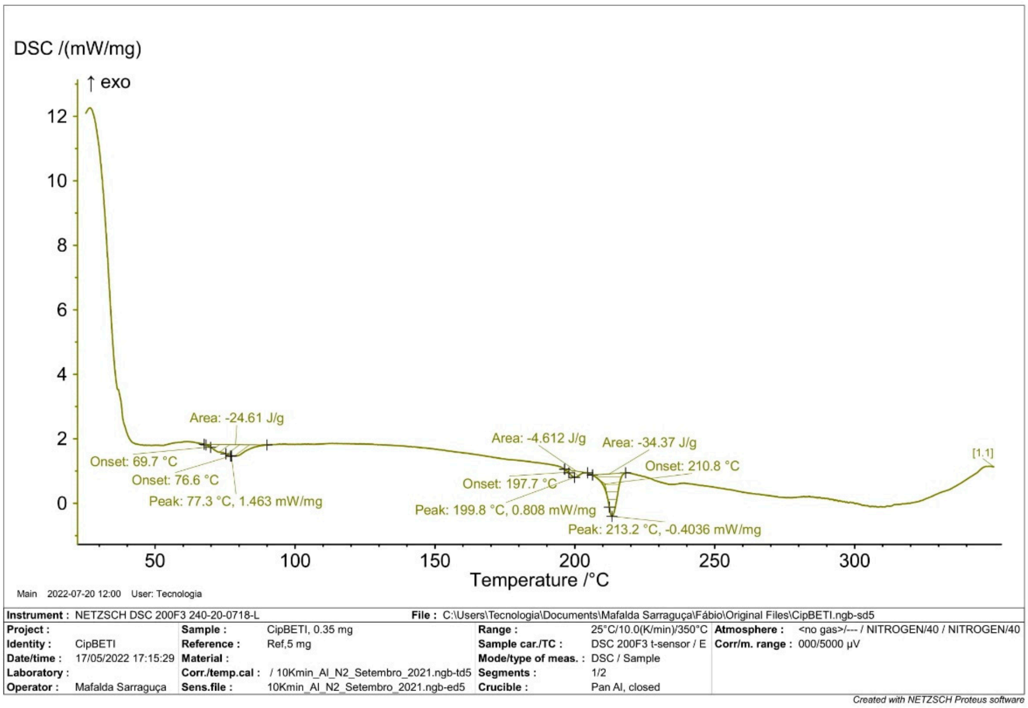

[Cip][NTF$_2$]

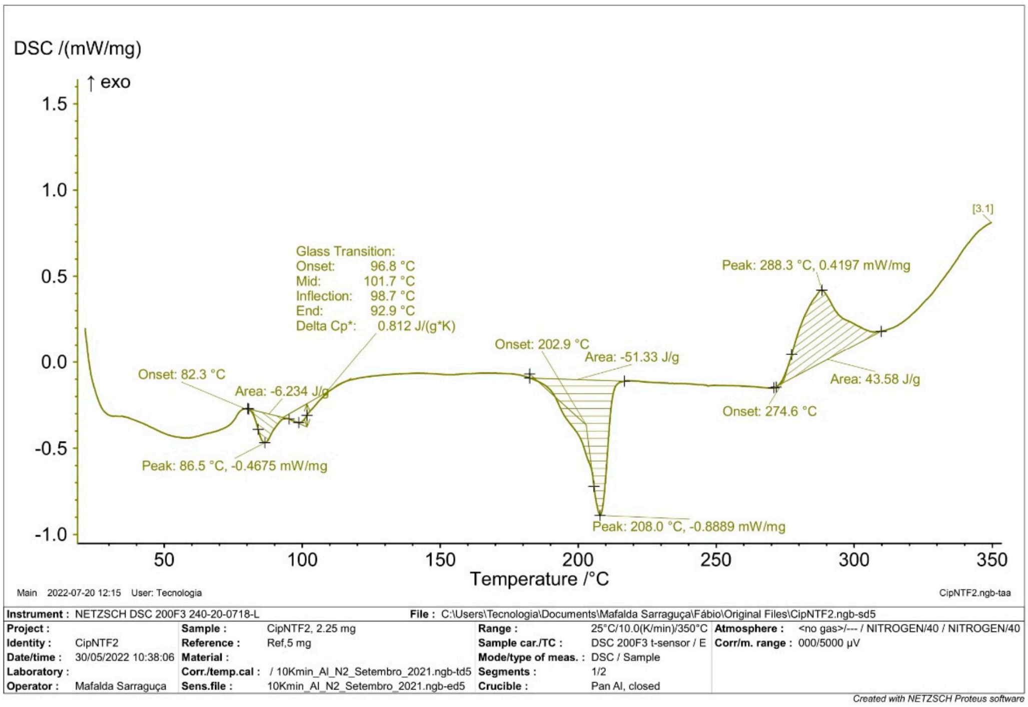

[Cip][TPB]

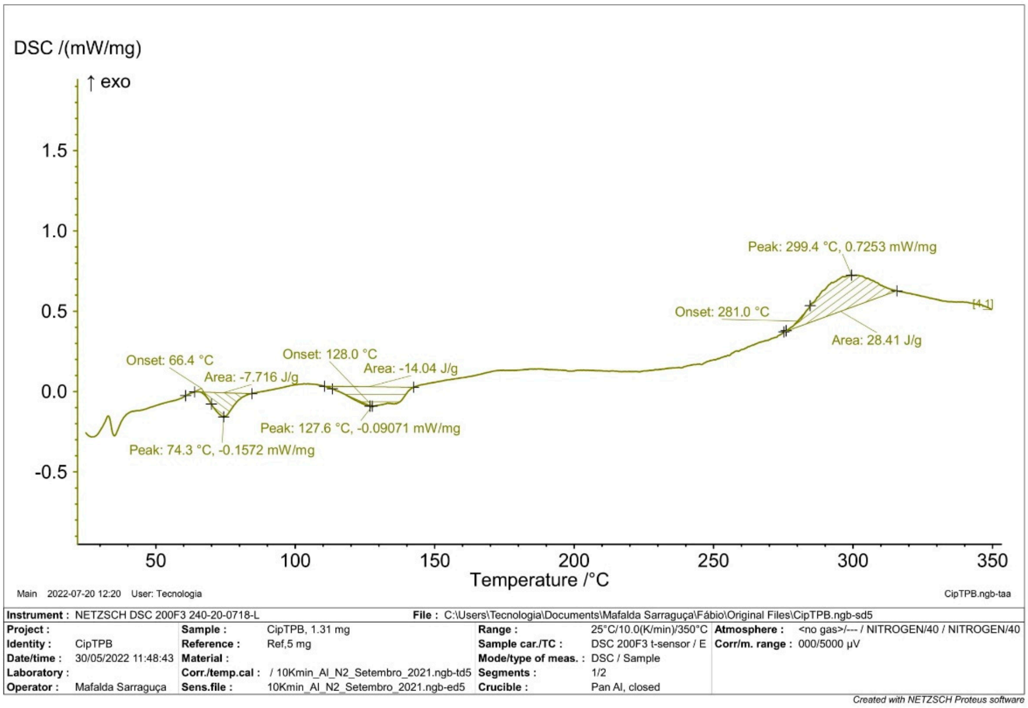

[Cip][Doc]

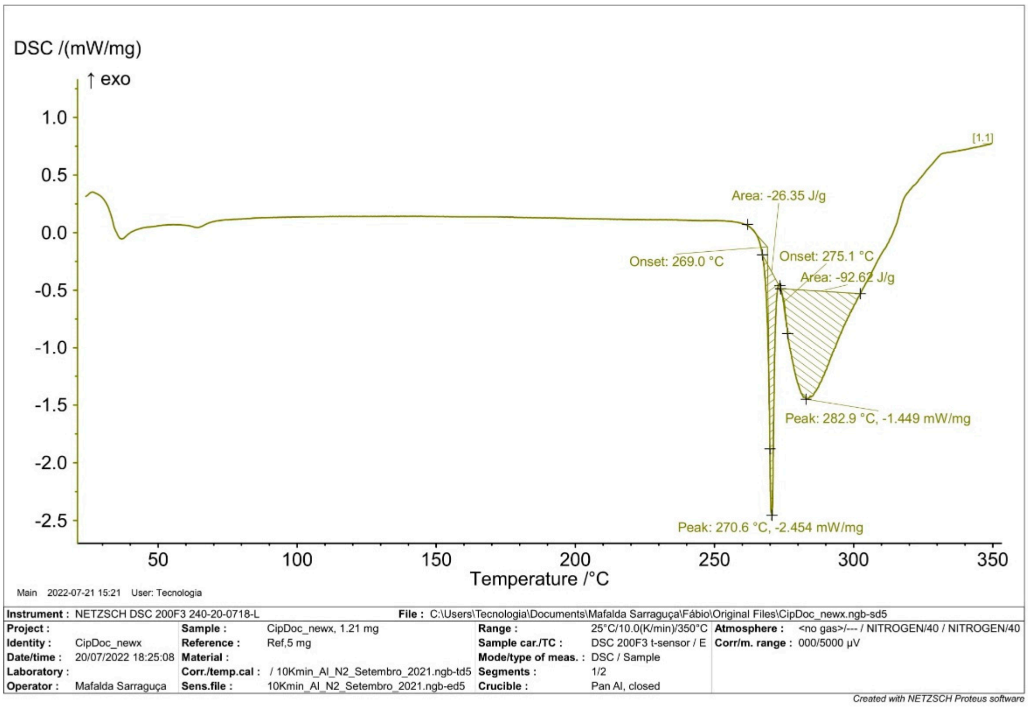

[Cip][Dxc]

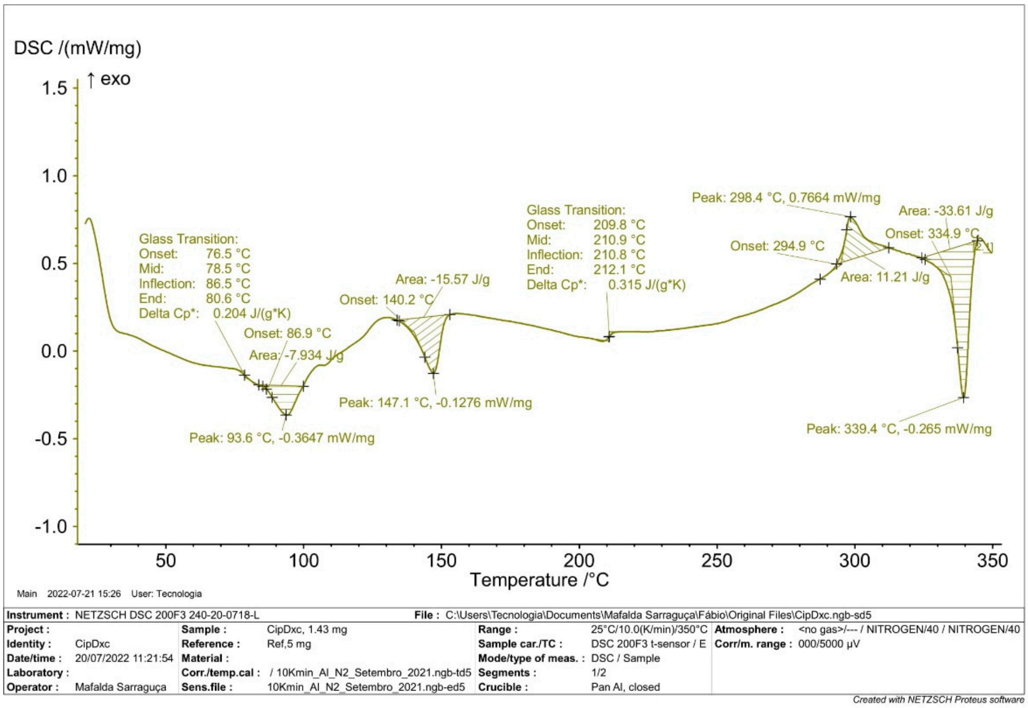

[Mox][BETI]

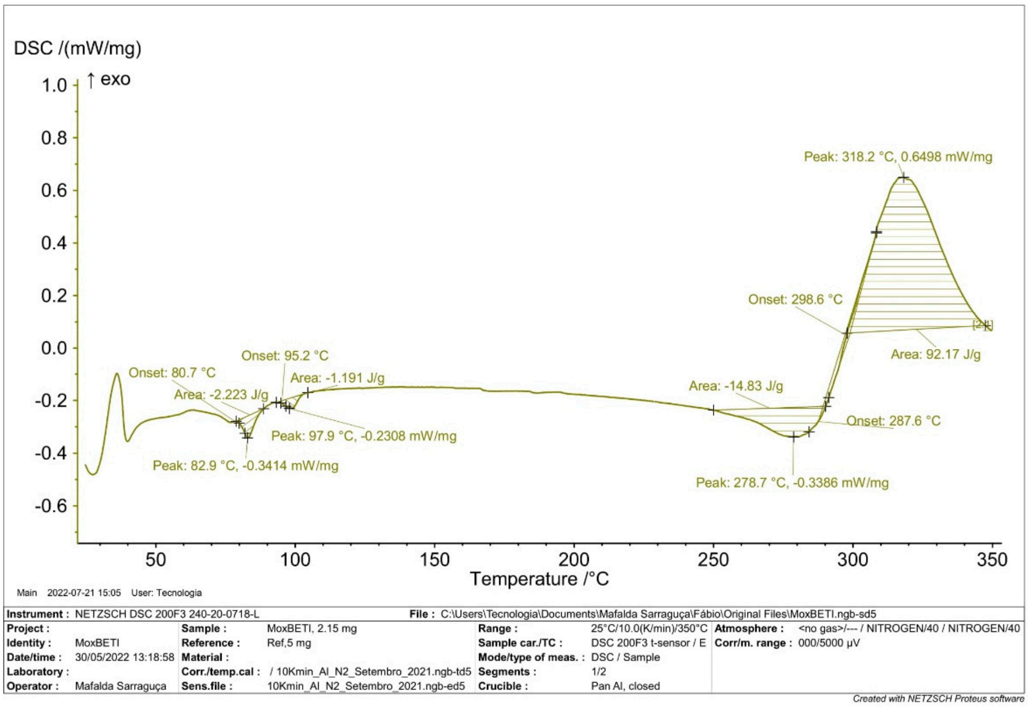

[Mox][NTF$_2$]

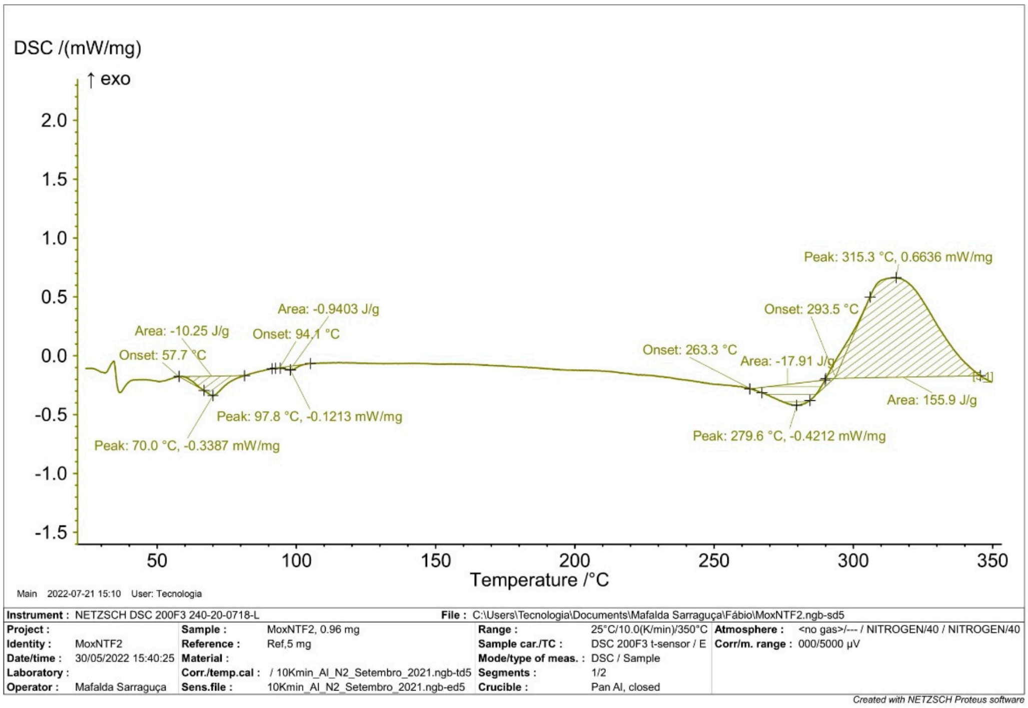

[Mox][TPB]

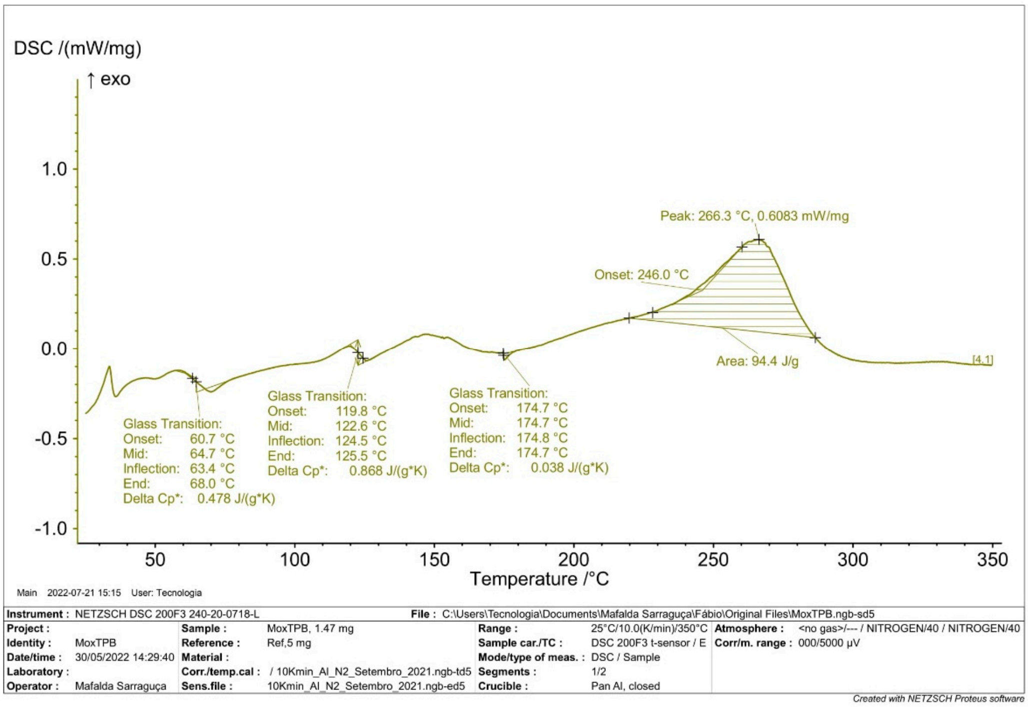

[Mox][Doc]

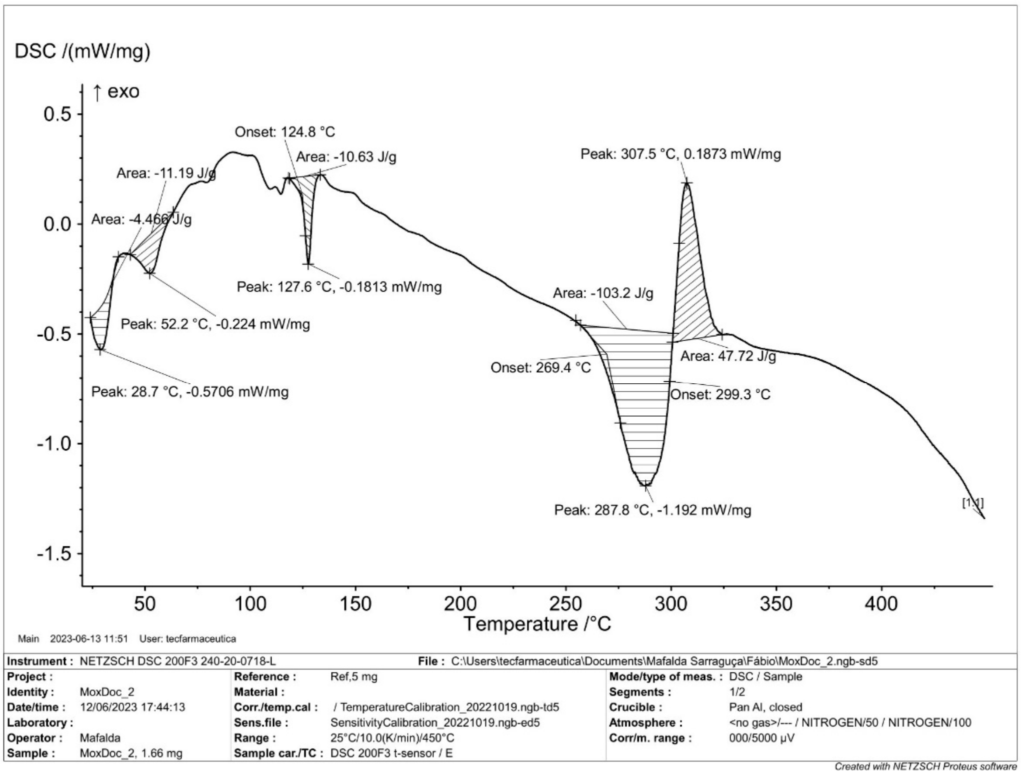

[Mox][Dxc]

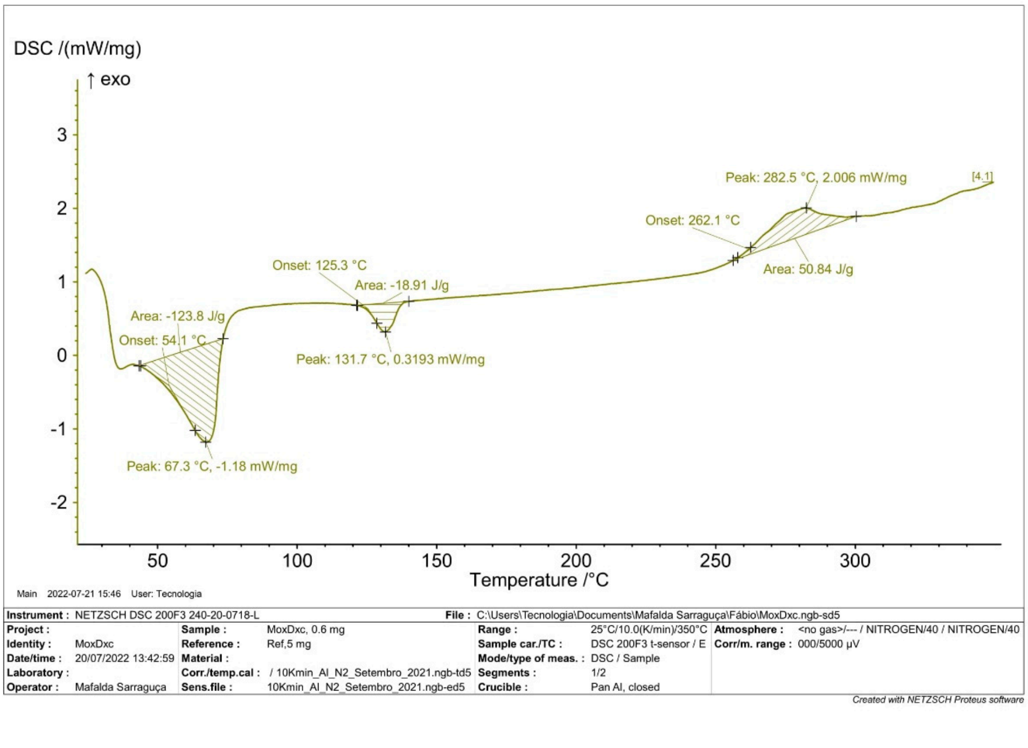

TGA analysis

GUMBOS

[Cip][BETI]

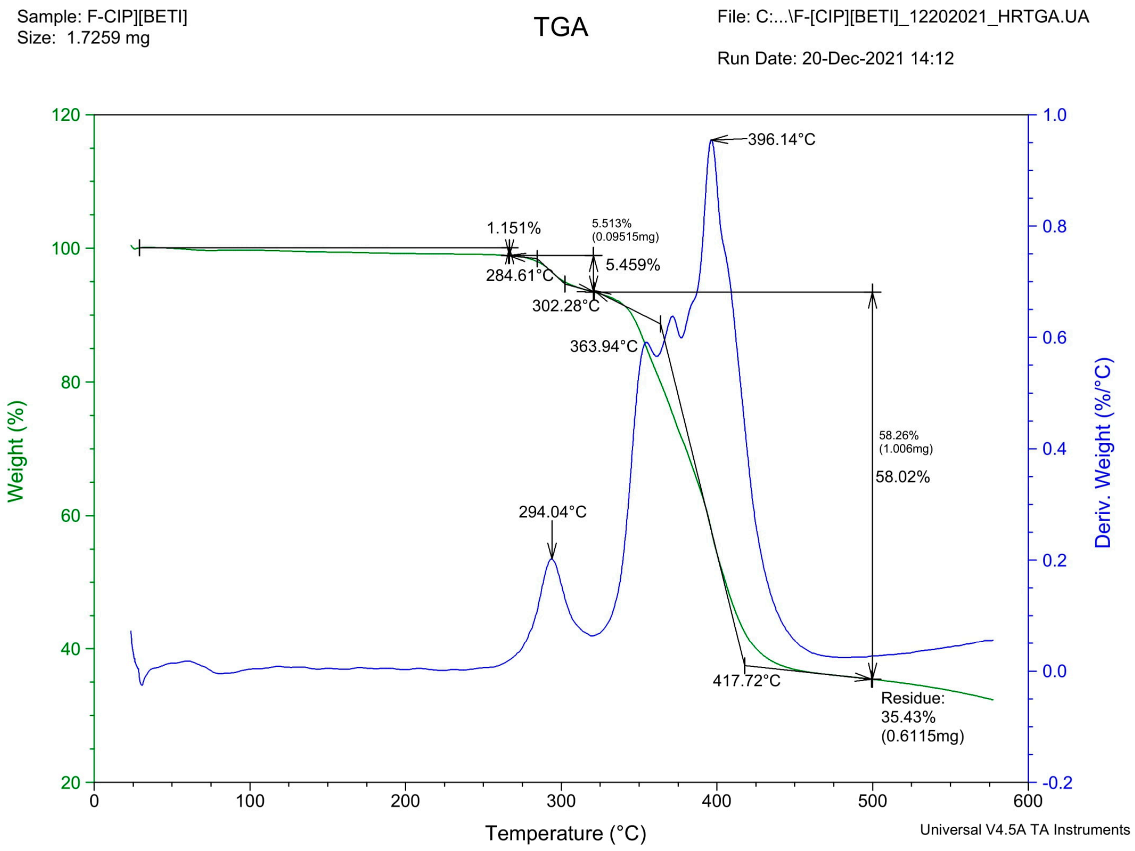

[Cip][NTF$_2$]

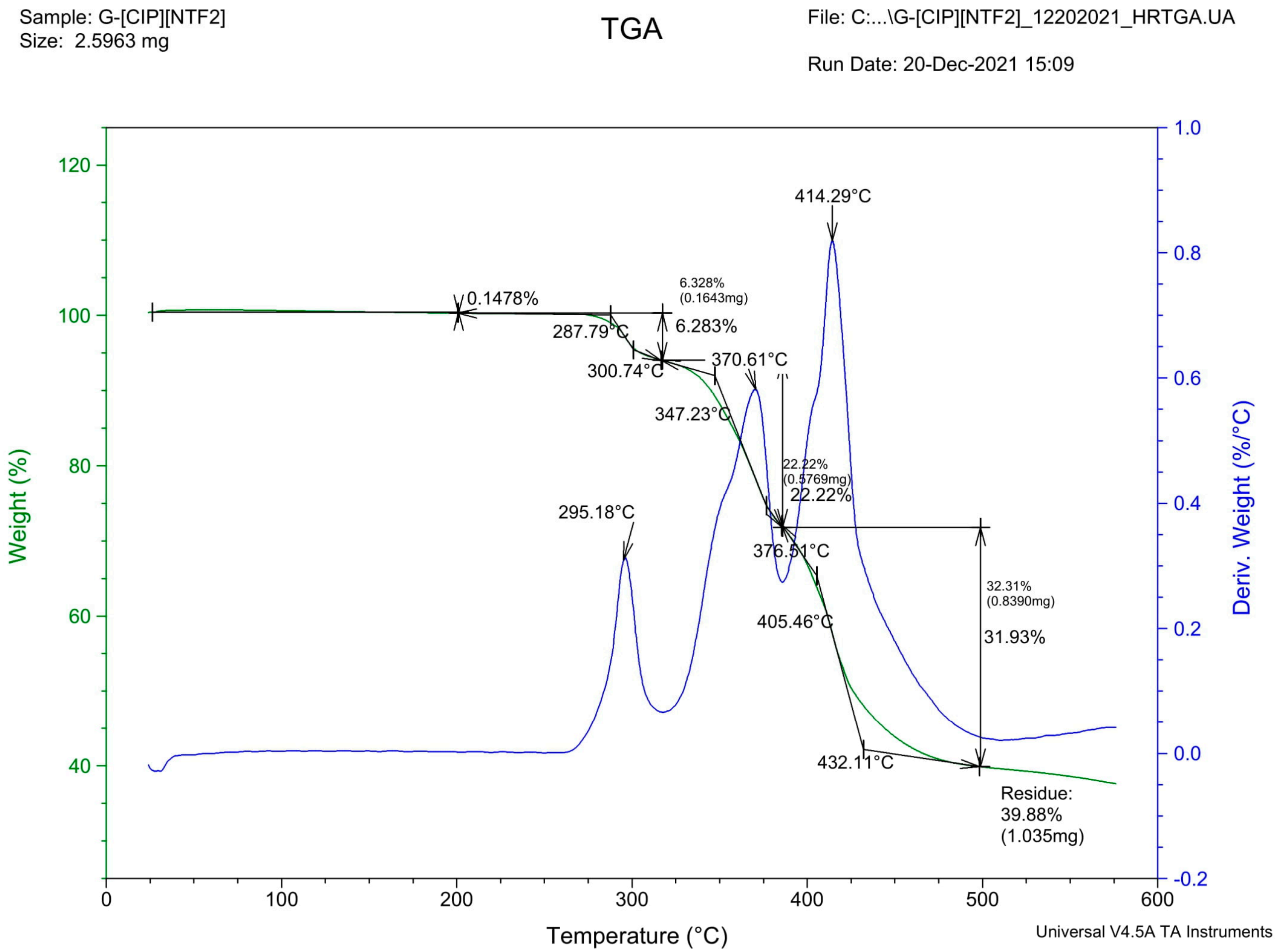

Sample: G-[CIP][NTF2]
Size: 2.5963 mg
TGA
File: C:...\G-[CIP][NTF2]_12202021_HRTGA.UA
Run Date: 20-Dec-2021 15:09
0.1478%
287.79°C
300.74°C
6.328%
(0.1643mg)
6.283%
370.61°C
347.23°C
414.29°C
295.18°C
22.22%
(0.5769mg)
22.22%
376.51°C
405.46°C
32.31%
(0.8390mg)
31.93%
432.11°C
Residue:
39.88%
(1.035mg)
Weight (%)
Deriv. Weight (%/°C)
Temperature (°C)
Universal V4.5A TA Instruments

[Cip][TPB]

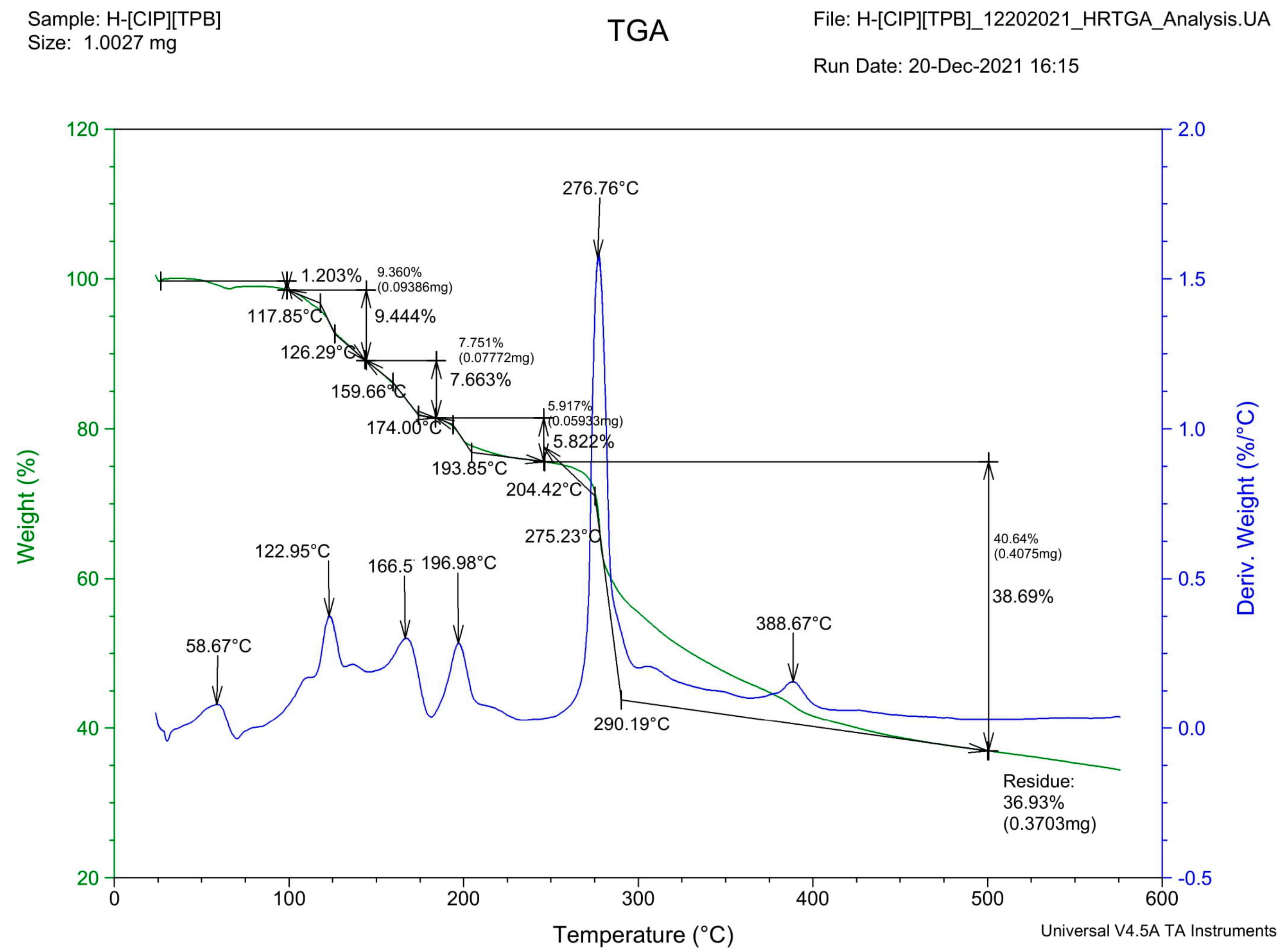

Sample: H-[CIP][TPB]
Size: 1.0027 mg
TGA
File: H-[CIP][TPB]_12202021_HRTGA_Analysis.UA
Run Date: 20-Dec-2021 16:15
276.76°C
1.203%
9.360%
(0.09386mg)
117.85°C
9.444%
126.29°C
7.751%
(0.07772mg)
7.663%
159.66°C
5.917%
(0.05933mg)
174.00°C
5.822%
193.85°C
204.42°C
275.23°C
122.95°C
166.5
196.98°C
40.64%
(0.4075mg)
38.69%
58.67°C
388.67°C
290.19°C
Residue:
36.93%
(0.3703mg)
Weight (%)
Deriv. Weight (%/°C)
Temperature (°C)
Universal V4.5A TA Instruments

[Cip][Doc]

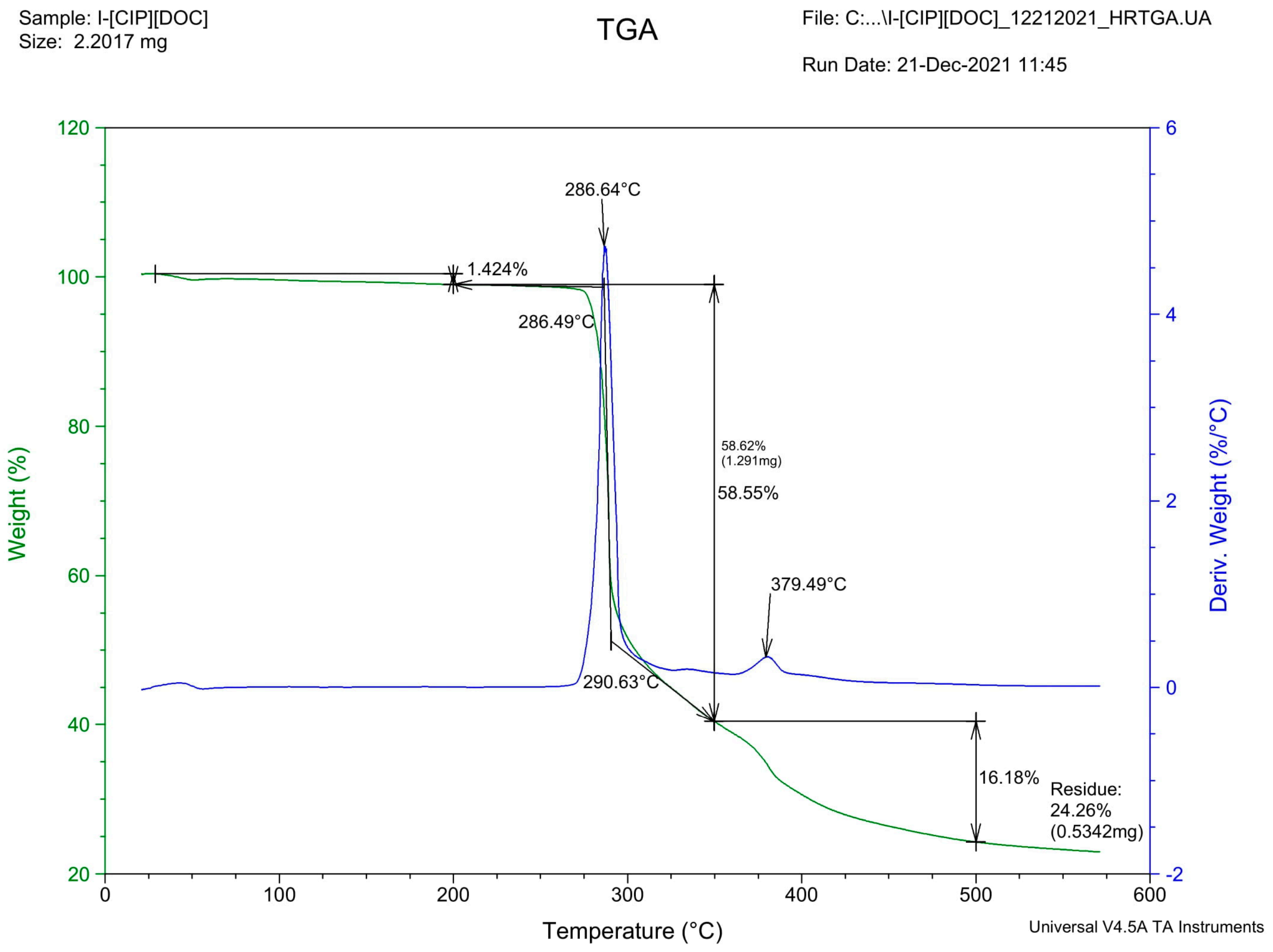

Sample: I-[CIP][DOC]
Size: 2.2017 mg
TGA
File: C:...\I-[CIP][DOC]_12212021_HRTGA.UA
Run Date: 21-Dec-2021 11:45
286.64°C
1.424%
286.49°C
58.62%
(1.291mg)
58.55%
379.49°C
290.63°C
16.18%
Residue:
24.26%
(0.5342mg)
Weight (%)
Deriv. Weight (%/°C)
Temperature (°C)
Universal V4.5A TA Instruments

[Cip][Dxc]

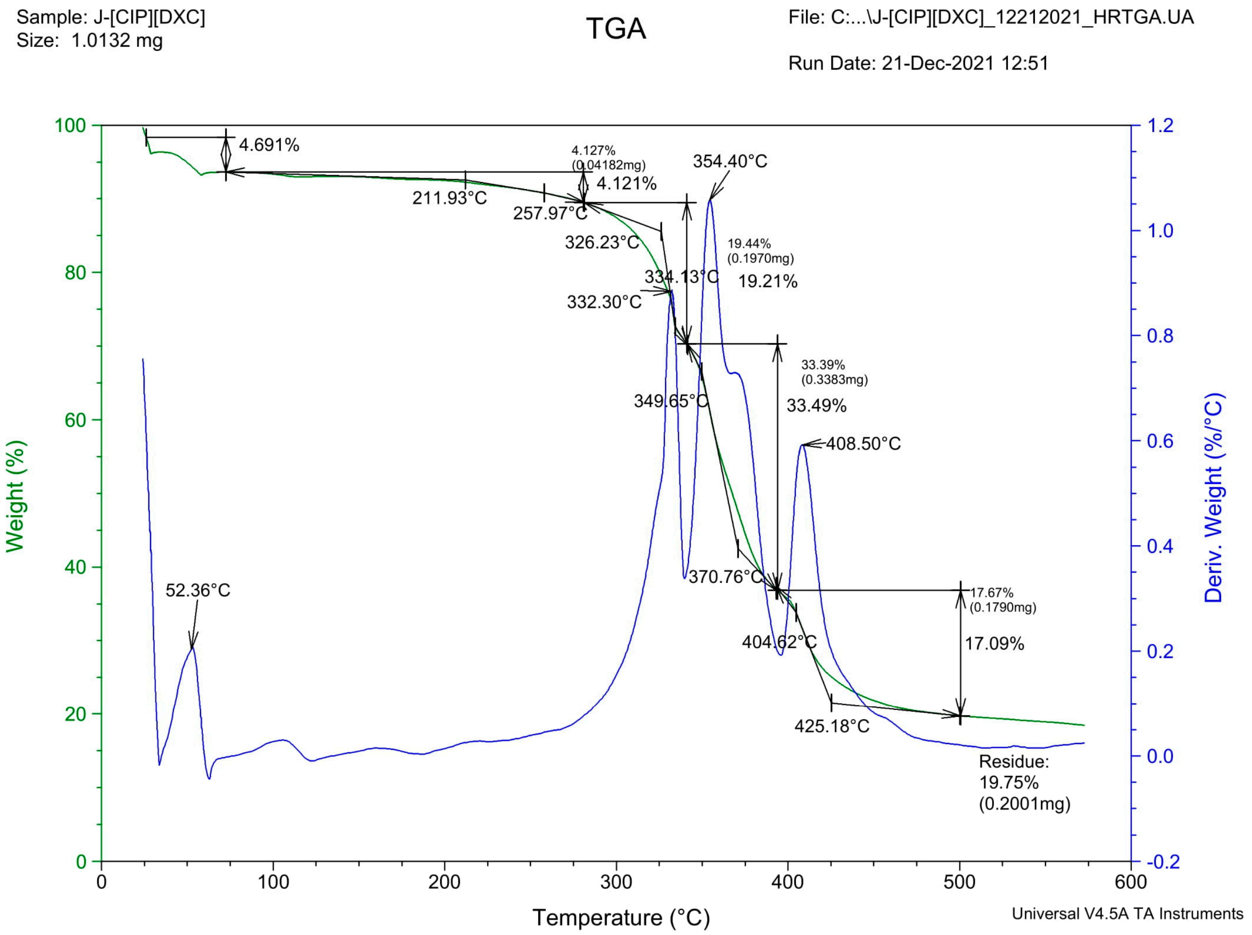

Sample: J-[CIP][DXC]
Size: 1.0132 mg
TGA
File: C:...\J-[CIP][DXC]_12212021_HRTGA.UA
Run Date: 21-Dec-2021 12:51
4.691%
4.127%
(0.04182mg)
4.121%
211.93°C
257.97°C
354.40°C
326.23°C
19.44%
(0.1970mg)
19.21%
334.13°C
332.30°C
33.39%
(0.3383mg)
33.49%
349.65°C
408.50°C
52.36°C
370.76°C
17.67%
(0.1790mg)
17.09%
404.62°C
425.18°C
Residue:
19.75%
(0.2001mg)
Weight (%)
Deriv. Weight (%/°C)
Temperature (°C)
Universal V4.5A TA Instruments

[Mox][BETI]

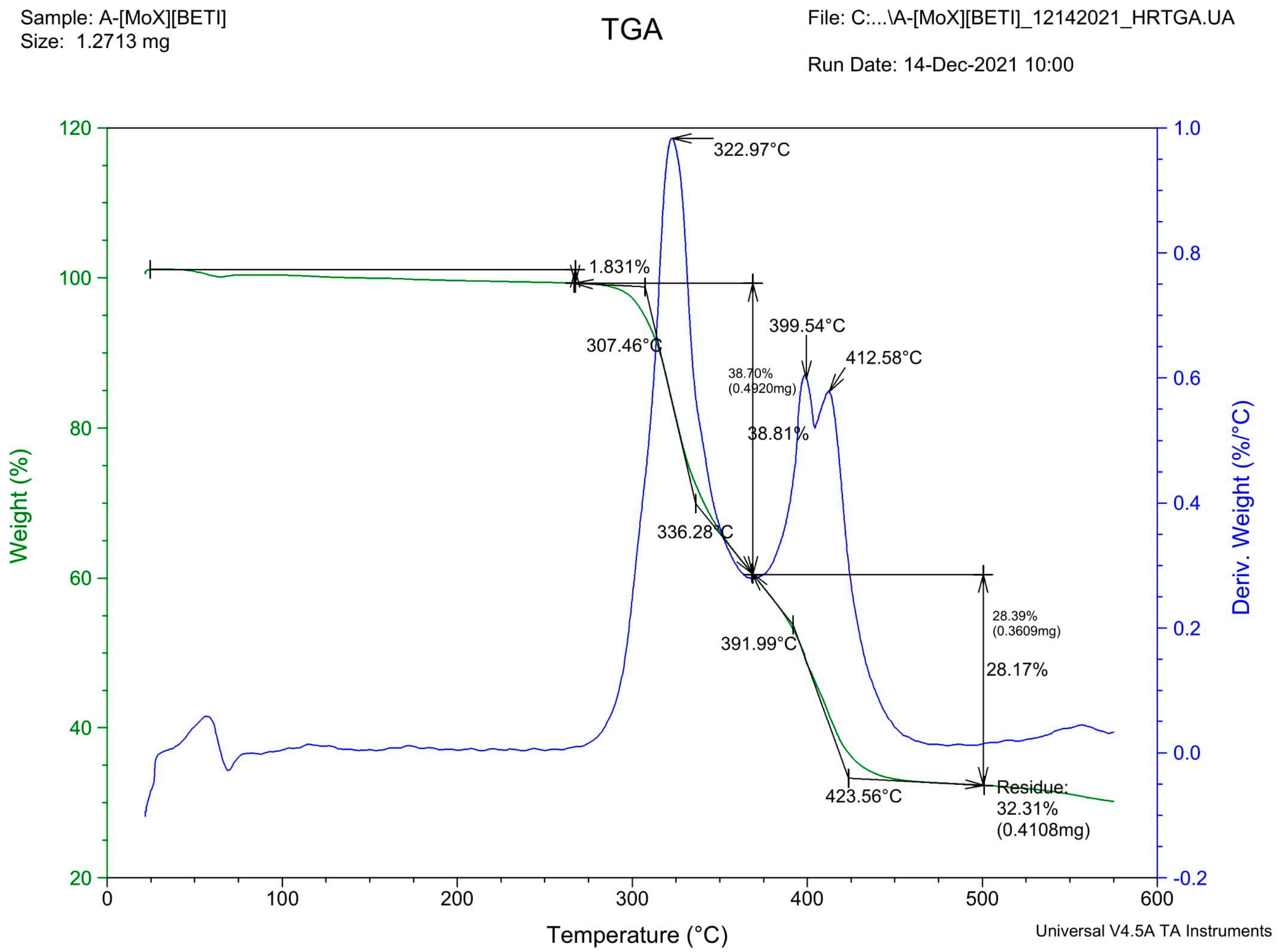

Sample: A-[MoX][BETI]
Size: 1.2713 mg
TGA
File: C:...\A-[MoX][BETI]_12142021_HRTGA.UA
Run Date: 14-Dec-2021 10:00
322.97°C
1.831%
399.54°C
412.58°C
307.46°C
38.70%
(0.4920mg)
38.81%
336.28°C
391.99°C
28.39%
(0.3609mg)
28.17%
423.56°C
Residue:
32.31%
(0.4108mg)
Weight (%)
Deriv. Weight (%/°C)
Temperature (°C)
Universal V4.5A TA Instruments

[Mox][NTF_2]

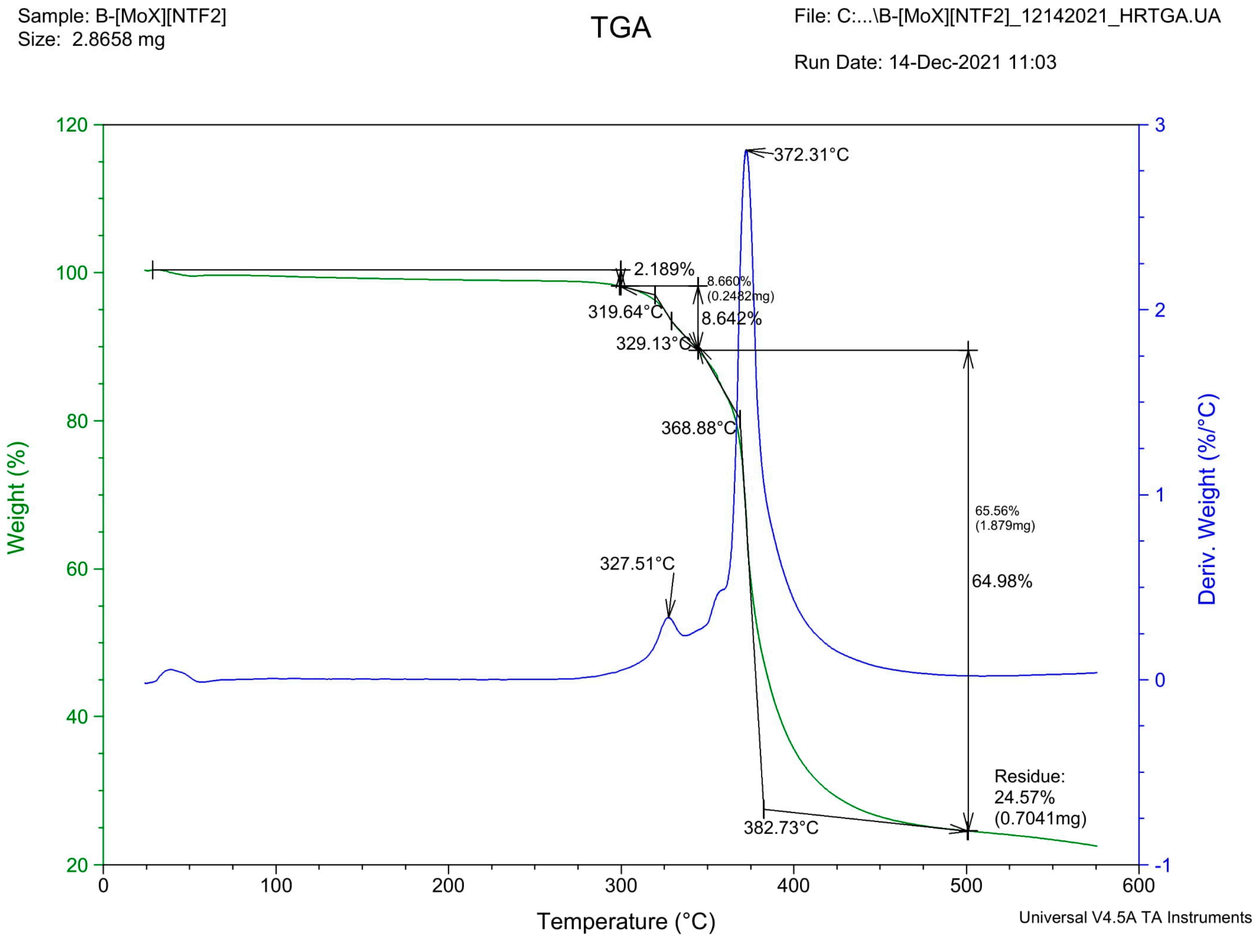

Sample: B-[MoX][NTF2]
Size: 2.8658 mg
TGA
File: C:...\B-[MoX][NTF2]_12142021_HRTGA.UA
Run Date: 14-Dec-2021 11:03
372.31°C
2.189%
8.660%
(0.2482mg)
319.64°C
8.642%
329.13°C
368.88°C
65.56%
(1.879mg)
64.98%
327.51°C
Residue:
24.57%
(0.7041mg)
382.73°C
Weight (%)
Deriv. Weight (%/°C)
Temperature (°C)
Universal V4.5A TA Instruments

[Mox][TPB]

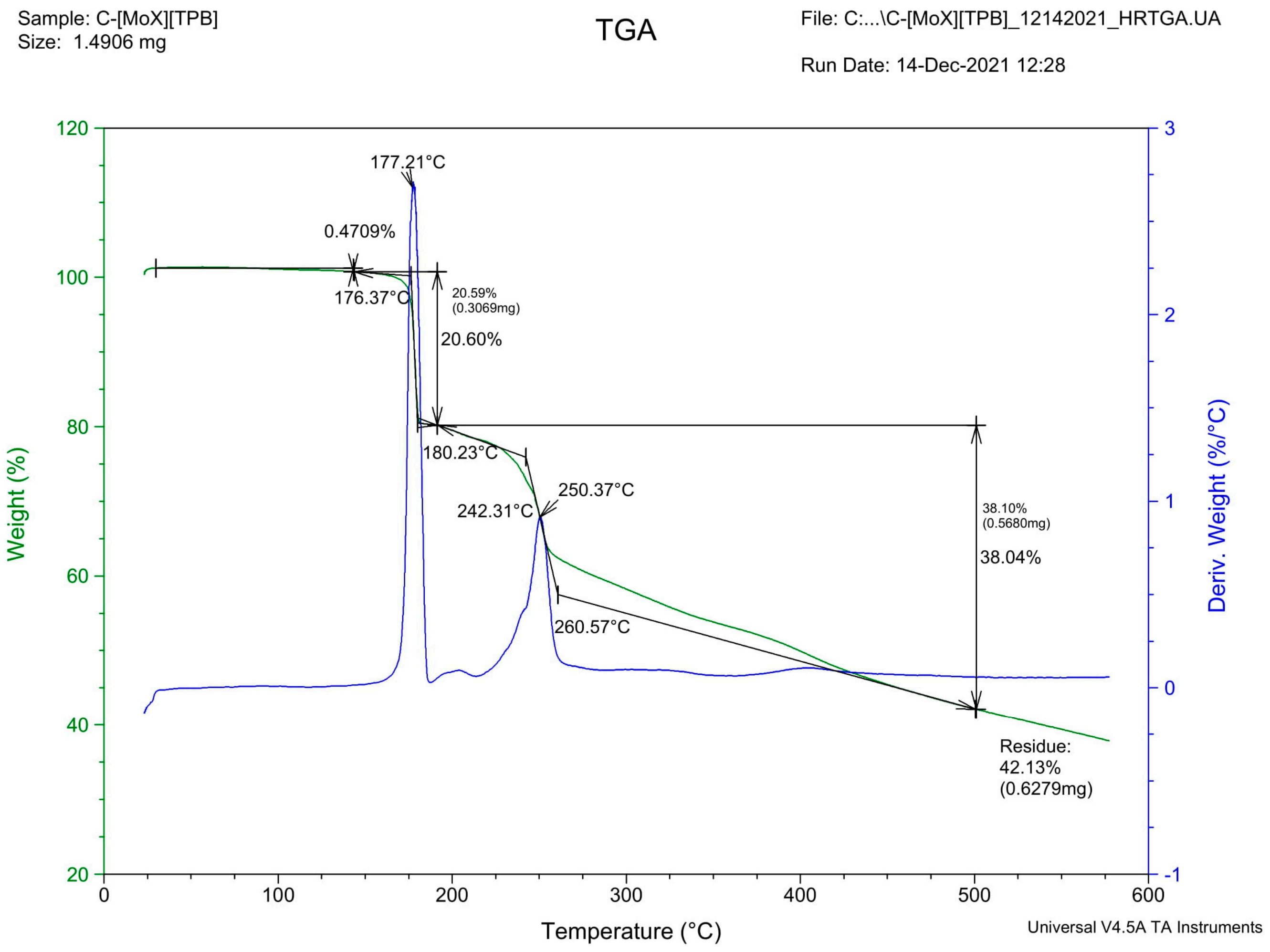

Sample: C-[MoX][TPB]
Size: 1.4906 mg
TGA
File: C:...\C-[MoX][TPB]_12142021_HRTGA.UA
Run Date: 14-Dec-2021 12:28
177.21°C
0.4709%
176.37°C
20.59%
(0.3069mg)
20.60%
180.23°C
250.37°C
242.31°C
38.10%
(0.5680mg)
38.04%
260.57°C
Residue:
42.13%
(0.6279mg)
Weight (%)
Deriv. Weight (%/°C)
Temperature (°C)
Universal V4.5A TA Instruments

[Mox][Doc]

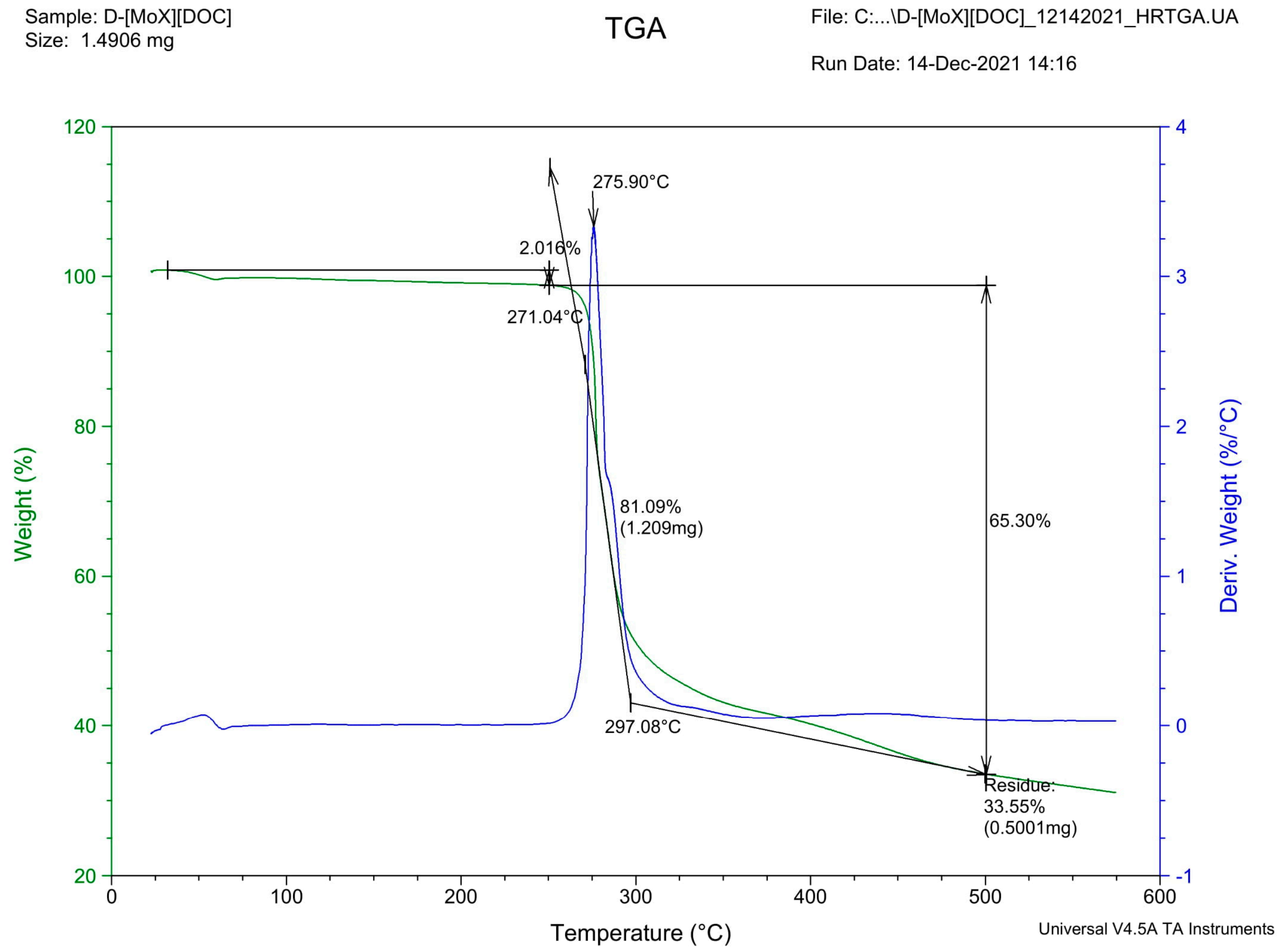

Sample: D-[MoX][DOC]
Size: 1.4906 mg
TGA
File: C:...\D-[MoX][DOC]_12142021_HRTGA.UA
Run Date: 14-Dec-2021 14:16
275.90°C
2.016%
271.04°C
81.09%
(1.209mg)
65.30%
297.08°C
Residue:
33.55%
(0.5001mg)
Weight (%)
Deriv. Weight (%/°C)
Temperature (°C)
Universal V4.5A TA Instruments
120
100
80
60
40
20
4
3
2
1
0
-1
0
100
200
300
400
500
600

[Mox][Dxc]

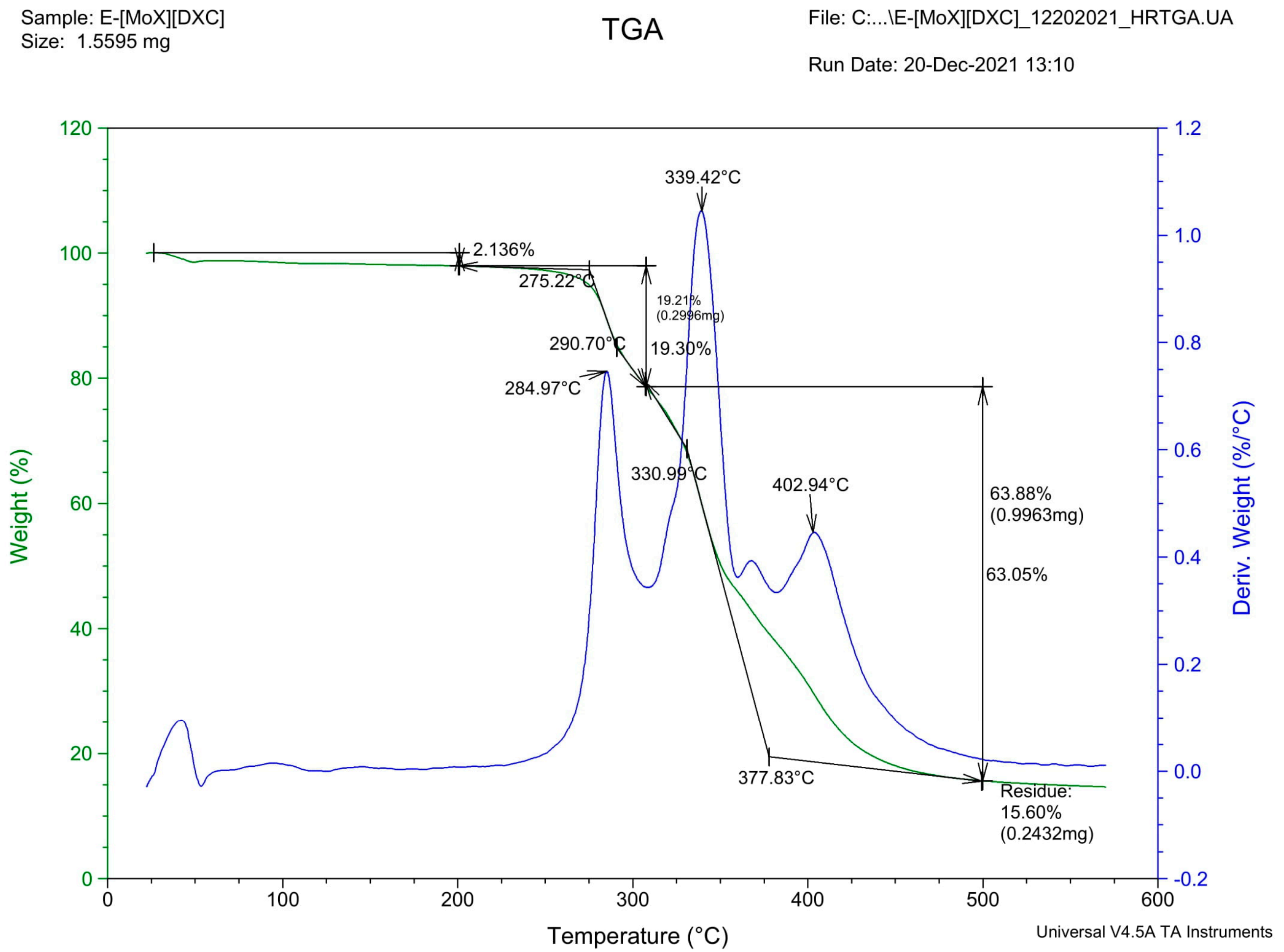

Sample: E-[MoX][DXC]
Size: 1.5595 mg
TGA
File: C:...\E-[MoX][DXC]_12202021_HRTGA.UA
Run Date: 20-Dec-2021 13:10
339.42°C
2.136%
275.22°C
19.21%
(0.2996mg)
290.70°C
19.30%
284.97°C
330.99°C
402.94°C
63.88%
(0.9963mg)
63.05%
377.83°C
Residue:
15.60%
(0.2432mg)
Weight (%)
Deriv. Weight (%/°C)
Temperature (°C)
Universal V4.5A TA Instruments
